# Simultaneous Analysis of 272 Pesticides in Agricultural Products by the QuEChERS Method and Gas Chromatography with Tandem Mass Spectrometry

**DOI:** 10.3390/molecules29092114

**Published:** 2024-05-03

**Authors:** Da-Young Yun, Ji-Yeon Bae, Yoon-Jung Kang, Chae-Uk Lim, Gui-Hyun Jang, Mi-Ok Eom, Won-Jo Choe

**Affiliations:** 1 Pesticides and Veterinary Drug Residues Division, National Institute of Food and Drug Safety Evaluation, Ministry of Food and Drug Safety, Cheongju 28159, Republic of Korea; dyyun96@korea.kr (D.-Y.Y.); jiyeon0962@korea.kr (J.-Y.B.); arion@korea.kr (G.-H.J.); miokeom@korea.kr (M.-O.E.); 2 Center for Food and Drug Analysis, Busan Regional Office of Food and Drug Safety, Busan 47537, Republic of Korea; chemi07@korea.kr; 3 Safety Analysis Division, Experiment Research Institute, National Agricultural Products Quality Management Service, Kimcheon 39660, Republic of Korea; chaiuk@korea.kr

**Keywords:** simultaneous analysis, pesticide residue, QuEChERS, GC-MS/MS

## Abstract

The aim of this study is to develop a rapid and accurate method for simultaneous analysis of multi-residue pesticides and conduct pesticide monitoring in agricultural products produced by the production and distribution stage in Korea. The representative agricultural products were selected as brown rice, soybean, potato, mandarin, and green pepper and developed using gas chromatography with tandem mass (GC-MS/MS) for the analysis of 272 pesticide residues. The experimental samples were extracted by the QuEChERS-EN method and then cleaned up by using d-SPE, including MgSO_4_ and primary secondary amine (PSA) sorbents. The established method was validated in accordance with Codex CAC-GL/40, and the limit of quantitation (LOQ) was determined to be 0.01 mg/kg. A total of 243 pesticides satisfied the guidelines in five samples at three levels with values of 60 to 120% (recovery) and ≤45% (coefficient of variation, CV). The remaining 29 pesticides did not satisfy the guidelines, and these pesticides are expected to be used as a screening method for the routine inspection of agricultural products. As a result of analyzing 223 agricultural products in South Korea by applying the simultaneous analysis method, none of the detected levels in the samples exceeded the standard values based on maximum residue limits (MRLs). The developed method in this study will be used to inspect residual pesticides in agricultural products, and it is anticipated to contribute to the distribution of safe agricultural products to consumers.

## 1. Introduction

Pesticides, essential agricultural materials used since ancient times, play a crucial role in efficiently controlling pests during crop cultivation. Thus they contribute toward enhancing the productivity and quality of agricultural products [1,2]. After World War II, inexpensive and effective organochlorine pesticides began to be produced, followed by various synthetic pesticides such as organophosphates and phenoxy acids [1]. Ideally, these pesticides decompose naturally through biological, chemical, and photo processes, but excessive use can result in their prolonged presence in the soil, leading to absorption by crops and their persistence in the environment [2]. In particular, some organochlorine pesticides classified as persistent organic pollutants (POPs), including dichlorodiphenyltrichloroethane (DDT) and dieldrin, persist in the environment, exhibit long half-lives persisting within the environment. They act as endocrine disruptors in animals and humans with effects that cascade through the food chain, posing toxic risks to both organisms. Moreover, highly toxic POPs can induce neurotoxicity by depleting neurotransmitters such as serotonin and dopamine, and thus, the issue of residual pesticides remains a significant social concern [3,4]. The Food and Agricultural Organization and the World Health Organization have established and overseen the acceptable daily intake and maximum residue limits (MRLs) for pesticides through CODEX to ensure food safety. In the CODEX Pesticide Residues in Food Online Database [5], MRLs are set for 243 pesticides. In contrast, Europe has established MRLs for 500 pesticides, whereas Japan has set MRLs for 781 pesticides. Furthermore, some pesticides do not have specified MRLs and are managed below 0.01 ppm in Europe, Japan, and Canada [6]. In line with safety management trends in each country, South Korea first introduced a Positive List System (PLS) in 2016, which uniformly applies a limit of 0.01 ppm to pesticides not established MRLs. This system was fully implemented for agricultural products in 2019 [7]. Some states/countries such as California, Senegal, and China have established monitoring programs and risk assessments to monitor pesticides remaining in food to protect food safety and public health [8,9,10]. Pesticide monitoring serves multiple purposes, such as managing unacceptable pesticide levels, estimating specific pesticide concentrations in agricultural products, and assessing overall safety. Ultimately, this ensures the provision of safe agricultural products to consumers [8]. Thus it is imperative to conduct thorough monitoring of residual pesticides in agricultural products and the development of simultaneous analysis methods that can monitor multiple pesticides quickly and accurately is essential.

According to the Korean Food Code, the simultaneous analysis method employed by the Ministry of Food and Drug Safety (MFDS) involves extracting samples with acetonitrile (MeCN) or acetone. For target pesticides analyzed using gas chromatography (GC), the extracts are purified with florisil or carbon cartridges, whereas those analyzed using liquid chromatography (LC) are purified with amino propyl cartridges. Traditional detectors, such as the electron capture detector, flame photometric detector, nitrogen phosphorous detector, fluorescence detector, and ultraviolet detector, have all been used for suitable pesticide analysis [11]. Nevertheless, when these approaches are employed in the evaluation of agricultural products, they involve an intricate sequence of procedures, including extraction, distribution, refinement, and device analysis. The specific steps involved in this process vary depending on the type of crop and pesticide analyzed. Consequently, this necessitates a substantial investment in time and financial resources, which in turn amplifies the potential for errors. Therefore, it is imperative to establish a simultaneous analysis method that aligns with the current trends in analytical techniques. Considering these trends, various studies have sought to find a method to analyze multiple pesticides simultaneously, using agricultural products such as fruits and vegetables. Commonly, these strategies rely on mass spectrometry (MS), which can detect low levels of pesticides in various matrices with excellent sensitivity and selectivity [12,13,14]. To quickly assess distributed agricultural products, there is a need for a rapid pre-treatment method that matches the speed of the analysis equipment. The quick, easy, cheap, effective, rugged, and safe (QuEChERS) method, first introduced by Anastassiades, is a method comprising only two steps: (1) extraction with MeCN and (2) liquid–liquid distribution and purification using dispersive solid-phase extraction (d-SPE). Thus, many studies employ this method because of its simple pretreatment process and shorter processing time [15].

The QuEChERS method was initially introduced as a liquid–liquid distribution method, involving the separation of an aqueous and an organic solution through the addition of MgSO_4_ and NaCl. In addition, the AOAC Official Method 2007.01 (QuEChERS-AOAC) of buffering with acetate salts and the European Committee for Standardization Standard Method EN 15662 (QuEChERS-EN) of buffering with citrate salts have been proposed for the excellent recovery of certain pH-dependent pesticides [16]. Some research conducted a simultaneous analysis of 360 pesticides in brown rice, orange, spinach, and potato using an analysis method that combines the QuEChERS method and GC-MS/MS [17]. The extraction process, using various extraction solvents and the QuEChERS method, achieved an excellent recovery rate of 338 components with the use of 0.1% formic acid in MeCN and the original QuEChERS method. However, given that the results obtained from the experiment using QuEChERS-EN method with MeCN as an extraction solvent are not significantly different, further investigation into the development of a simultaneous analysis method based on the buffered EN 15662 method is warranted. Therefore, this study attempted to establish a simultaneous analysis method based on the QuEChERS-EN method after establishing the instrumental analysis conditions for both parent compounds and metabolites of 272 pesticides using GC-MS/MS. Utilizing a multi-component analysis method, this study examined five agricultural products (brown rice, soybean, mandarin, potato, and green pepper) to verify their compliance with CODEX guideline CAC-GL/40-1993 [18] and to present an analysis method suitable for routine inspections for the safety management of agricultural products.

## 2. Results and Discussion

### 2.1. Optimization of GC-MS/MS Conditions

A total of 272 pesticides, including both parent compounds and metabolites, were analyzed using a capillary column, which has the advantage of a high separation capacity and shortened analysis time for routine inspections [19]. The DB-5MS UI (30 m × 0.25 mm × 0.25 μm) capillary column, known for its utility in multi-residue analysis, was selected for the experiment [20,21].

The mass range was set to 40–450 *m*/*z*, and a full scan was conducted for each standard solution (1 mg/L) to establish the MRM conditions. Based on the full-scan results, the precursor ion with the highest sensitivity and superior selectivity was selected as a priority, and the mass value was selected as ≥200 *m*/*z*, as far as was possible. The product ion scan was performed with the selected precursor ion using various collision energies. The product ion with the highest sensitivity was selected as the quantification ion, and the next-largest detected product ion was selected as the qualification ion.

Some pesticides with poor peak shapes or intensities were identified so a priming system was implemented to address and enhance the observed issues. Some analytes tend to remain or decompose at the active site of the liner during the sample injection stage in GC [22]. Consequently, a priming system is generally employed in which analytes, such as analyte protectants (APs) or spinach extracts, are injected multiple times during the initiation of the sequence. Several studies have reported the crucial role of priming in improving pesticide analysis [17,23]. Parallel to spinach extract, a leafy vegetable, we used green pepper extract in the priming system, resulting in improved peak shapes and chromatographic signals for the investigated pesticides. The injection mode was set to splitless to achieve a high sensitivity for the target pesticides and the temperature program exhibited an optimal peak shape and high separation capacity for all compounds within 35.5 min. The experimental pesticides and MRM conditions are listed in Table S1.

### 2.2. Optimization of Extraction and Purification

Brown rice, which is categorized as a dry sample, and green pepper, which is classified as a vegetable, were employed in the optimization of the extraction and purification methods [18,24]. MeCN, which is utilized as the extraction solvent in the QuEChERS method, was selected in study. The extraction efficiencies of 272 pesticides were evaluated at the 0.01 mg/kg level using the citrate salt-based QuEChERS-EN method among the three QuEChERS methods. The recoveries of chlorothalonil and dichlofluanid did not meet the guideline range of 60–120%. Specifically, chlorothalonil showed recoveries of 31.9% and 35.1% in brown rice and green pepper, respectively, whereas dichlofluanid was not detected in either crop. Additional experimental results obtained using the other QuEChERS methods also revealed insufficient recovery for both components. Additionally, the number of components meeting the guideline criteria for these two methods was lower than that observed with the EN method. The inadequate recovery of both components was attributed to the compromised stability of the standard solutions of chlorothalonil and dichlofluanid dissolved in MeCN. Considering the susceptibility of both components to decomposition under basic conditions, we inferred that a correction method involving the addition of an acid was necessary. In the case of dichlofluanid, no detection was observed in the MeCN analysis after 24 h, whereas chlorothalonil exhibited a decline in the recovery rate [25]. Furthermore, the modified QuEChERS method, which involves extraction with MeCN containing 1% formic acid, is applicable to chlorothalonil analysis. Thus, we anticipated that the analysis of dichlofluanid and chlorothalonil would be enhanced by employing an acidified extraction solvent in the modified QuEChERS method [26]. Based on these experimental results, we established the QuEChERS-EN method as the selected extraction method.

**Figure 1:**
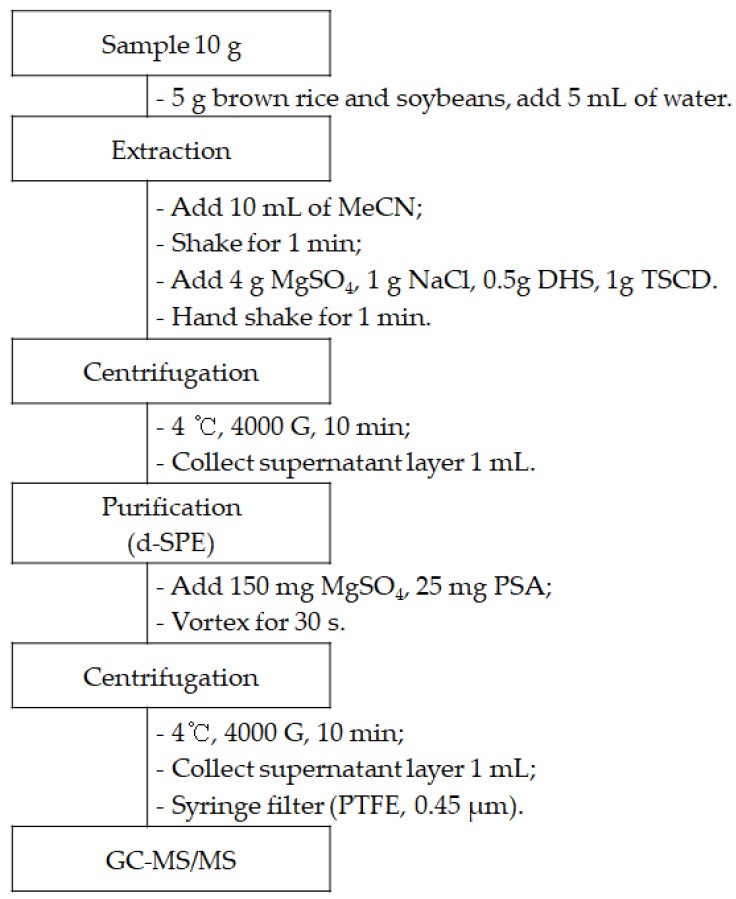
Experimental flow for 272 pesticides analysis in agricultural products.

To eliminate interfering substances that persisted in the organic solvent layer separated from the aqueous solution layer, we investigated the purification efficiency at a concentration of 0.01 mg/kg, using various d-SPE kits consisting of a primary secondary amine (PSA), octadecylsilane (C_18_), graphitized carbon black (GCB), and MgSO_4_. For pesticides meeting the guideline range of 60–120%, brown rice and green pepper treated with a d-SPE combination consisting of MgSO_4_ and PSA presented the highest number of components at 264 (97.1%) and 267 (98.2%), respectively (Table 1). We anticipated that the recovery rate would be favored with the combination of C_18_, known for its effectiveness in eliminating lipids, or GCB, recognized for its efficacy in removing carotenoids and chlorophyll, with PSA. PSA, with its ability to eliminate sugars, lipids, organic acids, and pigments, was expected to enhance the overall purification process [27]. Interestingly, when PSA alone was utilized, both brown rice and green pepper exhibited a high number of components that met the guideline criteria. Some pesticides with planar structures are adsorbed onto the lamellar structure of GCB, and similar studies have shown that recovery rates decrease in the purification process when using GCB and C_18_ compared to treatment with sole PSA [28,29]. Based on the above experimental results, a method for the simultaneous analysis of 272 pesticides was established, involving extraction using the QuEChERS-EN method and purification by d-SPE including MgSO_4_ and PSA (Figure 1).

### 2.3. Method Validation

**Figure 2:**
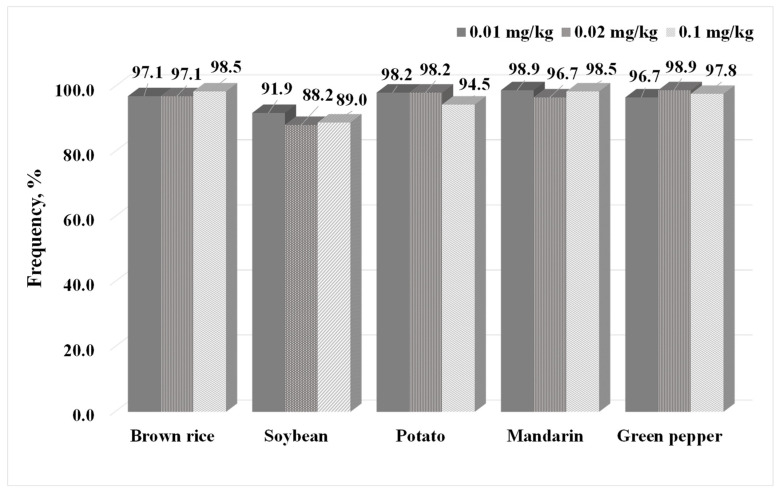
The percentage of pesticides meeting the guideline ranges for recovery (60–120% spiked at 0.01 and 0.02 mg/kg; 70–120% spiked at 0.1 mg/kg) and CV (≤45% at 0.01 and 0.02 mg/kg; ≤32% at 0.01 mg/kg), as determined through the utilization of an optimized analytical method.

To verify the efficiency and reliability of the established analytical method, we assessed the selectivity and linearity. Additionally, accuracy and precision were evaluated using repeatability and reproducibility. The results confirmed the absence of interfering substances at the same retention times as those of the 271 pesticides in the GC-MS/MS chromatograms of the five sample blanks (brown rice, soybean, mandarin, green pepper, and potato), thus confirming excellent selectivity. In the case of dichlofluanid, the peak was not properly separated to decomposition and matrix interference. Therefore, we determined the necessity to develop an individual method suitable for biochemical characterization, considering that dichlofluanid is not detectable in experiments involving spinach, brown rice, orange, and potato when using the pretreatment extraction method with MeCN containing 0.1% formic acid [17]. The coefficient of determination (R^2^) of all pesticides, except dichlofluanid, ≥0.99, confirming excellent linearity in the calibration range of 0.002–0.02 mg/kg. The LOD was set to a value with a signal-to-noise (S/N) ratio of at least 3, and all pesticides met the guideline range of 0.001–0.003 mg/kg, and thus, LOD was set 0.003 mg/kg. The LOQ was set at three times the LOD. A value of 0.01 mg/kg, corresponding to an S/N ratio of ≥10, was set as the LOQ, which was confirmed to meet the quantitative limit standard of the PLS. To evaluate the repeatability and reproducibility of the analysis method, the recovery and CV values were calculated by analyzing 5 and 11 repetitions at the LOQ, 2×LOQ, and 10×LOQ levels. The reproducibility of the five representative agricultural products, based on the standards of the CODEX guidelines, is shown in Figure 2.

The repeatability and reproducibility results for 272 pesticides in agricultural products are summarized in Tables S2–S6. When the established analysis method was cross-verified by three laboratories (n = 11), the ratio of pesticides satisfying the guideline ranges (recovery: 60–120%; CV: <45%) was 91.9–98.9% at 0.01 mg/kg, and those satisfying the guideline ranges (recovery: 70–120%; CV: <32%) were 88.6–98.9% at 0.02 mg/kg and 89.3–98.5% at 0.1 mg/kg. These findings indicate that most pesticides met the specified criteria. In particular, at the three tested concentrations, 13 pesticides (aldrin, anilofos, *γ*-BHC, butylate, chlorbenside, dichlofenthion, dichloran, fenclorim, fenoxanil, flucythrinate, indoxacarb, pyraclofos, and tridiphane) showed poor recoveries from soybean. Based on these findings, the amount of extraction solvent was increased from 10 to 20 mL, and consequently, the recovery and CV satisfied the guideline criteria (Table 2).

Among the 272 pesticides, 243 pesticides satisfied the CODEX guidelines for five representative agricultural products. These results suggest their suitability for routine inspections of residual pesticides in agricultural products. Additionally, extraction with 20 mL of MeCN is necessary for the accurate quantification of 13 pesticides in soybeans. Out of the 272 pesticides, 29 pesticides (2,6-DIPN, acetochlor, acrinathrin, bifenox, carfentrazone-ethyl, chinomethionat, chlorothalonil, cyfluthrin, cyhalofop-butyl, cyhalothrin, cypermethrin, DDT, dichlofluanid, dimethipin, dimethomorph, edifenphos, fenpropathrin, fenvalerate, fluvalinate, halfenprox, iprodione, molinate, nonachlor, ortho-phenyl phenol, permethrin, prochloraz, pyridalyl, quizalofop-ethyl, and tetramethrin) did not meet the guideline criteria. Acetochlor and prochloraz complied with the prescribed guidelines across all agricultural products and treatment concentrations. However, these metabolites did not meet the required standards. Consequently, quantification using this analytical method was deemed unfeasible, given that the analysis is conducted on the combined total of the parent compound and metabolites in accordance with the residual definition. Cyhalofop-butyl did not meet the guideline at the 0.01 and 0.02 mg/kg levels in potato. Consequently, quantification using this method is feasible for agricultural products, except potato. Using the developed simultaneous analysis method, accurate quantitative analysis of 243 pesticides is possible, and 29 pesticides can be applied to daily inspections for quick screening as qualitative compounds.

### 2.4. Matrix Effects

Although the developed method has the advantage of analyzing numerous pesticides within a short period, it is important to consider the matrix effects (MEs) when evaluating signals derived from pure solvents and blank matrix standards. To eliminate MEs, various purifications and corrections employing internal standards were considered. However, in the former case, the recovery of an analyte may be diminished during the matrix removal process, whereas in the latter case, drawbacks include economic concerns and the use of commercially unavailable materials. Therefore, preparing a calibration curve using a representative matrix of agricultural commodities can be a viable alternative [30,31,32]. An ME is classified as soft (−20 to 20%), medium (−50% to −20% or 20% to 50%), or strong (<−50% or >50%). The soft effect can be considered as a no-matrix effect. Thus, a matrix representing a soft effect in many pesticides can be selected as a representative matrix [33,34]. Taking this into consideration, the ME for the five agricultural products was calculated for 271 pesticides (299 compounds), excluding dichlofluanid, as shown in Tables S2–S6.

**Figure 3:**
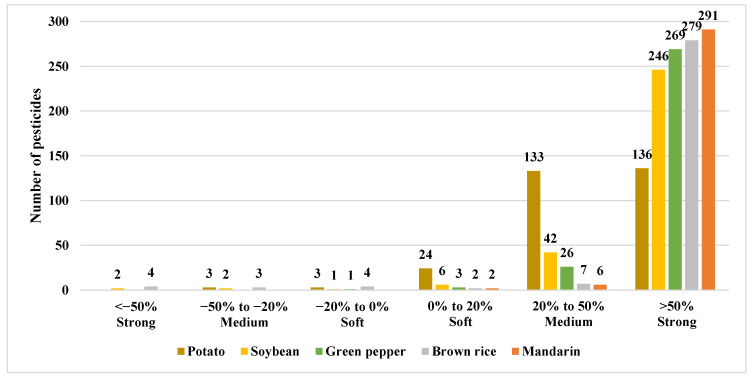
Matrix effect of five agricultural products, determined using the matrix-matched standard and solvent-only calibration curves.

According to the calculated results, among the 299 compounds, 136 (potato), 246 (soybean), 269 (pepper), 279 (brown rice), and 291 (mandarin) pesticides exhibited an ME greater than 50%. These results indicate that most of the pesticides demonstrated strong effects. In addition, the ME of 133 (potato), 42 (soybean), 26 (pepper), 7 (brown rice), and 6 (mandarin) pesticides fell within the range of 20–50% (medium effect), whereas 24 (potato), 6 (soybean), 3 (pepper), 2 (brown rice), and 2 (mandarin) exhibited a soft effect (Figure 3). Generating a calibration curve using a matrix-matched standard solution, rather than a solvent-only standard solution, is crucial for ensuring precise sample quantification in the overall results. However, none of the five crops seemed suitable as representative crops for constructing such a calibration curve. Thus, a subsequent study is necessary to identify a representative agricultural product matrix that exhibits a soft effect.

### 2.5. Application of the Multi-Residue Analysis Method in Real Samples

In total, 223 samples, including soybean, persimmon, mandarin, peach, potato, pepper, radish, Korean cabbage, apple, brown rice, onion, and tomato, were analyzed. Residual pesticides were detected above the 0.01 mg/kg level in 53 agricultural products (23.8%), and all samples remained within the established standards in accordance with the MRLs set by the Food Code of the Food and Drug Safety in Korea. Pesticide residues were detected in 9 samples of persimmon (45.0%), 7 samples of mandarin (36.8%), 6 samples of pepper (33.3%), 2 samples of radish (11.1%), 3 samples of Korean cabbage (16.7%), 7 samples of peach (36.8%), 12 samples of apple (66.7%), 2 samples of brown rice (11.1%), and 5 samples of tomato (27.8%) (Table 3). The most frequently detected pesticides were tebuconazole (14), chlorfenapyr (8), indoxacarb (8), difenoconazole (7), and spiromesifen (6). The monitoring results of agricultural products distributed in Korea suggest that the analysis method developed in this study can be effectively employed in daily inspections.

## 3. Materials and Methods

### 3.1. Chemicals and Reagents

High-performance liquid chromatography (HPLC)-grade water, acetone, MeCN, dichloromethane (DCM), methanol (MeOH), and n-hexane were purchased from Merck (Darmstadt, Germany). QuEChERS-EN method (Part No. ENK1-SC) and d-SPE clean-up (Part No. PM1EN) kits were supplied by Chromatific (Heidenrod, Germany). Polytetrafluoroethylene (PTFE) syringe filters (0.45 μm) were purchased from Thermo Fisher Scientific (Waltham, MA, USA).

### 3.2. Preparation of Analytical Standard

The target pesticides included 272 pesticides (300 compounds), encompassing fungicides, herbicides (safeners and synergists), insecticides (synergists), plant growth regulators, nematicides, and acaricides. The standards were purchased from Kemidas (Suwon, Republic of Korea), Dr. Ehrenstorfer (Augsburg, Germany), ChemService (West Chester, PA, USA), HPC Standards (Cunnersdorf, Germany), AccuStandard (New Haven, CT, USA), and Wako (Osaka, Japan). Each standard was prepared as a 100 mg/L stock solution using MeCN, MeOH, DCM, and n-hexane, taking solubility into consideration. The stock solutions were diluted in MeCN to prepare 5 mg/L of mixed stock solutions, which were subsequently stored at −20 °C, in the dark. These mixed stock solutions were further diluted with MeCN to prepare matrix-matched calibration standards (0.002–0.1 mg/L) using a blank matrix sample.

### 3.3. Sample Preparation

To validate the analytical method, blank samples that did not contain the target pesticides were obtained from local Korean markets. Twelve different agricultural products (n = 223)—soybean (21), persimmon (20), mandarin (19), peach (19), potato (18), pepper (18), radish (18), Korean cabbage (18), apple (18), brown rice (18), onion (18), and tomato (18)—were collected from different local markets to confirm the applicability of the analytical method. The specimens were collected between June and September from Seoul (33), Busan (25), Incheon (22), Daegu (21), Suwon (16), Daejeon (16), Gwangju (16), Ulsan (15), Cheongju (14), Changwon (13), Jeonju (12), Wonju (10), and Jeju (10). Each sample (>1 kg) was homogenized in accordance with the Korea Food Code [11] and stored in a container at −20 ℃ until further study.

Samples (10 g) were extracted with MeCN (10 mL) and shaken for 1 min. Dry samples, such as brown rice and soybeans, were weighed (5 g) and wetted with 5 mL of deionized water before extraction. The QuEChERS kit—4 g of anhydrous magnesium sulfate, 1 g of sodium chloride, 0.5 g of disodium hydrogen citrate sesquihydrate, and 1 g of trisodium citrate dehydrate—was added to the mixture. The mixture was then shaken for 1 min and centrifuged at 4000× *g* for 10 min. The supernatant (1 mL) was transferred to a d-SPE clean-up kit (150 mg anhydrous magnesium sulfate and 25 mg PSA), shaken for 30 s, and centrifuged at 4000× *g* for 10 min. The supernatant was passed through a syringe filter and analyzed by GC-MS/MS.

### 3.4. Optimization of Analytical GC-MS/MS Conditions

Multi-residue pesticide analysis was performed using an Agilent Technologies 7890 B GC system combined with a 7010 triple quadrupole mass spectrometer (Agilent Technologies, Santa Clara, CA, USA). Data acquisition and processing were conducted using Agilent MassHunter QQQ Acquisition and Quantitative Analysis software, version 10.1. A DB-5MS UI (30 m × 0.25 mm × 0.25 μm) capillary column (Agilent Technologies) was employed to separate 300 pesticides, using helium (99.9% purity) as the carrier and quenching gas, and nitrogen (99.9% purity) as the collision gas. The collision and quenching gas flows were set to 1.2 mL/min and 2.25 mL/min, respectively, and 1 μL of sample/standard was injected in spitless mode. The oven temperature program was set as follows: initially at 60 °C and increased to 180 °C at 20 °C/min, then increased to 300 °C at 5 °C/min and held for 5 min. The ion source and transfer line temperatures were both set at 280 °C. The electron ionization energy was set at −70 eV and the detector voltage at 1.4 kV. Additionally, a solvent delay of 3.5 min was implemented, so that the total run time was 35.5 min (Table 4).

For the MRM setup, each pesticide was diluted to 1 mg/L with MeCN and subjected to a full scan in the 40–50 *m*/*z* range to determine the retention time. The precursor ion was selected from the full-scan mass spectrum, and the product ion and optimal collision energy were selected from the product scan.

### 3.5. Method Validation

Method validation was conducted on the five representative agricultural products (brown rice, soybean, mandarin, green pepper, and potato) in accordance with CODEX Alimentarius guideline CAC/GL 40 [18]. The validation encompassed measurements of linearity, selectivity, limit of detection (LOD), limit of quantitation (LOQ), accuracy, and precision (repeatability and reproducibility). Linearity was confirmed through a matrix-matched calibration curve using the standard mixture (0.002–0.2 mg/L) diluted with blank samples (≥90%, *v*/*v*). The coefficient of determination (R^2^) was calculated to assess the linearity. To evaluate the selectivity of the analytical method, spiked blank samples were compared with non-spiked samples, to confirm that the interference occurred at the same retention time for each pesticide. The signal-to-noise (S/N) ratios for the LOD and LOQ exceeded 3 and 10, respectively, in the chromatogram. The standard stock solution was prepared in MeCN (5 mg/L) and spiked into blank samples to evaluate the repeatability (n = 5) and reproducibility (n = 11). The spiking levels in the agricultural products were LOQ, 2×LOQ, and 10×LOQ. Accuracy and precision were calculated using the mean recovery (%) and coefficient of variation (CV, %), respectively.

To evaluate the ME (%) of pesticides in the GC-MS/MS instrument, which may exhibit suppression or enhancement phenomena, a matrix-matched standard was prepared using the five agricultural products. The ME (%) was calculated by comparing the slope of the matrix-matched standard with that based on solvent-only calibration, using the following formula:

$$\mathrm{ME}\;(\%)=\left[\frac{\mathrm{Slope}\mathrm{of}\mathrm{spiked}\;\mathrm{in}\mathrm{matrix}\;\mathrm{matched}\mathrm{calibration}\mathrm{curve}}{\mathrm{Slope}\mathrm{of}\mathrm{spiked}\;\mathrm{in}\;\mathrm{solvent}\;\mathrm{calibration}\;\mathrm{curve}}-1\right]\;\text{×}\;100$$

## 4. Conclusions

In this study, we developed a QuEChERS-based method for the simultaneous analysis of 272 pesticides in agricultural products using GC-MS/MS. The MeCN served as the solvent for extracting multicomponent pesticides, and the QuEChERS-EN extraction method was established. A clean-up procedure was developed to remove impurities present in the matrix. This involved using dispersive solid-phase clean-up procedure with primary secondary amines, which led to excellent recovery and CV results. Following the CODEX guidelines, we assessed the linearity, selectivity, repeatability, reproducibility, and LOQ for five agricultural products (brown rice, soybean, mandarin, potato, and green pepper) across three concentration levels. The results confirmed that 243 pesticides satisfied the guidelines at each concentration level, affirming that the simultaneous analysis method can be utilized as a quantitative test method. Additionally, the 29 pesticides that did not meet the guidelines could be used as a screening method for routine inspection purposes. A total of 223 samples were inspected using the developed analysis method in accordance with the MRLs established by the MFDS, whereby 58 agricultural products (23.8%) were detected. Thus, the developed method proves to be suitable for the daily inspection in agricultural products and it will be employed to confirm the MRLs established in South Korea.

## Figures and Tables

**Table 1:** Number and percentages of pesticides meeting guideline criteria (recoveries: 60–120%; CV: ≤30%) at 0.01 mg/kg in brown rice and green pepper using various d-SPE sorbents.

d-SPE Sorbents	Brown Rice		Green Pepper	
	No. of Analytes	% of Analytes	No. of Analytes	% of Analytes
150 mg of MgSO_4_, 25 mg of PSA	264	97.1	267	98.2
150 mg of MgSO_4_, 25 mg of PSA, 25 mg of C_18_	261	96.0	264	97.1
150 mg of MgSO_4_, 25 mg of PSA, 2.5 mg of GCB	262	96.3	257	94.5

**Table 2:** Improvement of recovery efficiency (%) of 13 pesticides using 20 mL of extraction solvent in soybean spiked at 0.01, 0.02, and 0.1 mg/kg.

No.	Analyte	Soybean (*n* = 11)					
		0.01 mg/kg		0.02 mg/kg		0.1 mg/kg	
		Rec. (%)	CV (%)	Rec. (%)	CV (%)	Rec. (%)	CV (%)
1	Aldrin	85.9	7.9	79.9	3.9	90.0	4.9
	Dieldrin	69.6	2.6	114.2	2.7	119.0	4.5
2	Anilofos	88.9	8.3	106.4	4.3	101.9	4.8
3	BHC, *γ*-	76.9	9.2	89.6	4.9	88.6	12.3
4	Butylate	104.0	6.0	71.7	5.8	74.8	13.7
5	Chlorbenside	92.9	7.8	112.7	4.2	86.2	2.3
6	Dichlofenthion	62.3	9.3	72.9	3.0	82.3	18.6
7	Dicloran	112.2	4.6	114.8	9.8	110.7	8.3
8	Fenclorim	87.3	15.2	77.7	8.1	76.1	12.0
9	Fenoxanil	84.2	6.8	107.3	5.6	102.0	2.6
10	Flucythrinate-1	90.8	7.3	105.7	4.1	100.8	2.6
	Flucythrinate-2	90.7	7.7	108.3	3.7	101.3	2.2
11	Indoxacarb	75.2	9.9	95.0	5.8	97.9	1.6
12	Pyraclofos	86.8	9.8	114.9	3.8	109.6	11.3
13	Tridiphane	70.4	9.7	80.7	6.2	85.6	18.7

**Table 3:** Monitored pesticide results from agricultural products in South Korea.

Commodity	Sample Number	Detected Number	Pesticides	Concentration	MRLs (Korea)
				(mg/kg)	
Persimmon	20	9	Buprofezin	0.015	0.5
			Difenoconazole	0.012	1
			Difenoconazole	0.017	1
			Difenoconazole	0.048	1
			Tebuconazole	0.017	2
			Cyprodinil	0.02	1
			Tebuconazole	0.031	2
			Buprofezin	0.018	0.5
			Tebuconazole	0.078	2
			Trifloxystrobin	0.046	0.7
			Buprofezin	0.034	0.5
			Tebuconazole	0.021	2
			Buprofezin	0.01	0.5
Mandarin	19	7	Chlorfenapyr	0.016	1
			Indoxacarb	0.034	0.5
			Chlorfenapyr	0.07	1
			Deltamethrin	0.011	0.5
			Etoxazole	0.025	1
			Deltamethrin	0.017	0.5
			Chlorfenapyr	0.01	1
			Boscalid	0.015	0.5
			Chlorfenapyr	0.017	1
Pepper	18	6	Chlorfenapyr	0.016	1
			Deltamethrin	0.03	0.2
			Bifenthrin	0.021	1
			Boscalid	0.023	3
			Chlorfenapyr	0.222	1
			Chlorfenapyr	0.034	1
			Procymidone	0.015	5
			Boscalid	0.011	3
			Indoxacarb	0.014	1
			Chlorfenapyr	0.062	1
			Indoxacarb	0.024	1
			Spiromesifen	0.028	3
			Tebufenpyrad	0.026	0.5
Radish	18	2	Metalaxyl	0.019	0.05
			Tebuconazole	0.03	0.2
Korean cabbage	18	3	Diniconazole	0.018	0.1
			Metalaxyl	0.014	0.2
			Diniconazole	0.038	0.1
Peach	19	7	Indoxacarb	0.014	1
			Fenitrothion	0.045	0.1
			Boscalid	0.019	1
			Deltamethrin	0.039	0.5
			Difenoconazole	0.026	2
			Indoxacarb	0.678	1
			Difenoconazole	0.028	2
			Indoxacarb	0.011	1
			Trifloxystrobin	0.061	2
			Kresoxim- methyl	0.024	1
			Indoxacarb	0.03	1
Apple	18	12	Tebuconazole	0.023	1
			Bifenthrin	0.013	0.5
			Tebuconazole	0.032	0.5
			Tebuconazole	0.048	1
			Bifenthrin	0.015	0.5
			Deltamethrin	0.019	0.5
			Propiconazole	0.069	1
			Spiromesifen	0.101	1
			Tebuconazole	0.125	1
			Trifloxystrobin	0.011	0.7
			Chlorpyrifos	0.287	1
			Difenoconazole	0.049	1
			Indoxacarb	0.023	0.3
			Propiconazole	0.033	1
			Tebuconazole	0.029	1
			Tebuconazole	0.016	1
			Trifloxystrobin	0.014	0.7
			Bifenthrin	0.043	0.5
			Difenoconazole	0.011	1
			Tebuconazole	0.091	1
			Trifloxystrobin	0.043	0.7
			Tebuconazole	0.069	1
			Tebuconazole	0.017	1
Brown rice	18	2	Fenoxanil	0.013	1
			Fenoxanil	0.02	1
Tomato	18	5	Fenpyrazamine	0.012	3
			Spiromesifen	0.016	1
			Buprofezin	0.035	3
			Spiromesifen	0.015	1
			Spiromesifen	0.014	1
			Spiromesifen	0.019	1

**Table 4:** Analytical conditions for the GC-MS/MS of pesticides.

**Instrument**			
GC	7890B GC system (Agilent Technologies, Santa Clara, CA, USA)		
MS/MS	GC/MS Triple Quad (Agilent Technologies, Santa Clara, CA, USA)		
**GC conditions**			
Column	DB-5MS (30 m × 0.25 mm, 0.25 μm)		
Flow rate	1.2 mL/min (He 99%)		
Injection volume	1 μL		
Injection mode	splitless		
Oven temp.	Rate (°C/min)	Temperature (°C)	Hold (min)
	Initial	60	-
	20	180	-
	5	300	5
**MS/MS condition**			
Ionization mode	Electron ionization (EI)		
Transfer line temp.	280 °C		
Ion source temp.	280 °C		

## Data Availability

The data presented in this study are available on request from the corresponding author.

## Supplementary Materials

The following supporting information can be downloaded at https://www.mdpi.com/article/10.3390/molecules29092114/s1, Table S1: The optimized GC-MS/MS parameter including to each pesticide, retention time, molecular weight, exact mass, MRM transitions, and collision energies; Table S2: Method validation parameter of 272 pesticides in brown rice; Table S3: Method validation parameter of 272 pesticides in soybean; Table S4: Method validation parameter of 272 pesticides in potato; Table S5: Method validation parameter of 272 pesticides in mandarin; Table S6: Method validation parameter of 272 pesticides in green pepper.
