# Supplementary material for: Simultaneous Analysis of 272 Pesticides in Agricultural Products by the QuEChERS Method and Gas Chromatography with Tandem Mass Spectrometry
Source: Molecules. 2024 May 3;29(9):2114. doi: 10.3390/molecules29092114 (PMC11085925; doi:10.3390/molecules29092114)
Supplement: Supplementary file 1 [file molecules-29-02114-s001.zip › molecules-2979958-supplementary.pdf]

molecules

Supplementary Material

Simultaneous Analysis of 272 Pesticides in Agricultural Products by the QuEChERS Method and Gas Chromatography with Tandem Mass Spectrometry

Da-Young Yun [1], Ji-Yeon Bae [1], Yoon-Jung Kang [2], Chae-Uk Lim [3], Gui-Hyun Jang [1], Mi-Ok E om [1] and Won-Jo Choe [1,*]

[1] Pesticides and Veterinary Drug Residues Division, National Institute of Food and Drug Safety Evaluation, Ministry of Food and Drug Safety, Cheongju 28159, Republic of Korea; dyyun96@korea.kr (D.-Y.Y.);
jiyeon0962@korea.kr (J.-Y.B.); arion@korea.kr (G.-H.J.); miokeom@korea.kr (M.-O.E.)
[2] Center for Food and Drug Analysis, Busan Regional Office of Food and Drug Safety,
Busan 47537, Republic of Korea; chemi07@korea.kr
[3] Safety Analysis Division, Experiment Research Institute, National Agricultural Products Quality
Management Service, Kimcheon 39660, Republic of Korea; chaiuk@korea.kr
* Correspondence: aragaya06@korea.kr; Tel.: +82-43-719-4206

Supplementary Information

Table S1. The optimized GC-MS/MS parameter including to each pesticide, retention time, molecular weight, exact mass, MRM transitions, and collision energies.

S#	Compound name	Type of pesticide	RT (min.)	Molecular Weight (g/mol)	Extract Mass (g/mol)	Precursor ion (m/z)	Product ion (m/z)	CE (V)
1	2,6-Diisopropylnaphthalene (2,6-DIPN)	Plant growth regulator	9.06	212.3	212.1	212	197	15
						197	155	10
2	Acetochlor	Herbicide	10.76	269.8	269.1	223	146	10
							132	20
	2-ethyl-6-methylaniline (EMA)	Metabolite	5.14	135.2	135.1	120	77	20
						135	120	10
	2-1-hydroxyl-ethyl-6-methyl-aniline (HEMA)	Metabolite	6.28	151.2	151.0	151	117	40
							132	20
3	Acrinathrin	Insecticide, Acaricide	21.70	541.4	541.1	208	181	5
						289	93	10
4	Alachlor	Herbicide	10.99	269.8	269.1	188	160	10
						160	130	30
5	Aldrin	Insecticide	12.18	364.9	361.8	263	193	40
							191	40
	Dieldrin	Metabolite	15.20	380.9	377.8	263	193	40
						265	193	40
6	Allidochlor	Herbicide	5.38	173.6	173.0	134	56	5
						132	56	5
7	Ametryn	Herbicide	11.18	227.3	227.1	227	185	5
							170	10

S#	Compound name	Type of pesticide	RT (min.)	Molecular Weight (g/mol)	Extract Mass (g/mol)	Precursor ion (m/z)	Product ion (m/z)	CE (V)
8	Anilofos	Herbicide	19.85	367.8	367.0	226	157	15
							184	5
9	Aramite	Insecticide, Acaricide	15.77	334.9	334.1	187	65	10
						185	63	10
10	Aspon	Insecticide	11.93	378.4	378.0	211	115	10
							97	35
11	Atrazine	Herbicide	9.21	216.7	215.0	215	200	10
							58	15
12	Azaconazole	Fungicide	15.39	300.1	299.0	219	175	15
						217	173	15
13	Benfluralin	Herbicide	8.31	335.3	335.1	292	160	20
							264	10
14	Benfuresate	Herbicide	10.62	256.3	256.0	256	163	5
						163	121	5
15	Benodanil	Fungicide	16.68	323.1	322.9	231	203	20
							76	25
16	Benzoylprop-ethyl	Herbicide	18.78	366.2	365.0	292	105	10
						105	77	10
17	BHC, *α*-	Insecticide	8.77	290.8	287.8	181	145	15
						217	181	5
	BHC, *β*-	Insecticide	9.25	290.8	287.8	181	145	15
						217	181	5
	BHC, *δ*-	Insecticide	10.07	290.8	287.8	181	145	15
						217	181	5

S#	Compound name	Type of pesticide	RT (min.)	Molecular Weight (g/mol)	Extract Mass (g/mol)	Precursor ion (m/z)	Product ion (m/z)	CE (V)
18	BHC, γ- (Lindane)	Insecticide	9.47	290.8	287.8	181	145	15
						217	181	5
19	Bifenox	Herbicide	19.89	342.1	341.0	341	311	10
							189	20
20	Bifenthrin	Insecticide, Acaricide	19.41	422.9	422.1	181	166	15
							165	30
21	Boscalid	Fungicide	24.34	343.2	342.0	140	76	25
							112	10
22	Bromobutide	Herbicide	10.81	312.3	311.0	118	117	10
						232	176	10
23	Bromophos-methyl	Insecticide	12.67	366.0	363.8	331	316	20
						329	314	15
24	Bromophos-ethyl	Insecticide	13.91	394.0	391.8	303	211	30
							63	30
25	Bromopropylate	Acaricide	19.37	428.1	425.9	343	185	15
						339	183	15
26	Bupirimate	Fungicide	15.31	316.4	316.1	273	193	10
							108	10
27	Buprofezin	Insecticide, Acaricide	15.32	305.4	305.1	172	57	15
						175	132	15
28	Butachlor	Herbicide	14.22	311.9	311.1	237	160	10
						176	147	10
29	Butafenacil	Herbicide	23.41	474.8	474.0	331	180	20
							124	40

S#	Compound name	Type of pesticide	RT (min.)	Molecular Weight (g/mol)	Extract Mass (g/mol)	Precursor ion (m/z)	Product ion (m/z)	CE (V)
30	Butralin	Herbicide, Plant growth regulator	12.56	295.3	295.1	266	174	20
							220	15
31	Butylate	Herbicide	6.36	217.4	217.3	156	57	5
						146	57	10
32	Cadusafos	Insecticide, Nematicide	8.56	270.4	270.0	159	97	20
						158	97	20
33	Carbophenothion	Insecticide, Acaricide	17.30	342.9	341.9	344	159	5
						342	157	5
34	Carboxin	Fungicide	15.32	235.3	235.0	235	143	10
							87	20
35	Carfentrazone-ethyl	Herbicide	17.24	412.2	411.0	340	312	10
						312	151	20
36	Chinomethionat	Fungicide	13.99	234.3	233.9	234	206	5
						206	148	15
37	Chlorbenside	Insecticide	13.97	269.2	267.9	268	125	10
						125	89	15
38	Chlorbufam	Herbicide	9.21	223.7	223.0	223	53	15
							127	15
39	Chlordane-*cis*	Insecticide	14.36	409.8	405.7	375	266	20
						373	266	20
	Chlordane-*trans*	Insecticide	13.97	409.8	405.7	375	266	20
						373	266	20
40	Chlorethoxyfos	Insecticide	7.88	336.0	333.8	153	97	15
						97	47	35

S#	Compound name	Type of pesticide	RT (min.)	Molecular Weight (g/mol)	Extract Mass (g/mol)	Precursor ion (m/z)	Product ion (m/z)	CE (V)
41	Chlorfenapyr	Insecticide, Acaricide	15.60	407.6	405.9	247	227	15
						328	247	20
42	Chlorfenson	Insecticide	14.72	303.2	301.9	175	111	10
						111	75	15
43	Chlorflurenol-methyl	Herbicide, Plant growth regulator	13.79	274.7	274.0	217	152	25
						215	152	20
44	Chlornitrofen	Herbicide	17.19	318.5	316.9	317	287	15
						236	173	5
45	Chlorobenzilate	Insecticide	16.14	325.2	324.0	251	139	10
						139	111	10
46	Chloropropylate	Insecticide	16.14	339.2	338.0	251	139	10
						139	111	10
47	Chloroneb	Fungicide	6.94	207.1	205.9	193	53	30
						191	113	15
48	Chlorothalonil	Fungicide	9.79	265.9	263.8	266	231	20
							170	30
49	Chlorpropham	Herbicide, Plant growth regulator	8.25	213.7	213.0	171	127	15
						153	90	25
50	Chlorpyrifos	Insecticide	12.05	350.6	348.9	314	258	20
						199	171	15
51	Chlorpyrifos-methyl	Insecticide	10.80	322.5	320.8	286	271	20
							93	20
52	Chlorthal-dimethyl	Herbicide	12.18	332.0	329.9	299	221	25
						301	223	25

S#	Compound name	Type of pesticide	RT (min.)	Molecular Weight (g/mol)	Extract Mass (g/mol)	Precursor ion (m/z)	Product ion (m/z)	CE (V)
53	Chlorthion	Insecticide	12.47	297.6	296.9	125	47	10
						297	109	10
54	Chlorthiophos	Insecticide	16.52	361.2	359.9	325	269	10
						269	205	10
55	Chlozolinate	Fungicide	13.22	332.1	331.0	331	259	5
						259	188	10
56	Cinmethylin	Herbicide	11.27	274.4	274.1	154	111	5
						169	107	10
57	Clomazone	Herbicide	9.32	239.7	239.0	125	89	15
						204	107	25
58	Coumaphos	Insecticide	22.61	362.8	362.0	210	182	5
						226	163	10
59	Cyanophos	Insecticide	9.53	243.2	243.0	125	79	5
						243	109	10
60	Cyflufenamid	Fungicide	15.67	412.4	412.1	223	203	12
						188	88	40
61	Cyfluthrin	Insecticide	23.99	434.3	433.0	226	206	10
						163	127	5
62	Cyhalofop-butyl	Herbicide	20.98	357.4	357.1	256	120	20
						229	109	15
63	Cyhalothrin, *γ*-	Insecticide	20.93	449.9	449.1	208	181	5
						197	141	10
	Cyhalothrin, *λ*-	Insecticide	21.27	449.9	449.1	208	181	5
						197	141	10

S#	Compound name	Type of pesticide	RT (min.)	Molecular Weight (g/mol)	Extract Mass (g/mol)	Precursor ion (m/z)	Product ion (m/z)	CE (V)
64	Cypermethrin	Insecticide	24.60	416.3	415.0	165	91	10
						163	127	5
65	Cyprazine	Herbicide	10.71	227.7	227.0	170	109	15
						212	109	30
66	Cyprodinil	Fungicide	13.03	225.3	225.1	225	224	10
						224	208	25
67	DDD, *p,p'*-	Metabolite	16.40	320.0	317.9	237	165	25
						235	165	25
	DDE, *p,p'*-	Metabolite	15.06	318.0	315.9	246	176	30
						248	176	30
	DDT, *o,p'*-	Insecticide	16.47	354.5	351.9	237	165	25
						235	165	25
	DDT, *p,p'*-	Insecticide	17.64	354.5	351.9	237	165	25
						235	165	25
68	Deltamethrin	Insecticide	27.20	505.2	502.9	253	93	15
						253	174	27
69	Desmetryn	Herbicide	10.63	213.3	213.1	213	58	20
							198	5
70	Dialifor	Insecticide	21.84	393.8	393.0	357	97	25
						208	89	25
71	Di-Allate	Herbicide	8.63	270.2	269.0	234	150	20
							192	15
72	Diazinon	Insecticide, Acaricide	9.64	304.3	304.1	199	93	15
							135	10

S#	Compound name	Type of pesticide	RT (min.)	Molecular Weight (g/mol)	Extract Mass (g/mol)	Precursor ion (m/z)	Product ion (m/z)	CE (V)
73	Dichlobenil	Herbicide	5.80	172.0	170.9	173	136	10
						171	136	30
74	Dichlofenthion	Insecticide, Nematicide	10.65	315.1	313.9	279	223	10
						223	205	15
75	Dichlofluanid	Fungicide	8.27	333.2	331.9	224	123	24
						123	77	28
76	Dichlormid	Herbicide safener	5.86	208.1	207.0	172	108	5
						166	56	10
77	Diclobutrazol	Fungicide	15.37	328.2	327.0	272	161	10
						270	159	15
78	Diclofop-methyl	Herbicide	18.21	341.2	340.0	340	253	10
						253	162	40
79	Dicloran	Fungicide	9.01	207.0	205.9	160	124	10
						206	176	15
80	Dicofol	Acaricide	12.49	370.5	367.9	139	75	35
							111	15
81	Dicrotophos	Insecticide, Acaricide	8.28	237.2	237.0	127	95	15
						193	127	5
82	Diethatyl-ethyl	Herbicide	14.44	311.8	311.1	188	160	10
							130	40
83	Diethofencarb	Fungicide	12.10	267.3	267.1	267	225	5
						225	168	10
84	Difenoconazole	Fungicide	26.90	406.3	405.0	325	267	15
						323	265	15

S#	Compound name	Type of pesticide	RT (min.)	Molecular Weight (g/mol)	Extract Mass (g/mol)	Precursor ion (m/z)	Product ion (m/z)	CE (V)
85	Diflufenican	Herbicide	18.29	394.3	394.0	266	218	25
							238	15
86	Dimepiperate	Herbicide	13.61	263.4	263.1	145	112	5
							69	15
87	Dimethachlor	Herbicide	10.66	255.7	255.1	134	105	15
						197	148	10
88	Dimethametryn	Herbicide	13.19	255.4	255.1	212	122	5
							94	20
89	Dimethenamid	Herbicide	10.67	275.8	275.0	154	111	10
						230	154	10
90	Dimethipin	Herbicide	9.24	210.3	210.0	124	76	5
						118	58	5
91	Dimethomorph (*E*)	Fungicide	27.99	387.9	387.1	301	165	12
						387	301	12
	Dimethomorph (*Z*)	Fungicide	28.58	387.9	387.1	387	301	12
						301	165	12
92	Dimethylvinphos (*E*)	Insecticide	11.73	331.5	329.9	295	109	20
						297	109	20
	Dimethylvinphos (*Z*)	Insecticide	12.10	331.5	329.9	295	109	20
						297	109	20
93	Diniconazole	Fungicide	16.25	326.2	325.0	268	136	35
							232	15
94	Dinitramine	Herbicide	9.85	322.2	322.0	261	241	10
						261	195	20

S#	Compound name	Type of pesticide	RT (min.)	Molecular Weight (g/mol)	Extract Mass (g/mol)	Precursor ion (m/z)	Product ion (m/z)	CE (V)
95	Dioxathion	Insecticide	9.42	456.5	456.0	270	97	30
						125	97	5
96	Diphenamid	Herbicide	12.69	239.3	239.1	167	165	30
							152	20
97	Diphenylamine	Fungicide	8.02	169.2	169.0	168	167	20
						169	168	15
98	Dithiopyr	Herbicide	11.31	401.4	401.0	354	286	15
							306	5
99	Edifenphos	Fungicide	17.36	310.4	310.0	173	109	10
						201	109	15
100	Endosulfan, *α*-	Insecticide, Acaricide	14.36	406.9	403.8	241	206	15
						205	170	15
	Endosulfan, *β*-	Insecticide, Acaricide	16.20	406.9	403.8	205	170	15
						207	172	15
	Endosulfan, sulfate-	Insecticide, Acaricide	17.43	422.9	419.8	274	237	25
						272	237	20
101	Endrin	Insecticide	15.85	380.9	377.8	263	193	40
							191	35
	δ-keto-Endrin	Metabolite	18.80	380.9	377.8	317	281	10
							245	20
102	EPN	Insecticide, Acaricide	19.29	323.3	323.0	169	141	5
						157	77	30
103	Epoxiconazole	Fungicide	18.57	329.8	329.0	192	111	25
							138	15

S#	Compound name	Type of pesticide	RT (min.)	Molecular Weight (g/mol)	Extract Mass (g/mol)	Precursor ion (m/z)	Product ion (m/z)	CE (V)
104	EPTC	Herbicide	5.88	189.3	189.1	132	90	5
						189	128	5
105	Etaconazole-1	Fungicide	16.35	328.2	327.0	173	109	25
						245	173	20
	Etaconazole-2	Fungicide	16.75	328.2	327.0	173	109	25
						245	173	20
106	Ethalfluralin	Herbicide	8.12	333.3	333.0	276	202	15
						316	276	5
107	Ethion	Insecticide, Acaricide	16.45	384.5	383.9	231	129	25
							175	10
108	Ethofumesate	Herbicide	11.67	286.3	286.0	161	105	10
						207	137	10
109	Ethoprophos	Insecticide, Nematicide	8.06	242.3	242.0	158	114	5
							97	20
110	Ethychlozate	Plant growth regulator	13.29	238.7	238.0	165	138	10
							102	15
111	Etoxazole	Acaricide	19.65	359.4	359.1	300	270	25
						204	176	10
112	Etridiazole	Fungicide	6.55	247.5	245.9	211	140	25
							183	10
113	Fenamidone	Fungicide	19.67	311.4	311.1	268	180	20
						238	237	10
114	Fenarimol	Fungicide	21.40	331.2	330.0	251	139	15
						219	107	10

S#	Compound name	Type of pesticide	RT (min.)	Molecular Weight (g/mol)	Extract Mass (g/mol)	Precursor ion (m/z)	Product ion (m/z)	CE (V)
115	Fenbuconazole	Fungicide	23.57	336.8	336.1	198	129	5
						129	78	20
116	Fenchlorphos	Insecticide	11.24	321.5	319.9	285	270	20
							240	30
117	Fenclorim	Herbicide safener	8.78	225.1	223.9	189	104	10
						224	189	20
118	Fenfuram	Fungicide	9.99	201.2	201.0	201	109	25
						109	53	20
119	Fenitrothion	Insecticide	11.61	277.2	277.0	277	260	5
							109	20
120	Fenobucarb	Insecticide	7.77	207.3	207.1	121	103	15
						150	121	5
121	Fenothiocarb	Acaricide	14.26	253.4	253.1	72	56	10
						160	72	8
122	Fenoxanil	Fungicide	15.84	329.2	328.0	189	125	10
							154	10
123	Fenpropathrin	Insecticide, Acaricide	19.71	349.4	349.1	265	210	10
							89	40
124	Fenpropimorph	Fungicide	12.28	303.5	303.2	128	70	15
							110	10
125	Fenpyrazamine	Fungicide	21.33	331.4	331.1	230	188	10
							117	30
126	Fenson	Insecticide, Acaricide	12.64	268.7	267.9	141	77	10
						268	77	20

S#	Compound name	Type of pesticide	RT (min.)	Molecular Weight (g/mol)	Extract Mass (g/mol)	Precursor ion (m/z)	Product ion (m/z)	CE (V)
127	Fenthion	Insecticide	12.17	278.3	278.0	278	109	20
							169	20
128	Fenvalerate-1	Insecticide, Acaricide	26.06	419.9	419.1	167	125	10
						169	127	10
	Fenvalerate-2	Insecticide, Acaricide	26.47	419.9	419.1	167	125	10
						169	127	10
129	Fipronil	Insecticide	13.10	437.1	435.9	367	213	30
							255	30
130	Flamprop-isopropyl	Herbicide	16.19	363.8	363.1	105	77	15
							51	40
131	Fluacrypyrim	Acaricide	16.80	426.4	426.1	145	102	30
						204	189	5
132	Fluazifop-butyl	Herbicide	15.96	383.4	383.1	282	91	5
							238	25
133	Fluchloralin	Herbicide	9.66	355.7	355.0	306	264	5
						326	63	15
134	Flucythrinate-1	Insecticide	24.74	451.5	451.1	157	107	15
						199	107	25
	Flucythrinate-2	Insecticide	25.13	451.5	451.1	157	107	15
						199	107	25
135	Fluensulfone	Nematicide	7.70	291.7	290.9	119	59	30
							92	10
136	Flufenpyr-ethyl	Herbicide	16.15	408.7	408.0	408	345	15
						321	286	15

S#	Compound name	Type of pesticide	RT (min.)	Molecular Weight (g/mol)	Extract Mass (g/mol)	Precursor ion (m/z)	Product ion (m/z)	CE (V)
137	Flumetralin	Plant growth regulator	14.20	421.7	421.0	143	117	20
							107	30
138	Flumioxazin	Herbicide	25.95	354.3	354.1	354	326	5
						287	259	5
139	Fluopyram	Fungicide	13.35	396.7	396.0	173	95	35
							145	20
140	Fluorochloridone	Herbicide	12.49	312.1	311.0	187	109	20
						311	174	20
141	Fluquinconazole	Fungicide	22.84	376.2	375.0	340	108	40
							286	30
142	Flusilazole	Fungicide	15.26	315.4	315.1	233	165	20
							152	20
143	Flutianil	Fungicide	25.19	426.4	426.0	231	216	5
							200	10
144	Fluvalinate-1	Insecticide, Acaricide	26.36	502.9	502.1	250	55	20
							200	20
	Fluvalinate-2	Insecticide, Acaricide	26.51	502.9	502.1	250	55	20
							200	20
145	Fluxapyroxad	Fungicide	19.27	381.3	380.0	159	43	30
							139	10
146	Fonofos	Insecticide	9.64	246.3	246.0	246	137	5
							109	15
147	Formothion	Insecticide, Acaricide	10.40	257.3	256.9	126	93	5
						170	93	15

S#	Compound name	Type of pesticide	RT (min.)	Molecular Weight (g/mol)	Extract Mass (g/mol)	Precursor ion (m/z)	Product ion (m/z)	CE (V)
148	Fthalide	Fungicide	12.52	271.9	269.8	241	213	15
						243	215	15
149	Halfenprox	Acaricide	24.51	477.3	476.0	265	117	10
						263	129	40
150	Heptachlor	Insecticide	11.22	373.3	369.8	272	237	20
						274	237	20
	Heptachlor epoxide	Metabolite	13.26	389.3	385.8	353	253	30
						217	182	20
151	Heptenophos	Insecticide	7.46	250.6	250.0	124	63	40
							89	15
152	Hexythiazox	Acaricide	13.96	352.9	352.1	184	149	5
						156	155	5
153	Indanofan	Herbicide	19.78	340.8	340.0	139	75	35
						159	103	15
154	Indoxacarb	Insecticide	27.34	527.8	527.0	203	106	25
							134	15
155	Ipconazole	Fungicide	21.28	333.9	333.1	125	99	20
							89	20
156	Iprobenfos	Fungicide	10.28	288.3	288.0	204	91	10
							121	35
157	Iprodione	Nematicide, Fungicide	19.03	330.2	329.0	314	56	20
							245	10
158	Isazofos	Insecticide, Nematicide	9.93	313.7	313.0	257	162	5
						208	166	10

S#	Compound name	Type of pesticide	RT (min.)	Molecular Weight (g/mol)	Extract Mass (g/mol)	Precursor ion (m/z)	Product ion (m/z)	CE (V)
159	Isofenphos	Insecticide	13.28	345.4	345.1	213	121	20
							185	5
160	Isofenphos-methyl	Insecticide	12.85	331.4	331.1	199	93	30
							121	15
161	Isoprocarb	Insecticide	7.17	193.2	193.1	136	121	10
							77	30
162	Isopropalin	Herbicide	12.82	309.4	309.1	238	165	10
						280	238	5
163	Isoprothiolane	Fungicide, Plant growth regulator	14.86	290.4	290.0	189	89	20
							145	10
164	Isopyrazam	Fungicide	21.87	359.4	359.1	359	159	40
						159	139	10
165	Isotianil	Fungicide	17.48	298.1	296.9	180	91	20
						297	180	15
166	Isoxadifen-ethyl	Herbicide safener	17.19	295.3	295.1	222	205	5
							204	20
167	Kresoxim-methyl	Fungicide	15.37	313.4	313.1	131	89	35
						116	89	15
168	Leptophos	Insecticide	20.46	412.1	409.8	377	362	25
						171	77	25
169	Mefenpyr-diethyl	Herbicide safener	18.72	373.3	372.0	299	253	10
						253	190	20
170	Mepanipyrim	Fungicide	14.42	223.3	223.1	222	221	20
							222	10

S#	Compound name	Type of pesticide	RT (min.)	Molecular Weight (g/mol)	Extract Mass (g/mol)	Precursor ion (m/z)	Product ion (m/z)	CE (V)
171	Mepronil	Fungicide	16.89	269.3	269.1	269	91	40
							119	15
172	Metalaxyl	Fungicide	11.17	279.3	279.1	234	146	20
						249	190	5
173	Methidathion	Insecticide, Acaricide	13.90	302.3	301.9	145	58	15
							85	5
174	Methoprotryn	Herbicide	15.47	271.4	271.1	256	170	25
							212	15
175	Methoxychlor	Insecticide	19.56	345.6	344.0	227	141	40
							169	25
176	Methyl trithion	Insecticide	16.09	314.8	313.9	125	47	15
						157	121	35
177	Metolachlor	Herbicide	11.99	283.8	283.1	162	133	15
						238	162	10
178	Metribuzin	Herbicide	10.79	214.3	214.0	198	82	20
							55	30
179	MGK-264	Insecticide synergist	13.11	275.4	275.1	164	67	5
							98	10
180	Molinate	Herbicide	7.30	187.3	187.1	187	126	5
						126	98	5
181	Monolinuron	Herbicide	9.24	214.6	214.0	126	99	15
						214	61	5
182	Myclobutanil	Fungicide	15.18	288.8	288.1	179	125	15
							152	5

S#	Compound name	Type of pesticide	RT (min.)	Molecular Weight (g/mol)	Extract Mass (g/mol)	Precursor ion (m/z)	Product ion (m/z)	CE (V)
183	Nitrothal-isopropyl	Fungicide	12.50	295.3	295.1	194	120	20
						236	194	10
184	Nonachlor-*cis*	Insecticide	16.35	444.2	439.7	407	300	30
						409	300	15
	Nonachlor-*trans*	Insecticide	14.45	444.2	439.7	407	300	15
						409	300	25
185	Nuarimol	Fungicide	17.99	314.7	314.0	139	111	15
						107	52	30
186	Ortho-phenyl phenol	Fungicide	7.10	170.2	170.0	169	115	30
							141	15
187	Oxadiazon	Herbicide	15.12	345.2	344.0	175	112	5
						258	175	15
188	Oxadixyl	Fungicide	16.33	278.3	278.1	163	117	25
							132	5
189	Oxyfluorfen	Herbicide	15.29	361.7	361.0	300	223	20
						361	300	20
190	Paclobutrazol	Plant growth regulator	14.14	293.8	293.1	236	125	10
							167	5
191	Parathion	Insecticide, Acaricide	12.27	291.3	291.0	291	81	40
							109	10
192	Parathion-methyl	Insecticide	10.96	263.2	263.0	263	79	35
							109	15
193	Penconazole	Fungicide	13.18	284.2	283.0	248	157	30
							192	20

S#	Compound name	Type of pesticide	RT (min.)	Molecular Weight (g/mol)	Extract Mass (g/mol)	Precursor ion (m/z)	Product ion (m/z)	CE (V)
194	Pendimethalin	Herbicide	12.99	281.3	281.1	252	162	10
							208	0
195	Penflufen	Fungicide	16.80	317.4	317.1	141	84	15
						274	141	15
196	Pentachlorobenzonitrile	Fungicide	9.43	275.3	272.8	275	179	30
							240	20
197	Penthiopyrad	Fungicide	16.34	359.4	359.1	302	177	20
						177	101	20
198	Pentoxazone	Herbicide	20.47	353.8	353.0	287	70	10
						285	70	15
199	Permethrin-*cis*	Insecticide	22.72	391.3	390.0	183	168	20
							155	10
	Permethrin-*trans*	Insecticide	22.97	391.3	390.0		168	20
							155	10
200	Perthane	Insecticide	15.92	307.3	306.0	223	165	20
							193	20
201	Phenthoate	Insecticide	13.47	320.4	320.0	274	125	15
							121	10
202	Phosphamidon (*E*)	Insecticide, Acaricide	9.52	299.7	299.0	264	127	10
						127	109	15
	Phosphamidon (*Z*)	Insecticide, Acaricide	10.42	299.7	299.0	264	127	10
						127	109	15
203	Phosalone	Insecticide, Acaricide	20.43	367.8	366.9	182	138	5
							102	15

S#	Compound name	Type of pesticide	RT (min.)	Molecular Weight (g/mol)	Extract Mass (g/mol)	Precursor ion (m/z)	Product ion (m/z)	CE (V)
204	Phosmet	Insecticide, Acaricide	19.15	317.3	316.9	160	133	10
							77	30
205	Picoxystrobin	Fungicide	14.41	367.3	367.1	145	102	30
						335	173	10
206	Piperonyl butoxide	Insecticide synergist	18.50	338.4	338.2	176	131	25
							145	10
207	Pirimicarb	Insecticide	10.22	238.3	238.1	166	96	12
						238	166	10
208	Pirimiphos-ethyl	Insecticide	12.69	333.4	333.1	304	168	10
						318	166	10
209	Pirimiphos-methyl	Insecticide, Acaricide	11.55	305.3	305.0	290	125	20
						233	151	5
210	Pretilachlor	Herbicide	14.86	311.9	311.1	162	132	20
						262	202	5
211	Prochloraz	Fungicide	22.97	376.7	375.0	308	70	15
						180	138	10
	2,4,6-trichlorophenol	Metabolite	5.90	376.7	375.0	132	97	15
						196	97	40
212	Procymidone	Fungicide	13.57	284.1	283.0	283	96	5
						285	96	5
213	Prodiamine	Herbicide	11.59	350.3	350.1	275	255	10
						321	279	10
214	Profenofos	Insecticide, Acaricide	14.95	373.6	371.9	337	267	10
						339	269	10

S#	Compound name	Type of pesticide	RT (min.)	Molecular Weight (g/mol)	Extract Mass (g/mol)	Precursor ion (m/z)	Product ion (m/z)	CE (V)
215	Profluralin	Herbicide	9.39	347.3	347.1	318	199	20
							55	10
216	Prohydrojasmon	Plant growth regulator	9.91	254.4	254.1	153	97	5
						184	83	15
217	Prometon	Herbicide	9.09	225.3	225.1	183	168	5
						210	168	10
218	Prometryn	Herbicide	11.26	241.4	241.1	226	184	10
						241	199	10
219	Propachlor	Herbicide	7.82	211.7	211.0	169	93	10
						176	92	20
220	Propanil	Herbicide	10.67	218.1	217.0	161	126	25
						217	161	25
221	Propazine	Herbicide	9.29	229.7	229.1	229	58	15
						214	172	10
222	Propetamphos	Insecticide, Acaricide	9.47	281.3	281.0	138	64	20
						194	166	5
223	Propham	Herbicide, Plant growth regulator	6.57	179.2	179.0	179	93	15
						137	93	8
224	Propiconazole-1	Fungicide	17.42	342.2	341.0	259	69	10
							173	5
	Propiconazole-2	Fungicide	17.62	342.2	341.0		69	10
							173	5
225	Propisochlor	Herbicide	11.10	283.8	283.1	162	120	20
							147	15

S#	Compound name	Type of pesticide	RT (min.)	Molecular Weight (g/mol)	Extract Mass (g/mol)	Precursor ion (m/z)	Product ion (m/z)	CE (V)
226	Propyzamide	Herbicide	9.59	256.1	255.0	173	145	20
							109	35
227	Prothiofos	Insecticide	14.81	345.2	343.9	267	221	20
							239	10
228	Pyracarbolid	Fungicide	12.70	217.3	217.1	125	97	5
							55	15
229	Pyraclofos	Insecticide	22.04	360.8	360.0	194	138	20
							139	10
230	Pyraflufen-ethyl	Herbicide	17.71	413.2	412.0	412	349	10
						349	307	15
231	Pyrazophos	Fungicide	21.52	373.4	373.0	232	204	10
						221	193	10
232	Pyridalyl	Insecticide	25.10	491.1	488.9	204	176	10
							148	20
233	Pyrifenox	Fungicide	14.06	295.2	294.0	187	124	20
						171	100	25
234	Pyriftalid	Herbicide	21.50	318.3	318.0	318	273	20
							274	5
235	Pyrimethanil	Fungicide	9.77	199.3	199.1	198	118	35
							158	20
236	Pyriminobac-methyl (*E*)	Herbicide	17.58	361.4	361.1	302	230	15
							256	20
	Pyriminobac-methyl (*Z*)	Herbicide	16.14	361.4	361.1		230	15
							256	20

S#	Compound name	Type of pesticide	RT (min.)	Molecular Weight (g/mol)	Extract Mass (g/mol)	Precursor ion (m/z)	Product ion (m/z)	CE (V)
237	Quinalphos	Insecticide, Acaricide	13.49	298.3	298.0	146	118	10
							91	30
238	Quinoxyfen	Fungicide	17.43	308.1	306.9	237	208	30
						307	237	20
239	Quintozene	Fungicide	9.35	295.3	292.8	249	214	10
						295	237	20
240	Quizalofop-ethyl	Herbicide	24.68	372.8	372.0	372	299	10
						299	255	15
241	Silafluofen	Insecticide	25.31	408.6	408.1	179	91	10
						286	258	10
242	Simeconazole	Fungicide	10.98	293.4	293.1	121	101	15
							75	35
243	Simetryn	Herbicide	11.08	213.3	213.1	213	170	10
							185	10
244	Spiromesifen	Insecticide, Acaricide	18.75	370.5	370.2	272	209	10
							254	10
245	Spiroxamine-1	Fungicide	10.98	297.5	297.2	100	72	5
	Spiroxamine-2	Fungicide	11.78			198	126	5
246	Sulfotep	Insecticide, Acaricide	8.39	322.3	322.0	202	146	10

S#	Compound name	Type of pesticide	RT (min.)	Molecular Weight (g/mol)	Extract Mass (g/mol)	Precursor ion (m/z)	Product ion (m/z)	CE (V)
						322	146	25
247	Tebuconazole	Fungicide	18.09	307.8	307.1	250	125	30
						252	127	30
248	Tebufenpyrad	Acaricide	19.89	333.9	333.1	276	171	10
						333	171	20
249	Tebupirimfos	Insecticide	10.21	318.4	318.1	276	234	5
						261	137	15
250	Tecnazene	Fungicide, Plant growth regulator	7.70	260.9	258.8	213	142	25
						215	179	25
251	Tefluthrin	Insecticide	9.95	418.7	418.0	177	137	20
							127	20
252	Terbacil	Herbicide	9.89	216.7	216.0	160	76	15
						161	88	20
253	Terbumeton	Herbicide	9.32	225.3	225.1	169	154	10
						210	100	20
254	Terbutryn	Herbicide	11.60	241.4	241.1	185	170	5
						241	185	0
255	Tetrachlorvinphos	Insecticide	14.10	366.0	363.8	329	109	25
						331	109	25
256	Tetraconazole	Fungicide	12.34	372.1	371.0	336	204	40
							218	20
257	Tetradifon	Acaricide	20.21	356.0	353.8	229	201	10
						159	111	20
258	Tetramethrin-1	Insecticide	19.18	331.4	331.1	164	77	25

S#	Compound name	Type of pesticide	RT (min.)	Molecular Weight (g/mol)	Extract Mass (g/mol)	Precursor ion (m/z)	Product ion (m/z)	CE (V)
							107	15
	Tetramethrin-2	Insecticide	19.68	331.4	331.1	164	77	25
							107	15
259	Thifluzamide	Fungicide	15.10	528.1	525.8	194	125	25
							166	10
260	Thiometon	Insecticide, Acaricide	8.89	246.3	245.9	125	47	25
						93	63	5
261	Thionazin	Insecticide, Nematicide	7.76	248.2	248.0	192	96	15
						248	140	10
262	Tolclofos-methyl	Fungicide	11.00	301.1	299.9	265	93	30
							250	15
263	Triadimefon	Fungicide	12.36	293.8	293.0	208	111	35
							181	10
264	Triadimenol	Fungicide	13.59	295.8	295.1	130	65	25
						128	65	20
265	Tri-allate	Herbicide	10.11	304.7	303.0	268	184	10
							226	20
266	Triazophos	Insecticide, Nematicide, Acaricide	16.94	313.3	313.0	161	134	5
							106	15
267	Tridiphane	Herbicide synergist	11.29	320.4	317.8	173	109	30
						187	159	15
268	Trifloxystrobin	Fungicide	17.49	408.4	408.1	116	89	15
						222	130	10
269	Triflumizole	Fungicide	13.66	345.8	345.0	206	179	15

S#	Compound name	Type of pesticide	RT (min.)	Molecular Weight (g/mol)	Extract Mass (g/mol)	Precursor ion (m/z)	Product ion (m/z)	CE (V)
						278	43	15
270	Trifluralin	Herbicide	8.26	335.3	335.1	264	206	5
						306	264	10
271	Vinclozolin	Fungicide	10.89	286.1	284.9	198	145	15
						187	124	20
272	Zoxamide	Fungicide	18.62	336.6	335.0	187	159	20
							123	30

Table S2. Method validation parameter of 272 pesticides in brown rice.

No.	Analyte	LOD	LOQ	R^2	ME.(%)	Repeatability (*n*=5)						Reproducibility (*n*=11)					
						0.01 mg kg^{-1}		0.02 mg kg^{-1}		0.1 mg kg^{-1}		0.01 mg kg^{-1}		0.02 mg kg^{-1}		0.1 mg kg^{-1}	
						Rec. (%)	CV (%)	Rec. (%)	CV (%)	Rec. (%)	CV (%)	Rec. (%)	CV (%)	Rec. (%)	CV (%)	Rec. (%)	CV (%)
1	2,6-DIPN	0.002	0.006	0.9979	140.7	119.4	1.6	104.9	1.7	108.3	2.7	115.7	7.7	145.0	28.0	101.2	15.1
2	Acetochlor	0.001	0.002	0.9985	106.2	113.7	1.4	114.4	1.3	98.6	2.3	96.4	16.1	97.1	12.7	97.2	9.2
	EMA	0.002	0.007	0.9983	79.5	64.5	3.2	105.3	8.9	107.2	1.0	94.3	23.2	97.1	18.1	94.0	9.1
	HEMA	0.001	0.004	1.0000	>500	90.8	2.6	119.6	2.7	103.2	5.0	97.7	48.7	89.5	24.4	88.9	9.0
3	Acrinathrin	0.002	0.005	0.9980	>500	111.3	3.4	118.9	0.7	71.1	3.3	93.8	18.9	96.8	15.1	104.8	20.3
4	Alachlor	0.002	0.006	0.9985	94.7	112.6	1.5	115.0	2.0	101.1	1.1	94.5	19.1	96.3	12.9	98.2	7.1
5	Aldrin	0.001	0.002	0.9990	75.6	90.2	2.6	109.7	9.4	105.2	5.4	90.5	8.1	84.9	6.4	83.9	4.9
	Dieldrin	0.001	0.003	0.9992	55.8	117.4	1.4	118.4	2.6	106.9	3.5	93.2	13.2	90.5	15.8	89.6	15.0
6	Allidochlor	0.001	0.003	0.9975	103.8	67.8	9.8	109.6	12.8	95.2	3.6	95.2	17.2	97.6	12.5	96.3	6.5
7	Ametryn	0.001	0.004	0.9988	187.4	116.3	1.2	114.9	1.0	104.1	0.8	85.3	23.2	94.9	13.0	97.1	8.2
8	Anilofos	0.001	0.004	0.9984	>500	82.1	4.8	103.7	4.8	100.0	4.9	95.0	17.4	91.1	11.0	93.1	6.7
9	Aramite	0.001	0.002	0.9990	208.2	116.2	2.0	109.9	5.6	106.0	1.9	101.9	15.8	103.5	11.7	102.0	10.3
10	Aspon	0.001	0.002	0.9999	27.0	119.2	1.4	118.2	1.2	107.2	1.5	89.3	18.2	93.8	12.9	96.2	6.4
11	Atrazine	0.001	0.004	0.9989	167.8	113.9	1.4	112.2	1.7	102.4	2.2	91.0	16.7	93.3	13.5	98.5	7.5
12	Azaconazole	0.002	0.007	0.9989	6.8	112.5	1.1	113.1	3.2	103.7	1.3	98.0	12.8	97.8	7.3	98.7	4.2
13	Benfluralin	0.002	0.007	0.9964	127.5	116.6	0.6	117.0	4.3	102.5	2.0	89.6	22.7	94.1	11.1	98.3	6.5
14	Benfuresate	0.001	0.002	0.9986	81.3	118.2	0.7	113.6	2.6	103.3	2.0	93.4	15.8	94.8	13.2	98.5	7.2
15	Benodanil	0.002	0.005	0.9988	319.9	119.0	0.6	115.7	3.2	102.9	1.9	93.2	11.6	95.0	11.7	101.1	9.7
16	Benzoylprop-ethyl	0.001	0.002	0.9991	138.0	116.6	1.0	117.3	1.6	108.5	1.1	96.8	13.2	97.3	10.4	100.1	6.0
17	BHC, α-	0.001	0.002	0.9989	69.6	108.0	7.1	107.0	12.5	102.3	1.4	92.7	13.8	93.1	7.2	92.0	1.7
	BHC, β-	0.001	0.003	0.9991	50.5	101.9	4.0	116.7	2.9	108.8	3.3	90.1	21.3	91.8	8.9	92.7	1.1

No.	Analyte	LOD	LOQ	R^2	ME.(%)	Repeatability (*n*=5)						Reproducibility (*n*=11)					
						0.01 mg kg^{-1}		0.02 mg kg^{-1}		0.1 mg kg^{-1}		0.01 mg kg^{-1}		0.02 mg kg^{-1}		0.1 mg kg^{-1}	
						Rec. (%)	CV (%)	Rec. (%)	CV (%)	Rec. (%)	CV (%)	Rec. (%)	CV (%)	Rec. (%)	CV (%)	Rec. (%)	CV (%)
	BHC, *δ*-	0.001	0.002	0.9989	60.7	87.0	5.4	101.1	3.3	82.0	2.0	97.1	8.5	93.9	7.7	92.2	1.6
18	BHC, *γ*-	0.001	0.003	0.9978	53.7	89.8	5.1	97.4	13.9	102.9	2.2	91.5	15.1	82.2	1.6	85.0	7.3
19	Bifenox	0.001	0.003	0.9973	352.8	119.9	1.6	115.9	1.0	86.6	3.3	97.2	21.8	100.8	7.8	101.2	2.0
20	Bifenthrin	0.001	0.002	0.9990	218.7	113.1	1.1	116.4	2.7	108.8	1.2	94.0	14.1	92.4	11.8	94.2	8.3
21	Boscalid	0.001	0.002	0.9987	489.4	117.1	1.0	117.7	3.8	106.2	1.0	95.5	14.3	97.7	12.1	103.3	11.4
22	Bromobutide	0.001	0.003	0.9987	79.5	112.5	2.2	113.9	2.8	103.7	0.3	94.4	21.9	94.4	18.0	98.7	9.0
23	Bromophos-methyl	0.001	0.002	0.9985	178.7	111.7	3.1	118.5	1.3	104.4	2.1	93.0	9.7	91.1	6.1	90.8	1.4
24	Bromophos-ethyl	0.002	0.005	0.9991	157.9	111.5	2.8	118.8	0.9	96.5	2.1	91.4	10.9	91.9	9.0	93.2	2.7
25	Bromopropylate	0.001	0.002	0.9989	399.5	116.6	0.8	118.5	3.3	109.0	1.8	93.8	14.5	94.9	11.5	98.1	9.0
26	Bupirimate	0.002	0.005	0.9992	162.6	117.0	0.9	114.6	4.9	109.9	0.5	91.4	14.6	96.8	10.5	99.3	6.9
27	Buprofezin	0.001	0.003	0.9991	112.0	115.8	1.5	115.1	5.1	108.9	1.0	96.2	15.0	97.7	8.6	99.6	5.9
28	Butachlor	0.001	0.004	0.9991	147.8	113.1	2.0	113.2	2.3	95.4	3.4	89.4	17.8	90.0	11.9	94.1	9.6
29	Butafenacil	0.001	0.002	0.9980	>500	114.0	1.0	119.5	2.5	108.5	2.1	100.0	18.7	101.1	15.3	104.2	15.7
30	Butralin	0.002	0.006	0.9976	189.0	118.4	2.5	118.0	4.0	97.0	2.6	96.7	13.7	91.4	9.6	99.5	8.1
31	Butylate	0.002	0.006	0.9974	113.1	72.0	7.4	117.1	4.8	108.2	7.4	97.2	8.3	90.4	3.6	95.9	7.8
32	Cadusafos	0.001	0.002	0.9986	219.2	108.5	3.9	119.4	3.0	106.2	1.6	92.4	20.2	94.6	12.3	95.2	5.6
33	Carbophenothion	0.001	0.002	0.9988	308.0	117.8	1.2	115.0	2.8	104.5	2.1	91.6	12.6	93.3	11.2	97.5	10.2
34	Carboxin	0.001	0.004	0.9990	212.6	109.0	1.4	107.6	5.0	101.2	1.0	99.0	11.8	96.9	8.2	98.7	7.5
35	Carfentrazone-ethyl	0.001	0.002	0.9988	230.1	118.3	1.6	114.3	4.5	102.9	1.6	126.0	47.9	99.8	36.6	85.3	8.6
36	Chinomethionat	0.001	0.002	0.9989	159.8	69.4	9.3	75.9	6.9	76.5	2.2	57.3	46.6	62.1	49.2	63.7	60.4
37	Chlorbenside	0.001	0.003	0.9989	134.1	108.1	3.8	116.5	1.9	105.0	1.4	83.9	18.3	91.3	9.3	93.6	2.8
38	Chlorbufam	0.001	0.002	0.9982	379.2	115.8	2.2	117.7	2.8	96.9	4.0	92.3	22.9	85.2	5.1	91.4	13.6
39	Chlordane-*cis*	0.001	0.003	0.9989	15.2	107.5	1.2	119.3	1.1	104.8	1.8	93.1	12.9	91.0	9.3	92.3	5.4

No.	Analyte	LOD	LOQ	R^2	ME.(%)	Repeatability (*n*=5)						Reproducibility (*n*=11)					
						0.01 mg kg^{-1}		0.02 mg kg^{-1}		0.1 mg kg^{-1}		0.01 mg kg^{-1}		0.02 mg kg^{-1}		0.1 mg kg^{-1}	
						Rec. (%)	CV (%)	Rec. (%)	CV (%)	Rec. (%)	CV (%)	Rec. (%)	CV (%)	Rec. (%)	CV (%)	Rec. (%)	CV (%)
	Chlordane-*trans*	0.001	0.003	0.9989	63.6	113.4	1.8	118.8	1.3	105.3	0.7	91.7	13.3	90.6	9.7	92.0	5.7
40	Chlorethoxyfos	0.002	0.007	0.9982	61.6	91.7	5.7	118.8	4.3	108.6	2.7	96.6	20.6	98.4	16.9	100.8	13.1
41	Chlorfenapyr	0.001	0.003	0.9988	172.8	114.0	4.5	119.8	5.2	107.1	1.8	98.1	14.1	100.5	7.9	99.0	4.6
42	Chlorfenson	0.001	0.003	0.9990	-76.8	111.3	4.2	117.5	2.0	110.9	4.5	95.6	12.2	98.0	8.3	97.1	5.6
43	Chlorflurenol-methyl	0.001	0.002	0.9991	329.5	117.7	0.5	117.9	3.2	105.9	4.9	95.5	16.8	95.4	13.0	97.7	9.3
44	Chlornitrofen	0.001	0.003	0.9986	221.8	118.6	1.3	115.8	3.6	101.8	2.6	90.6	13.4	93.8	10.1	102.5	6.7
45	Chlorobenzilate	0.002	0.006	0.9993	167.2	112.5	1.3	116.0	3.2	109.4	0.9	96.3	12.8	97.4	9.4	99.2	5.4
46	Chloropropylate	0.002	0.005	0.9993	160.4	81.6	7.8	116.7	4.2	106.8	2.6	96.5	12.9	101.9	6.8	100.6	5.0
47	Chloroneb	0.001	0.002	0.9981	103.5	112.5	1.3	116.0	3.2	109.2	0.6	91.4	19.9	94.9	14.8	97.3	2.7
48	Chlorothalonil	0.002	0.007	0.9962	323.0	N.D.	N.D.	N.D.	N.D.	N.D.	N.D.	62.6	57.6	33.9	69.2	17.2	66.5
49	Chlorpropham	0.001	0.003	0.9983	222.8	109.9	3.4	114.8	1.2	106.8	1.1	96.7	20.9	94.4	12.5	94.8	9.0
50	Chlorpyrifos	0.001	0.002	0.9987	132.7	118.4	0.9	117.7	1.3	100.3	2.3	95.8	12.2	94.3	10.5	94.6	5.0
51	Chlorpyrifos-methyl	0.002	0.007	0.9983	134.4	101.7	3.9	110.0	1.4	95.3	2.0	92.7	15.1	93.6	10.4	93.5	4.3
52	Chlorthal-dimethyl	0.001	0.002	0.9990	83.1	114.0	1.4	119.0	1.4	106.9	1.3	93.4	11.5	95.9	9.4	98.2	3.4
53	Chlorthion	0.001	0.002	0.9979	>500	98.5	4.3	100.9	3.5	84.5	4.3	104.7	4.3	99.7	7.5	99.8	10.2
54	Chlorthiophos	0.001	0.002	0.9989	210.7	115.3	2.8	118.4	3.6	104.8	1.6	94.2	11.8	94.2	9.6	96.4	6.5
55	Chlozolinate	0.001	0.002	0.9990	92.9	114.8	1.8	115.0	2.3	96.4	2.0	83.1	15.0	87.8	11.2	93.7	4.9
56	Cinmethylin	0.002	0.007	0.9948	92.4	109.5	11.4	119.0	5.5	106.0	5.2	88.8	23.2	95.1	12.2	95.9	5.9
57	Clomazone	0.002	0.005	0.9986	120.9	111.0	3.7	118.2	2.2	104.2	2.4	93.3	17.4	96.4	12.8	97.8	6.4
58	Coumaphos	0.001	0.003	0.9975	>500	102.3	9.4	109.2	13.1	88.7	0.6	107.2	10.8	101.1	10.8	98.3	17.1
59	Cyanophos	0.001	0.003	0.9984	185.3	114.1	1.3	120.0	1.5	100.3	2.7	102.4	7.4	96.5	7.7	96.6	5.5
60	Cyflufenamid	0.001	0.004	0.9991	255.6	109.3	0.9	117.7	4.2	111.0	0.5	94.9	19.5	96.3	12.5	99.2	8.3
61	Cyfluthrin	0.001	0.002	0.9987	>500	108.5	5.2	70.2	11.8	71.9	2.5	90.9	24.8	94.0	17.8	100.5	23.4

No.	Analyte	LOD	LOQ	R^2	ME.(%)	Repeatability (n=5)						Reproducibility (n=11)					
						0.01 mg kg^{-1}		0.02 mg kg^{-1}		0.1 mg kg^{-1}		0.01 mg kg^{-1}		0.02 mg kg^{-1}		0.1 mg kg^{-1}	
						Rec. (%)	CV (%)	Rec. (%)	CV (%)	Rec. (%)	CV (%)	Rec. (%)	CV (%)	Rec. (%)	CV (%)	Rec. (%)	CV (%)
62	Cyhalofop-butyl	0.001	0.002	0.9986	439.8	117.1	2.4	118.7	2.8	106.8	2.7	95.9	18.5	97.7	13.8	100.8	12.9
63	Cyhalothrin, *γ*-	0.001	0.003	0.9991	>500	107.9	6.0	84.7	6.1	81.8	2.5	89.1	24.7	101.6	18.6	109.5	23.3
	Cyhalothrin, *λ*-	0.001	0.002	0.9983	>500	111.8	3.0	78.8	6.5	71.6	2.6	98.2	17.2	97.4	18.3	106.8	25.1
64	Cypermethrin	0.001	0.003	0.9985	>500	105.2	6.3	70.5	5.9	71.0	3.1	95.6	18.0	95.3	14.9	104.1	18.4
65	Cyprazine	0.001	0.004	0.9988	73.3	119.4	0.7	117.5	2.1	100.2	2.4	86.9	20.9	91.6	16.2	97.0	8.4
66	Cyprodinil	0.001	0.002	0.9989	137.5	117.6	2.4	116.0	0.7	101.1	2.0	96.6	17.2	93.5	9.8	95.1	5.3
67	DDD, *p,p'*-	0.001	0.002	0.9990	110.8	102.5	2.1	111.6	2.5	108.5	0.6	91.8	15.5	91.2	10.2	92.1	5.8
	DDE, *p,p'*-	0.001	0.004	0.9990	86.6	106.0	1.9	108.8	2.6	104.5	2.0	83.8	16.1	87.3	20.8	84.2	11.2
	DDT, *o,p'*-	0.001	0.003	0.9992	-22.2	115.4	5.9	109.8	2.2	101.1	2.3	110.7	17.7	99.8	12.0	93.4	5.4
	DDT, *p,p'*-	0.001	0.004	0.9982	-9.3	106.5	1.7	104.6	3.2	97.9	1.6	123.2	33.4	104.0	18.3	93.4	10.0
68	Deltamethrin	0.001	0.002	0.9991	>500	105.4	7.3	70.2	6.4	75.5	4.0	83.8	21.0	75.5	25.4	72.7	4.4
69	Desmetryn	0.002	0.005	0.9988	133.4	117.6	0.3	116.6	4.6	102.2	2.8	97.8	15.1	102.1	12.6	102.7	9.6
70	Dialifor	0.001	0.004	0.9980	404.0	115.0	3.0	105.9	5.5	91.4	1.9	105.7	9.9	102.8	10.1	104.5	13.9
71	Di-Allate	0.001	0.004	0.9977	102.3	107.8	8.1	115.3	5.4	103.7	2.4	93.7	16.7	97.9	9.9	94.8	3.6
72	Diazinon	0.001	0.002	0.9983	111.3	115.5	2.9	119.7	0.8	106.7	1.0	96.1	13.8	99.9	9.4	99.1	6.1
73	Dichlobenil	0.002	0.007	0.9981	65.5	95.1	10.6	115.0	5.9	108.1	0.6	97.8	12.5	99.5	10.1	96.8	3.6
74	Dichlofenthion	0.002	0.006	0.9980	116.8	119.5	0.7	117.7	4.2	107.5	1.3	91.7	16.2	94.4	10.7	94.9	4.9
75	Dichlofluanid	0.002	0.005	0.9218	137.0	116.3	7.9	111.2	5.9	81.6	7.3	58.0	74.7	47.7	98.3	77.7	87.2
76	Dichlormid	0.001	0.002	1.0000	100.1	98.3	6.4	116.8	3.8	103.3	4.0	97.6	19.4	99.8	13.7	99.3	7.8
77	Diclobutrazol	0.001	0.003	0.9990	>500	116.2	1.3	111.0	6.1	110.0	0.9	91.7	16.9	94.2	10.4	100.3	11.3
78	Diclofop-methyl	0.001	0.003	0.9990	183.2	116.1	1.4	113.7	3.4	104.1	1.7	92.7	16.0	95.1	11.6	96.7	9.9
79	Dicloran	0.001	0.002	0.9983	177.9	115.5	2.2	117.7	1.6	100.0	2.0	93.0	14.8	96.2	11.0	104.8	2.7
80	Dicofol	0.001	0.002	0.9976	47.3	114.0	1.0	119.3	4.1	103.7	3.1	89.8	23.2	87.5	19.1	92.9	6.9

No.	Analyte	LOD	LOQ	R^2	ME.(%)	Repeatability (n=5)						Reproducibility (n=11)					
						0.01 mg kg^{-1}		0.02 mg kg^{-1}		0.1 mg kg^{-1}		0.01 mg kg^{-1}		0.02 mg kg^{-1}		0.1 mg kg^{-1}	
						Rec. (%)	CV (%)	Rec. (%)	CV (%)	Rec. (%)	CV (%)	Rec. (%)	CV (%)	Rec. (%)	CV (%)	Rec. (%)	CV (%)
81	Dicrotophos	0.002	0.006	0.9984	>500	108.3	2.7	98.6	2.9	73.2	3.2	105.0	11.9	107.3	3.5	95.0	12.8
82	Diethatyl-ethyl	0.001	0.002	0.9993	-16.6	119.0	1.9	112.1	2.9	101.0	4.1	91.3	17.4	94.9	14.3	97.6	10.1
83	Diethofencarb	0.002	0.007	0.9986	464.5	117.7	0.6	118.4	0.8	101.7	5.0	91.5	17.4	94.0	15.5	97.9	10.6
84	Difenoconazole	0.002	0.005	0.9982	>500	106.8	10.1	117.2	8.2	112.1	0.8	99.6	19.2	102.4	14.4	104.8	12.1
85	Diflufenican	0.001	0.002	0.9989	371.3	118.9	2.1	118.9	4.0	107.4	2.1	93.7	16.4	96.2	12.6	97.3	11.2
86	Dimepiperate	0.001	0.002	0.9992	148.1	117.8	0.7	119.2	3.7	106.5	2.4	96.2	16.5	93.2	10.5	96.1	6.8
87	Dimethachlor	0.001	0.003	0.9986	88.6	103.2	2.4	115.6	0.4	94.9	0.9	93.8	15.8	95.6	12.2	99.0	6.0
88	Dimethametryn	0.001	0.002	0.9985	-17.9	119.6	0.7	119.2	1.3	101.5	2.9	85.3	19.7	92.6	12.6	95.4	7.9
89	Dimethenamid	0.002	0.005	0.9986	89.7	110.6	0.8	117.1	1.1	100.3	0.8	95.0	15.5	96.6	10.7	98.1	5.9
90	Dimethipin	0.001	0.002	0.9988	129.5	72.4	12.1	72.4	4.6	74.2	2.4	104.3	3.8	99.7	1.8	96.4	1.4
91	Dimethomorph (*E*)	0.001	0.002	0.9978	>500	118.9	1.3	118.0	0.4	110.3	2.0	96.5	19.3	99.5	17.1	102.4	15.0
	Dimethomorph (*Z*)	0.001	0.003	0.9982	>500	112.5	1.7	118.7	2.7	111.8	1.7	102.4	15.5	104.3	13.0	105.6	12.2
92	Dimethylvinphos (*E*)	0.002	0.005	0.9990	343.8	78.5	2.0	100.7	3.9	71.2	3.6	102.3	8.0	97.8	10.9	95.7	9.3
	Dimethylvinphos (*Z*)	0.001	0.003	0.9988	450.7	68.2	4.1	97.1	2.9	96.6	4.3	96.6	12.4	94.7	13.5	93.9	10.7
93	Diniconazole	0.001	0.004	0.9990	>500	119.8	0.7	113.7	4.7	108.4	1.9	92.8	17.1	95.9	10.3	99.8	10.8
94	Dinitramine	0.001	0.002	0.9972	168.0	119.4	1.8	117.3	1.2	101.8	3.9	97.9	17.5	98.1	9.0	105.1	6.9
95	Dioxathion	0.001	0.002	0.9984	95.2	116.6	1.2	119.7	4.5	101.5	1.5	102.1	12.8	92.9	4.3	92.5	2.2
96	Diphenamid	0.002	0.005	0.9991	91.8	118.6	1.8	118.0	2.3	101.1	0.9	95.1	15.1	96.7	10.7	97.6	7.0
97	Diphenylamine	0.001	0.004	0.9977	160.4	100.5	6.9	118.3	3.2	105.2	1.1	90.0	22.4	93.4	16.9	93.4	6.9
98	Dithiopyr	0.001	0.002	0.9986	85.9	115.6	1.1	119.7	0.4	105.4	1.4	90.0	18.6	94.6	12.6	96.7	5.9
99	Edifenphos	0.002	0.005	0.9991	>500	104.1	11.3	91.0	1.0	82.1	2.1	102.2	20.0	98.7	24.6	104.8	19.6
100	Endosulfan, α-	0.001	0.003	0.9993	-31.0	112.3	0.5	119.3	0.4	105.5	2.5	87.5	26.1	93.2	16.5	96.9	5.2
	Endosulfan, β-	0.001	0.002	0.9991	46.6	108.4	1.3	114.0	3.3	104.4	1.2	89.2	16.6	92.2	11.0	96.3	3.0

No.	Analyte	LOD	LOQ	R^2	ME.(%)	Repeatability (n=5)						Reproducibility (n=11)					
						0.01 mg kg^{-1}		0.02 mg kg^{-1}		0.1 mg kg^{-1}		0.01 mg kg^{-1}		0.02 mg kg^{-1}		0.1 mg kg^{-1}	
						Rec. (%)	CV (%)	Rec. (%)	CV (%)	Rec. (%)	CV (%)	Rec. (%)	CV (%)	Rec. (%)	CV (%)	Rec. (%)	CV (%)
	Endosulfan, sulfate-	0.001	0.002	0.9991	113.7	102.9	1.0	107.0	2.9	90.1	0.7	89.6	18.2	85.5	5.3	86.7	4.7
101	Endrin	0.001	0.003	0.9991	74.9	110.1	2.4	118.5	2.8	93.1	0.2	81.5	23.6	85.4	18.2	92.3	8.3
	δ-keto-Endrin	0.001	0.003	0.9986	40.1	112.7	4.7	107.0	3.9	106.2	1.3	103.1	15.4	91.5	11.1	90.7	5.3
102	EPN	0.001	0.003	0.9982	377.3	118.9	0.4	116.2	4.2	96.4	2.6	96.3	16.4	96.5	10.1	106.3	10.9
103	Epoxiconazole	0.001	0.002	0.9989	194.5	118.3	0.2	119.0	3.3	105.7	0.9	93.1	18.6	95.9	12.9	100.9	9.0
104	EPTC	0.002	0.007	0.9979	71.2	82.6	6.0	110.4	9.4	104.9	3.2	104.8	4.9	105.1	6.6	99.7	7.4
105	Etaconazole-1	0.001	0.002	0.9991	159.8	118.6	6.0	116.6	2.9	104.6	1.7	99.8	18.0	99.6	9.7	100.4	6.5
	Etaconazole-2	0.001	0.002	0.9992	105.0	119.6	1.8	115.6	2.4	106.0	1.0	93.9	16.7	99.6	11.9	99.8	8.0
106	Ethalfluralin	0.001	0.002	0.9969	139.2	110.9	2.3	115.8	8.1	99.6	3.5	101.5	16.2	94.6	11.8	100.4	7.4
107	Ethion	0.001	0.002	0.9988	275.6	118.8	0.9	113.3	4.7	107.1	1.9	99.2	15.7	97.2	10.9	99.7	9.8
108	Ethofumesate	0.001	0.004	0.9990	108.3	119.7	2.0	116.3	0.9	103.1	1.0	94.4	17.1	96.3	12.1	98.1	6.1
109	Ethoprophos	0.001	0.002	0.9978	237.8	104.7	1.4	118.1	6.4	102.1	3.9	92.7	19.5	95.5	11.6	95.0	7.9
110	Ethychlozate	0.001	0.002	0.9992	469.8	118.7	1.1	111.4	6.5	100.0	5.1	90.1	13.7	94.1	12.6	98.0	11.6
111	Etoxazole	0.001	0.003	0.9987	253.1	119.1	1.0	119.0	0.6	108.1	1.3	98.1	10.0	99.7	6.9	99.7	9.3
112	Etridiazole	0.002	0.006	0.9984	69.1	100.6	10.8	117.0	5.3	107.5	8.5	106.2	9.0	98.1	13.8	98.3	6.3
113	Fenamidone	0.001	0.002	0.9987	177.4	118.7	0.4	118.7	1.7	108.2	0.8	93.4	13.3	97.8	10.1	102.0	6.9
114	Fenarimol	0.001	0.002	0.9988	309.2	118.4	0.3	119.0	1.0	103.2	1.3	95.5	11.5	98.8	7.9	102.4	8.6
115	Fenbuconazole	0.002	0.005	0.9988	451.2	116.7	0.2	116.6	1.7	109.8	1.3	95.4	13.6	100.8	9.9	103.7	9.3
116	Fenchlorphos	0.002	0.005	0.9985	144.4	107.3	1.9	116.3	2.3	99.2	1.4	86.3	21.1	92.6	9.0	93.4	4.3
117	Fenclorim	0.001	0.002	0.9987	140.2	103.5	0.5	119.8	6.6	108.4	4.0	94.0	12.6	92.9	11.5	90.7	3.7
118	Fenfuram	0.001	0.002	0.9985	144.8	113.8	1.1	119.7	1.8	100.9	4.5	93.2	16.2	95.1	10.8	94.2	6.7
119	Fenitrothion	0.001	0.003	0.9980	401.1	119.9	1.5	114.8	5.4	95.6	3.5	97.2	12.5	94.2	8.8	96.3	5.7
120	Fenobucarb	0.001	0.003	0.9983	233.0	102.2	16.8	86.4	5.3	70.9	2.8	94.3	14.8	93.0	14.4	94.8	9.0

No.	Analyte	LOD	LOQ	R^2	ME.(%)	Repeatability (*n*=5)						Reproducibility (*n*=11)					
						0.01 mg kg^{-1}		0.02 mg kg^{-1}		0.1 mg kg^{-1}		0.01 mg kg^{-1}		0.02 mg kg^{-1}		0.1 mg kg^{-1}	
						Rec. (%)	CV (%)	Rec. (%)	CV (%)	Rec. (%)	CV (%)	Rec. (%)	CV (%)	Rec. (%)	CV (%)	Rec. (%)	CV (%)
121	Fenothiocarb	0.001	0.002	0.9992	217.3	116.8	1.3	119.6	3.2	103.6	6.0	88.8	20.1	93.9	15.8	96.5	10.6
122	Fenoxanil	0.002	0.007	0.9992	>500	110.3	4.0	72.6	8.5	71.8	4.2	84.9	21.5	87.3	20.1	94.7	12.5
123	Fenpropathrin	0.002	0.006	0.9986	206.4	116.4	1.0	112.7	4.5	101.7	1.7	98.2	10.7	98.4	7.6	102.3	7.4
124	Fenpropimorph	0.001	0.002	0.9990	154.0	117.7	1.2	118.2	2.6	103.2	3.1	89.2	13.1	95.4	9.4	97.2	5.1
125	Fenpyrazamine	0.001	0.003	0.9979	>500	110.9	4.0	87.0	7.9	77.0	2.2	96.7	17.5	96.8	14.6	99.5	15.7
126	Fenson	0.001	0.003	0.9989	70.0	117.6	3.6	116.2	1.9	104.5	2.4	94.5	10.7	96.0	10.1	97.3	5.9
127	Fenthion	0.001	0.002	0.9986	170.5	119.9	1.2	119.3	1.7	101.9	2.1	96.7	12.5	95.3	10.1	96.0	5.8
128	Fenvalerate-1	0.001	0.002	0.9984	>500	109.2	3.6	70.6	2.4	107.9	3.7	100.7	18.8	101.8	12.5	107.7	16.3
	Fenvalerate-2	0.001	0.002	0.9981	>500	113.2	4.8	71.3	5.6	107.5	3.5	96.7	19.8	99.2	13.6	108.8	17.5
129	Fipronil	0.001	0.002	0.9984	434.0	118.4	1.9	118.0	1.7	99.8	6.3	95.9	21.7	94.7	16.0	99.0	14.0
130	Flamprop-isopropyl	0.001	0.002	0.9993	118.3	118.1	2.3	118.9	2.9	108.9	0.6	98.9	15.7	101.9	7.6	100.6	5.2
131	Fluacrypyrim	0.001	0.002	0.9991	267.6	119.1	0.4	118.8	3.1	110.7	1.1	96.0	14.1	96.7	12.6	100.8	8.2
132	Fluazifop-butyl	0.001	0.002	0.9991	265.3	119.8	0.4	116.5	4.0	108.6	1.2	96.7	14.1	99.9	9.3	99.9	7.6
133	Fluchloralin	0.001	0.002	0.9967	125.3	119.8	2.0	109.8	7.7	93.2	3.1	97.2	19.3	95.5	13.9	99.7	6.2
134	Flucythrinate-1	0.001	0.003	0.9985	>500	111.9	4.3	83.0	8.2	82.8	3.8	89.5	24.6	101.6	12.9	91.4	17.8
	Flucythrinate-2	0.001	0.002	0.9982	>500	103.4	4.0	81.3	8.7	73.2	2.9	95.3	19.4	100.2	14.5	91.8	18.5
135	Fluensulfone	0.001	0.002	0.9980	189.9	103.9	3.9	109.9	2.0	103.4	4.0	82.8	28.2	91.4	17.6	94.7	10.3
136	Flufenpyr-ethyl	0.001	0.002	0.9991	328.6	101.4	5.1	81.5	7.8	84.6	0.6	97.4	16.4	100.0	8.4	100.5	8.2
137	Flumetralin	0.001	0.003	0.9985	165.6	119.4	2.4	119.5	2.5	96.5	4.9	99.8	21.9	98.3	13.6	101.4	7.7
138	Flumioxazin	0.002	0.007	1.0000	>500	118.3	2.3	108.0	2.9	100.0	1.6	99.6	14.8	94.7	15.7	92.6	8.4
139	Fluopyram	0.001	0.002	0.9990	273.0	117.1	2.6	119.9	0.6	105.2	5.8	94.0	15.8	94.4	16.1	98.3	4.9
140	Fluorochloridone	0.001	0.004	1.0000	138.8	110.3	3.1	117.1	3.6	104.7	0.6	97.1	15.5	93.1	14.6	99.1	12.4
141	Fluquinconazole	0.002	0.006	1.0000	226.8	118.0	3.4	117.7	2.4	93.0	2.4	98.5	10.8	96.8	8.8	100.2	5.7

No.	Analyte	LOD	LOQ	R^2	ME.(%)	Repeatability (n=5)						Reproducibility (n=11)					
						0.01 mg kg^{-1}		0.02 mg kg^{-1}		0.1 mg kg^{-1}		0.01 mg kg^{-1}		0.02 mg kg^{-1}		0.1 mg kg^{-1}	
						Rec. (%)	CV (%)	Rec. (%)	CV (%)	Rec. (%)	CV (%)	Rec. (%)	CV (%)	Rec. (%)	CV (%)	Rec. (%)	CV (%)
142	Flusilazole	0.001	0.003	0.9992	154.8	118.4	1.2	115.1	4.8	109.2	0.9	94.8	14.0	97.5	9.4	99.0	6.1
143	Flutianil	0.001	0.002	0.9983	369.0	118.9	2.1	118.8	3.4	108.5	2.2	98.8	14.3	100.4	11.6	103.5	11.9
144	Fluvalinate-1	0.001	0.003	1.0000	>500	95.7	11.7	113.1	5.7	74.9	0.9	97.4	27.0	93.7	14.8	100.6	17.6
	Fluvalinate-2	0.001	0.003	0.9979	>500	91.5	7.2	115.2	2.9	75.3	3.3	93.3	27.3	91.5	11.3	99.8	16.6
145	Fluxapyroxad	0.002	0.007	0.9989	>500	119.8	2.3	116.1	2.9	109.3	1.2	94.3	10.4	95.8	10.5	99.7	8.8
146	Fonofos	0.001	0.002	0.9976	108.6	116.2	0.1	118.9	1.6	105.4	3.0	98.0	14.4	96.9	11.2	97.0	4.4
147	Formothion	0.002	0.005	0.9983	>500	71.2	7.4	91.3	3.3	87.7	4.5	85.5	13.5	78.4	9.1	81.6	7.0
148	Fthalide	0.001	0.002	0.9994	95.3	95.3	1.0	117.8	3.2	93.3	4.1	93.8	10.4	95.1	8.5	96.8	4.9
149	Halfenprox	0.001	0.002	0.9980	337.3	119.4	2.0	117.7	0.9	101.7	1.6	94.7	15.5	93.7	13.3	106.7	24.7
150	Heptachlor	0.002	0.007	0.9992	63.9	106.3	3.3	117.3	3.4	103.9	4.2	86.9	22.2	87.5	14.2	92.0	4.9
	Heptachlor epoxide	0.001	0.002	0.9991	49.4	111.9	6.5	119.2	3.0	106.2	2.9	97.3	14.6	96.2	6.4	95.0	3.5
151	Heptenophos	0.002	0.005	0.9981	247.0	73.0	9.9	114.2	1.7	83.8	2.6	96.1	14.0	93.7	15.0	94.3	8.9
152	Hexythiazox	0.001	0.003	0.9977	404.5	117.8	3.9	118.5	5.5	111.7	6.5	82.7	18.8	88.8	14.4	93.5	6.8
153	Indanofan	0.001	0.002	0.9989	330.3	117.8	1.8	118.6	0.9	108.3	1.1	95.3	10.2	94.5	4.1	100.2	7.7
154	Indoxacarb	0.002	0.005	0.9993	351.0	83.8	6.9	116.3	3.8	71.6	2.6	78.6	15.0	78.2	20.2	86.6	17.0
155	Ipconazole	0.002	0.005	0.9981	>500	119.5	2.6	117.4	0.7	108.7	1.6	99.1	18.3	101.0	11.9	104.7	12.0
156	Iprobenfos	0.001	0.004	0.9983	438.4	119.9	2.2	118.6	0.7	107.7	3.7	98.8	17.1	97.5	13.4	99.5	8.4
157	Iprodione	0.001	0.002	0.9994	>500	100.5	14.2	89.0	7.3	91.8	3.0	100.1	11.5	98.0	12.9	97.7	21.6
158	Isazofos	0.001	0.004	0.9976	100.5	116.7	1.7	120.0	2.9	104.9	2.6	97.2	13.1	99.1	10.5	99.1	5.1
159	Isofenphos	0.002	0.005	0.9991	139.1	117.0	1.6	116.0	2.2	104.6	2.7	92.9	16.7	95.1	12.6	97.6	7.9
160	Isofenphos-methyl	0.002	0.006	0.9989	143.2	119.1	2.4	117.8	3.0	104.2	2.6	94.0	15.0	95.6	11.8	98.1	6.5
161	Isoprocarb	0.001	0.002	0.9985	258.3	74.0	3.4	89.4	5.3	71.1	0.9	96.7	23.2	95.5	18.4	93.3	13.3
162	Isopropalin	0.002	0.006	0.9975	167.8	114.8	5.5	118.5	3.5	99.0	2.4	91.1	14.5	91.2	10.5	96.8	7.1

No.	Analyte	LOD	LOQ	R^2	ME.(%)	Repeatability (*n*=5)						Reproducibility (*n*=11)					
						0.01 mg kg^{-1}		0.02 mg kg^{-1}		0.1 mg kg^{-1}		0.01 mg kg^{-1}		0.02 mg kg^{-1}		0.1 mg kg^{-1}	
						Rec. (%)	CV (%)	Rec. (%)	CV (%)	Rec. (%)	CV (%)	Rec. (%)	CV (%)	Rec. (%)	CV (%)	Rec. (%)	CV (%)
163	Isoprothiolane	0.001	0.003	0.9942	-59.4	114.0	6.5	108.1	5.6	107.6	1.5	89.6	17.1	90.8	17.5	93.0	13.5
164	Isopyrazam	0.001	0.003	0.9985	>500	108.7	3.3	118.3	2.8	106.9	2.6	93.7	14.1	98.9	11.3	102.4	11.9
165	Isotianil	0.001	0.002	0.9979	>500	95.8	2.7	102.0	6.5	101.0	0.9	104.6	7.2	98.4	4.4	99.7	11.5
166	Isoxadifen-ethyl	0.001	0.002	0.9989	196.3	119.2	0.5	111.9	3.7	103.6	1.1	98.3	12.4	97.2	10.5	101.7	12.0
167	Kresoxim-methyl	0.001	0.003	0.9993	101.3	116.3	1.9	116.9	4.2	109.1	1.3	95.2	11.7	99.2	8.2	101.0	5.3
168	Leptophos	0.001	0.002	0.9988	257.8	98.6	2.4	97.9	5.0	91.3	1.0	88.1	5.3	82.1	9.0	89.3	4.1
169	Mefenpyr-diethyl	0.001	0.002	0.9989	255.0	114.1	0.6	119.2	3.2	108.6	1.4	98.6	11.8	98.8	10.5	101.0	8.7
170	Mepanipyrim	0.001	0.003	0.9984	-11.6	116.0	1.8	115.7	3.0	104.3	6.3	92.7	22.3	91.4	14.5	93.9	8.6
171	Mepronil	0.001	0.002	0.9990	>500	119.8	0.7	117.9	4.5	106.4	1.7	95.7	12.1	98.9	8.3	99.9	7.8
172	Metalaxyl	0.001	0.002	0.9988	92.0	117.8	2.8	119.6	1.1	100.7	1.7	90.9	18.5	92.6	14.8	97.5	7.2
173	Methidathion	0.001	0.002	1.0000	415.7	105.3	2.2	109.6	3.3	86.2	3.1	102.1	8.1	100.3	9.5	93.6	10.7
174	Methoprotryn	0.002	0.006	0.9989	237.4	118.3	0.1	113.1	5.2	105.8	0.9	97.0	16.3	97.6	9.4	97.0	9.1
175	Methoxychlor	0.001	0.004	0.9974	64.3	118.1	1.5	117.2	3.3	104.5	1.5	112.0	5.7	104.5	3.9	92.2	7.3
176	Methyl trithion	0.001	0.003	0.9985	338.4	110.1	2.0	108.8	2.9	96.4	2.7	92.6	14.5	93.0	7.2	91.5	6.5
177	Metolachlor	0.001	0.003	0.9989	132.4	117.9	0.6	117.9	4.2	102.2	1.6	95.1	16.3	94.7	13.2	96.5	7.6
178	Metribuzin	0.002	0.005	0.9988	123.1	115.3	2.2	117.0	3.5	98.8	1.0	99.9	15.0	100.4	11.9	100.8	8.6
179	MGK-264	0.001	0.002	0.9988	116.8	116.9	0.9	116.5	2.6	102.9	1.8	93.9	15.8	93.4	8.1	98.8	9.5
180	Molinate	0.001	0.004	0.9984	94.1	103.1	7.8	115.8	4.8	104.7	6.1	220.9	105.3	152.0	72.1	97.5	6.3
181	Monolinuron	0.002	0.006	0.9953	137.7	94.0	5.0	115.1	3.6	98.2	3.9	91.9	21.0	102.3	9.1	103.2	5.8
182	Myclobutanil	0.002	0.006	0.9992	159.0	119.1	0.5	114.2	4.7	107.4	0.8	97.0	13.3	97.1	8.5	98.9	4.9
183	Nitrothal-isopropyl	0.001	0.002	0.9973	233.4	117.7	4.5	116.7	4.0	94.7	3.2	97.1	18.1	95.0	9.9	104.9	9.2
184	Nonachlor-*cis*	0.001	0.004	0.9991	56.2	108.0	4.1	114.4	2.5	101.8	0.4	88.3	15.5	85.6	13.8	93.1	2.3
	Nonachlor-*trans*	0.001	0.003	0.9988	-28.7	109.3	3.6	114.8	2.1	103.8	2.5	93.3	18.6	92.9	8.2	93.4	3.3

No.	Analyte	LOD	LOQ	R^2	ME.(%)	Repeatability (*n*=5)						Reproducibility (*n*=11)					
						0.01 mg kg^{-1}		0.02 mg kg^{-1}		0.1 mg kg^{-1}		0.01 mg kg^{-1}		0.02 mg kg^{-1}		0.1 mg kg^{-1}	
						Rec. (%)	CV (%)	Rec. (%)	CV (%)	Rec. (%)	CV (%)	Rec. (%)	CV (%)	Rec. (%)	CV (%)	Rec. (%)	CV (%)
185	Nuarimol	0.001	0.002	0.9992	252.5	117.7	1.4	117.0	2.6	103.7	0.8	93.6	17.7	92.7	13.4	96.5	8.8
186	Ortho-phenyl phenol	0.001	0.004	0.9980	369.6	104.5	7.3	115.8	1.5	103.5	2.9	96.0	35.1	95.7	21.5	97.3	6.9
187	Oxadiazon	0.001	0.003	0.9989	133.3	117.3	2.5	117.2	4.6	108.9	1.0	94.6	14.1	97.0	9.0	96.7	6.6
188	Oxadixyl	0.002	0.006	0.9990	110.9	99.0	2.0	103.2	5.0	87.6	1.6	97.6	12.6	97.7	8.1	100.6	6.4
189	Oxyfluorfen	0.001	0.002	0.9982	333.9	117.2	3.7	115.9	5.7	107.0	3.5	97.1	19.9	93.7	13.2	98.9	2.1
190	Paclobutrazol	0.001	0.003	0.9992	>500	118.1	1.7	119.0	1.3	102.7	8.2	91.1	21.5	94.1	18.1	96.2	14.1
191	Parathion	0.001	0.002	0.9979	278.8	113.1	4.5	118.6	1.9	98.4	4.4	95.3	15.5	100.2	9.8	104.3	9.3
192	Parathion-methyl	0.001	0.002	0.9978	269.0	119.0	2.4	119.6	1.7	93.1	3.3	101.6	8.7	96.4	5.4	97.9	6.6
193	Penconazole	0.001	0.002	0.9991	120.9	116.9	1.0	118.9	0.4	102.7	3.0	92.4	15.9	93.6	11.9	97.9	6.8
194	Pendimethalin	0.001	0.002	0.9971	240.6	117.2	4.3	114.9	3.4	98.3	3.3	95.2	13.8	91.8	9.6	99.3	8.6
195	Penflufen	0.001	0.002	0.9992	320.6	117.5	0.8	115.9	3.0	106.3	1.4	95.7	15.7	98.0	9.9	99.5	7.8
196	Pentachlorobenzonitrile	0.001	0.002	0.9989	111.7	97.9	5.7	112.8	3.8	106.4	3.8	93.2	13.3	94.0	10.3	93.7	3.6
197	Penthiopyrad	0.001	0.002	0.9989	450.0	119.5	0.5	115.7	4.2	107.3	1.2	95.8	14.4	96.9	10.7	99.9	8.1
198	Pentoxazone	0.001	0.002	0.9989	218.4	118.1	1.4	118.8	3.2	106.7	1.3	94.2	12.2	96.6	9.7	100.1	7.0
199	Permethrin-cis	0.002	0.007	0.9987	410.2	84.9	13.0	114.6	1.6	98.7	1.8	94.6	21.3	96.1	14.7	99.2	13.9
	Permethrin-trans	0.001	0.002	0.9984	358.0	115.0	4.5	109.8	3.8	106.0	1.8	99.8	18.0	97.5	13.0	98.8	14.2
200	Perthane	0.001	0.002	0.9989	123.2	116.5	1.0	115.5	3.4	107.5	0.8	92.7	15.0	94.4	11.7	95.6	7.9
201	Phenthoate	0.001	0.002	0.9988	193.4	118.7	2.0	119.4	1.8	102.0	3.4	99.3	14.2	97.3	13.3	100.3	11.7
202	Phosphamidon (*E*)	0.002	0.005	0.9987	>500	82.1	9.7	91.7	8.6	109.7	7.4	92.9	26.5	102.5	15.2	89.4	21.5
	Phosphamidon (*Z*)	0.002	0.005	0.9983	>500	107.5	11.0	96.1	2.1	96.5	6.5	87.1	29.9	102.7	14.7	91.9	21.1
203	Phosalone	0.001	0.003	0.9980	>500	106.8	2.8	95.1	5.8	83.6	1.5	105.9	5.7	96.2	7.9	95.8	9.6
204	Phosmet	0.001	0.004	0.9977	>500	83.3	5.7	87.6	5.1	102.4	6.4	84.3	13.3	88.7	23.8	93.7	24.7
205	Picoxystrobin	0.001	0.003	0.9987	29.0	118.6	3.5	119.4	3.3	108.5	5.7	92.8	16.3	95.6	12.6	97.5	8.0

No.	Analyte	LOD	LOQ	R^2	ME.(%)	Repeatability (*n*=5)						Reproducibility (*n*=11)					
						0.01 mg kg^{-1}		0.02 mg kg^{-1}		0.1 mg kg^{-1}		0.01 mg kg^{-1}		0.02 mg kg^{-1}		0.1 mg kg^{-1}	
						Rec. (%)	CV (%)	Rec. (%)	CV (%)	Rec. (%)	CV (%)	Rec. (%)	CV (%)	Rec. (%)	CV (%)	Rec. (%)	CV (%)
206	Piperonyl butoxide	0.001	0.002	0.9989	447.4	119.4	1.4	117.7	4.0	107.8	2.0	99.1	9.6	99.3	10.8	101.0	9.0
207	Pirimicarb	0.001	0.004	0.9985	144.5	111.4	2.3	116.7	4.6	100.5	2.0	92.3	18.7	95.3	13.1	96.9	7.8
208	Pirimiphos-ethyl	0.001	0.002	0.9987	154.3	115.9	2.2	119.1	2.9	103.8	2.6	94.4	14.5	96.2	11.8	98.3	7.6
209	Pirimiphos-methyl	0.001	0.002	0.9985	191.4	118.2	1.1	116.6	4.7	101.0	3.9	92.8	16.2	91.3	15.3	96.0	6.5
210	Pretilachlor	0.001	0.002	0.9932	-62.6	99.7	6.1	99.1	6.1	92.9	0.9	90.7	18.7	92.3	16.2	93.1	11.8
211	Prochloraz	0.001	0.004	0.9980	>500	118.1	11.7	117.9	6.9	103.8	1.6	92.7	11.8	101.2	10.6	102.6	16.4
	2,4,6-trichlorophenol	0.002	0.006	0.9954	222.2	85.3	4.1	111.3	5.6	98.5	4.1	71.2	15.5	67.9	15.3	69.2	22.6
212	Procymidone	0.001	0.002	0.9991	88.7	118.1	0.7	118.6	1.2	105.6	1.6	93.5	12.7	95.0	11.6	97.4	5.8
213	Prodiamine	0.001	0.002	0.9981	208.6	119.4	1.9	118.4	2.4	100.8	4.1	91.3	15.9	95.7	8.1	101.4	6.8
214	Profenofos	0.001	0.002	0.9991	>500	65.9	6.1	70.7	7.1	108.8	7.5	89.6	23.3	89.8	20.4	88.8	17.2
215	Profluralin	0.001	0.002	0.9976	128.3	117.5	1.3	115.9	6.3	101.7	3.6	101.5	15.1	103.8	12.4	103.1	6.4
216	Prohydrojasmon	0.001	0.004	0.9975	162.1	119.6	2.4	118.5	2.4	101.9	4.7	84.9	28.4	88.4	16.7	100.2	9.0
217	Prometon	0.001	0.003	0.9989	170.7	115.7	2.6	118.5	4.2	104.5	2.5	91.7	16.3	97.5	11.4	102.6	10.5
218	Prometryn	0.002	0.006	0.9990	121.4	116.2	0.3	118.1	0.8	102.3	3.2	87.3	18.6	93.4	12.5	97.3	7.4
219	Propachlor	0.002	0.005	0.9984	127.4	86.7	6.1	113.7	3.1	89.6	0.7	90.8	21.0	95.8	14.5	95.3	9.1
220	Propanil	0.001	0.002	0.9984	192.5	112.9	3.4	99.3	1.9	100.1	4.8	84.6	27.6	93.4	16.4	98.2	6.4
221	Propazine	0.002	0.006	0.9989	121.6	113.5	0.1	118.2	0.6	105.4	1.1	91.4	14.3	92.9	13.6	97.3	4.3
222	Propetamphos	0.001	0.002	0.9980	178.0	113.8	3.1	116.8	1.9	105.0	3.0	96.3	16.3	96.4	12.2	98.5	7.4
223	Propham	0.001	0.004	0.9978	206.9	98.4	9.6	119.7	2.5	105.2	1.6	95.0	18.0	96.0	13.7	96.5	6.3
224	Propiconazole-1	0.001	0.003	0.9990	223.4	117.4	0.7	118.6	3.3	107.3	0.6	93.3	16.1	98.7	10.3	100.9	8.5
	Propiconazole-2	0.001	0.003	0.9993	219.9	117.3	1.1	118.5	1.1	104.5	1.1	95.4	7.7	99.0	6.9	100.5	5.3
225	Propisochlor	0.001	0.002	0.9990	95.8	117.5	1.8	114.7	4.5	100.8	1.1	94.3	18.1	97.8	10.7	99.4	7.8
226	Propyzamide	0.002	0.006	0.9984	150.2	114.8	3.2	119.7	0.6	103.1	2.4	101.3	16.5	96.6	11.5	97.1	6.4

No.	Analyte	LOD	LOQ	R^2	ME.(%)	Repeatability (n=5)						Reproducibility (n=11)					
						0.01 mg kg^{-1}		0.02 mg kg^{-1}		0.1 mg kg^{-1}		0.01 mg kg^{-1}		0.02 mg kg^{-1}		0.1 mg kg^{-1}	
						Rec. (%)	CV (%)	Rec. (%)	CV (%)	Rec. (%)	CV (%)	Rec. (%)	CV (%)	Rec. (%)	CV (%)	Rec. (%)	CV (%)
227	Prothiofos	0.001	0.002	0.9989	-63.2	101.6	7.4	104.3	4.5	104.2	2.6	84.9	19.5	87.2	15.5	86.5	13.2
228	Pyracarbolid	0.001	0.003	0.9969	274.7	91.9	6.6	119.5	1.8	96.9	5.2	85.6	16.5	93.7	12.6	96.5	10.6
229	Pyraclofos	0.002	0.006	0.9979	>500	71.1	9.1	82.1	7.9	104.1	6.0	107.1	13.8	104.3	16.7	96.6	6.9
230	Pyraflufen-ethyl	0.001	0.003	0.9989	297.3	118.9	0.9	114.6	3.5	104.2	2.0	86.3	21.9	91.8	17.8	96.9	12.4
231	Pyrazophos	0.001	0.002	0.9978	>500	117.3	2.1	113.0	4.7	95.9	2.7	105.6	13.0	100.9	14.2	102.1	15.8
232	Pyridalyl	0.001	0.002	0.9982	>500	118.3	1.4	116.4	3.1	102.8	2.7	91.3	18.1	92.7	14.8	97.1	15.5
233	Pyrifenox	0.002	0.005	0.9992	154.3	117.4	0.3	116.9	3.7	104.3	3.9	90.8	17.6	94.8	13.5	96.5	9.2
234	Pyriftalid	0.002	0.005	0.9985	397.2	106.1	0.6	117.4	2.4	103.2	1.7	100.0	10.8	102.7	9.7	108.1	10.3
235	Pyrimethanil	0.002	0.006	0.9985	148.8	117.4	1.8	119.7	0.2	102.7	4.7	91.6	17.1	93.3	11.5	96.7	4.9
236	Pyriminobac-methyl (*E*)	0.001	0.002	0.9991	262.9	110.3	0.8	117.4	3.6	108.0	1.3	98.0	17.9	98.6	13.2	100.5	11.7
	Pyriminobac-methyl (*Z*)	0.001	0.004	0.9990	150.4	115.3	1.9	115.8	3.9	108.6	0.7	97.3	12.1	99.7	9.0	101.5	7.3
237	Quinalphos	0.002	0.006	0.9989	163.8	117.2	1.8	76.6	0.2	105.2	3.2	91.9	9.7	98.8	11.8	98.2	9.0
238	Quinoxyfen	0.001	0.003	0.9992	119.3	110.9	1.9	114.8	3.4	104.9	1.3	90.1	14.1	92.2	10.2	94.0	6.3
239	Quintozene	0.001	0.002	0.9980	135.8	104.4	8.3	117.0	4.8	97.1	5.3	87.2	7.1	87.0	4.0	85.8	15.4
240	Quizalofop-ethyl	0.001	0.002	0.9983	>500	110.0	0.7	118.1	0.9	106.4	2.0	88.4	19.5	90.8	9.7	94.0	8.3
241	Silafluofen	0.001	0.002	0.9981	306.5	117.3	0.8	119.2	2.5	102.8	1.7	91.2	13.8	89.5	11.5	92.6	7.4
242	Simeconazole	0.001	0.003	0.9988	198.5	118.4	3.1	119.0	3.2	105.1	3.8	94.4	18.8	93.1	16.2	98.3	8.8
243	Simetryn	0.001	0.003	0.9990	140.0	114.1	1.0	114.9	1.9	100.2	1.8	88.0	20.1	91.9	15.1	95.1	9.5
244	Spiromesifen	0.001	0.003	0.9992	261.1	93.8	3.7	86.9	6.7	83.5	1.6	92.2	14.3	92.9	11.9	97.6	8.2
245	Spiroxamine-1	0.001	0.002	0.9989	194.0	105.9	1.6	119.4	8.4	99.5	5.1	76.3	26.6	92.7	16.7	91.2	3.9
	Spiroxamine-2	0.001	0.003	0.9986	183.4	106.4	2.2	112.0	1.8	95.7	4.7	82.8	32.8	96.6	12.5	93.2	2.6
246	Sulfotep	0.001	0.003	0.9981	110.3	113.5	4.3	117.9	1.0	104.7	1.2	97.1	16.0	97.2	12.4	97.1	7.2
247	Tebuconazole	0.002	0.007	0.9990	458.4	118.3	0.9	115.1	3.8	105.0	1.0	93.8	18.1	93.0	13.4	96.8	10.9

No.	Analyte	LOD	LOQ	R^2	ME.(%)	Repeatability (*n*=5)						Reproducibility (*n*=11)					
						0.01 mg kg^{-1}		0.02 mg kg^{-1}		0.1 mg kg^{-1}		0.01 mg kg^{-1}		0.02 mg kg^{-1}		0.1 mg kg^{-1}	
						Rec. (%)	CV (%)	Rec. (%)	CV (%)	Rec. (%)	CV (%)	Rec. (%)	CV (%)	Rec. (%)	CV (%)	Rec. (%)	CV (%)
248	Tebufenpyrad	0.002	0.006	0.9987	260.7	117.8	1.1	117.2	3.2	107.7	1.8	94.8	13.0	97.0	9.2	98.9	8.1
249	Tebupirimfos	0.001	0.002	0.9983	126.3	112.0	3.1	118.6	2.4	104.5	2.5	89.8	18.9	95.2	11.7	96.5	7.5
250	Tecnazene	0.002	0.005	0.9979	120.5	105.9	6.3	113.8	1.7	104.6	6.8	95.2	12.4	96.2	9.0	94.1	3.4
251	Tefluthrin	0.001	0.002	0.9981	86.1	112.8	2.0	119.1	1.3	109.2	1.3	93.2	14.9	94.6	11.2	94.5	4.1
252	Terbacil	0.001	0.002	0.9983	372.1	119.6	0.7	119.7	0.4	95.0	8.3	96.1	10.3	93.0	16.1	93.7	11.9
253	Terbumeton	0.001	0.003	0.9990	150.4	117.6	2.2	119.8	0.2	106.0	2.6	89.6	19.9	92.6	15.3	98.0	4.9
254	Terbutryn	0.001	0.003	0.9991	144.8	113.1	2.5	116.4	3.7	103.4	2.2	92.5	15.1	92.5	13.4	96.3	5.3
255	Tetrachlorvinphos	0.002	0.006	0.9992	450.8	119.9	0.1	78.2	2.6	101.4	0.7	88.3	23.5	98.9	15.9	98.0	8.1
256	Tetraconazole	0.002	0.005	0.9991	135.4	118.2	0.8	118.3	2.6	105.6	3.2	93.5	12.8	95.6	10.5	98.7	6.1
257	Tetradifon	0.001	0.003	0.9989	103.2	118.0	1.1	119.2	2.5	104.1	1.3	97.6	7.6	96.1	7.6	98.7	5.1
258	Tetramethrin-1	0.001	0.003	0.9983	>500	75.2	4.2	110.0	13.7	96.3	1.6	97.6	20.9	99.3	12.1	99.2	12.3
	Tetramethrin-2	0.001	0.003	0.9988	494.1	88.5	13.1	114.3	2.1	107.6	1.5	132.8	14.4	103.6	5.1	91.2	10.1
259	Thifluzamide	0.001	0.002	0.9991	>500	115.6	2.3	108.8	7.5	109.0	1.7	92.1	16.4	97.1	10.2	98.2	9.0
260	Thiometon	0.001	0.003	0.9984	157.8	110.9	6.0	115.6	4.5	100.7	9.3	91.3	19.4	98.2	16.5	98.3	15.6
261	Thionazin	0.001	0.003	0.9980	128.8	104.8	6.2	117.4	7.4	104.4	1.9	96.2	16.9	95.6	13.6	95.1	7.9
262	Tolclofos-methyl	0.001	0.003	0.9988	106.1	114.4	1.3	119.4	1.5	105.2	0.5	94.0	15.5	93.9	11.8	95.9	3.6
263	Triadimefon	0.001	0.003	0.9989	126.9	118.8	1.0	119.6	0.7	104.4	2.2	98.4	12.6	102.4	10.4	99.9	4.9
264	Triadimenol	0.002	0.005	0.9991	293.2	117.8	2.5	117.3	2.4	107.0	5.1	87.6	9.5	85.5	16.3	95.0	11.5
265	Tri-allate	0.002	0.006	0.9988	96.8	109.3	3.7	115.9	2.4	109.1	2.1	93.9	11.1	94.4	10.6	93.5	3.7
266	Triazophos	0.001	0.002	0.9990	457.6	110.2	2.8	100.4	6.9	92.9	2.1	101.8	6.2	90.7	19.5	101.3	11.1
267	Tridiphane	0.001	0.004	0.9984	41.7	113.5	3.1	118.5	1.3	103.7	2.1	105.5	5.5	99.5	6.5	97.3	8.1
268	Trifloxystrobin	0.001	0.002	0.9993	204.5	118.8	0.5	117.2	3.2	109.6	1.3	93.8	15.2	101.2	10.5	102.5	8.9
269	Triflumizole	0.002	0.007	0.9990	159.6	116.7	2.6	71.4	0.3	102.9	3.5	90.5	20.9	96.0	13.0	96.8	7.3

No.	Analyte	LOD	LOQ	R^2	ME.(%)	Repeatability (*n*=5)						Reproducibility (*n*=11)					
						0.01 mg kg^{-1}		0.02 mg kg^{-1}		0.1 mg kg^{-1}		0.01 mg kg^{-1}		0.02 mg kg^{-1}		0.1 mg kg^{-1}	
						Rec. (%)	CV (%)	Rec. (%)	CV (%)	Rec. (%)	CV (%)	Rec. (%)	CV (%)	Rec. (%)	CV (%)	Rec. (%)	CV (%)
270	Trifluralin	0.001	0.002	0.9964	131.8	110.9	0.9	115.3	5.0	104.1	1.8	97.3	18.6	98.8	13.5	100.4	9.6
271	Vinclozolin	0.001	0.002	0.9982	83.6	117.4	2.2	117.5	1.4	104.6	1.7	92.3	15.2	94.5	11.4	96.4	7.5
272	Zoxamide	0.001	0.004	0.9989	>500	93.8	16.7	79.9	6.2	77.2	3.3	104.6	18.1	94.2	18.9	92.0	13.8

Table S3. Method validation parameter of 272 pesticides in soybean.

No.	Analyte	LOD	LOQ	R^2	ME.(%)	Repeatability (*n*=5)						Reproducibility (*n*=11)					
						0.01 mg kg^{-1}		0.02 mg kg^{-1}		0.1 mg kg^{-1}		0.01 mg kg^{-1}		0.02 mg kg^{-1}		0.1 mg kg^{-1}	
						Rec. (%)	CV (%)	Rec. (%)	CV (%)	Rec. (%)	CV (%)	Rec. (%)	CV (%)	Rec. (%)	CV (%)	Rec. (%)	CV (%)
1	2,6-DIPN	0.003	0.008	0.9967	72.2	76.9	7.7	71.3	9.4	61.0	9.5	74.8	24.1	84.6	9.0	61.0	9.1
2	Acetochlor	0.001	0.003	0.9996	43.8	68.8	10.5	80.6	5.3	88.0	11.9	97.8	19.3	98.8	8.2	95.4	8.5
	EMA	0.001	0.003	0.9985	60.7	41.5	22.9	64.7	16.9	80.3	18.7	81.4	26.1	86.2	16.3	89.1	9.2
	HEMA	0.002	0.005	0.9996	>500	46.1	12.2	62.2	9.6	85.6	16.0	97.2	65.8	50.5	59.6	62.7	37.2
3	Acrinathrin	0.001	0.004	0.9994	50.3	75.6	8.7	92.7	6.2	96.7	2.2	85.4	57.8	87.5	45.0	102.7	39.4
4	Alachlor	0.001	0.004	0.9994	38.0	70.6	8.7	83.9	4.8	82.8	1.9	86.2	23.9	91.3	12.9	93.0	11.1
5	Aldrin	0.001	0.002	0.9994	35.4	42.8	9.6	55.2	5.2	55.6	2.2	35.8	28.0	43.6	37.6	46.3	28.3
	Dieldrin	0.001	0.003	0.9970	23.3	63.3	8.6	76.5	4.3	74.2	2.6	56.0	18.4	62.9	30.5	67.0	15.1
6	Allidochlor	0.001	0.004	0.9994	75.8	76.8	11.0	95.8	5.5	91.9	6.5	96.8	11.0	100.6	5.9	97.8	3.5
7	Ametryn	0.001	0.002	0.9999	90.2	88.3	12.3	72.1	7.4	86.7	7.1	80.6	29.9	86.7	14.5	88.7	12.7
8	Anilofos	0.001	0.004	0.9989	157.2	98.8	8.3	118.2	4.3	118.4	3.3	52.0	38.1	61.5	38.7	62.8	25.2
9	Aramite	0.001	0.004	0.9999	83.5	68.0	9.7	84.3	4.1	89.9	8.8	86.4	31.8	96.4	9.0	93.4	21.8
10	Aspon	0.001	0.003	0.9962	70.0	61.5	8.5	74.4	3.3	84.3	16.1	82.8	18.3	83.1	15.0	84.6	12.2
11	Atrazine	0.002	0.005	0.9997	95.9	67.3	10.2	80.2	5.0	88.4	11.2	87.1	20.7	90.1	12.9	91.9	7.4
12	Azaconazole	0.002	0.005	0.9999	51.7	67.6	9.0	83.3	4.5	89.4	9.9	90.9	19.7	92.1	13.2	95.0	9.9
13	Benfluralin	0.001	0.002	0.9985	90.5	77.2	7.3	84.2	3.0	86.7	14.5	95.3	17.6	88.8	14.4	88.9	15.5
14	Benfuresate	0.001	0.004	0.9991	32.0	68.8	8.1	81.8	5.3	88.7	11.3	95.6	17.9	93.0	9.8	92.2	9.6
15	Benodanil	0.001	0.004	0.9995	127.8	81.9	7.3	94.8	4.8	94.9	5.2	99.4	18.0	101.8	9.7	106.6	9.6
16	Benzoylprop-ethyl	0.001	0.004	0.9998	64.9	65.4	10.5	80.8	4.1	87.9	11.6	88.6	25.5	91.3	18.7	93.7	16.0
17	BHC, *α*-	0.001	0.002	0.9968	7.8	69.3	11.5	83.4	4.4	85.7	14.6	82.8	31.7	92.0	10.9	85.0	16.2
	BHC, *β*-	0.001	0.003	1.0000	35.8	78.2	6.2	90.2	3.3	91.7	9.0	89.2	23.7	95.5	9.8	89.4	13.4

No.	Analyte	LOD	LOQ	R^2	ME.(%)	Repeatability (*n*=5)						Reproducibility (*n*=11)					
						0.01 mg kg^{-1}		0.02 mg kg^{-1}		0.1 mg kg^{-1}		0.01 mg kg^{-1}		0.02 mg kg^{-1}		0.1 mg kg^{-1}	
						Rec. (%)	CV (%)	Rec. (%)	CV (%)	Rec. (%)	CV (%)	Rec. (%)	CV (%)	Rec. (%)	CV (%)	Rec. (%)	CV (%)
	BHC, *δ*-	0.002	0.005	0.9986	-59.4	80.7	8.6	87.9	8.5	86.6	19.2	91.7	20.5	89.8	19.2	84.4	15.0
18	BHC, *γ*-	0.001	0.003	0.9991	6.3	85.4	9.2	99.5	4.9	90.9	4.0	59.0	5.6	64.3	24.9	64.3	29.5
19	Bifenox	0.001	0.003	0.9989	130.4	117.4	16.9	75.5	10.3	90.3	8.2	123.9	33.4	127.7	50.3	133.0	51.7
20	Bifenthrin	0.001	0.004	0.9999	120.1	102.3	8.9	113.0	9.3	116.2	3.8	91.2	29.6	77.3	28.1	77.9	28.0
21	Boscalid	0.001	0.004	0.9999	281.5	70.8	9.0	86.8	4.9	92.3	7.2	91.4	21.1	95.0	19.7	97.5	13.8
22	Bromobutide	0.001	0.003	0.9951	32.6	77.4	8.8	87.3	5.4	91.1	8.8	91.5	17.5	90.1	20.0	93.3	13.8
23	Bromophos-methyl	0.001	0.004	0.9982	55.9	66.0	8.5	77.5	3.2	84.1	16.7	79.0	24.2	84.8	11.1	88.4	6.6
24	Bromophos-ethyl	0.001	0.003	0.9982	59.9	88.1	15.9	96.1	4.0	108.0	5.3	79.9	22.6	83.3	14.2	82.6	14.3
25	Bromopropylate	0.001	0.003	0.9993	189.9	63.3	8.9	75.8	3.5	83.9	17.0	84.0	36.2	88.9	21.2	88.8	26.0
26	Bupirimate	0.001	0.003	1.0000	79.7	66.7	9.7	83.2	3.2	88.3	11.2	96.4	20.8	89.5	19.2	93.8	12.8
27	Buprofezin	0.001	0.002	0.9998	50.2	60.6	9.7	74.2	4.6	83.4	17.5	80.8	27.4	89.0	11.6	84.4	19.0
28	Butachlor	0.001	0.003	0.9999	72.8	67.7	11.7	79.7	5.5	87.5	13.0	85.2	25.0	91.9	9.7	92.2	16.2
29	Butafenacil	0.001	0.003	0.9987	249.4	76.3	8.6	90.2	5.0	91.5	8.2	96.6	18.4	96.7	21.9	97.7	20.1
30	Butralin	0.001	0.003	0.9993	74.6	75.7	5.8	77.5	3.3	85.0	17.4	88.8	10.6	86.2	17.1	88.8	24.2
31	Butylate	0.001	0.003	0.9999	70.5	52.9	19.6	79.7	5.8	79.2	5.2	59.7	19.8	66.0	21.5	68.4	15.6
32	Cadusafos	0.001	0.004	0.9994	140.1	64.5	11.5	79.5	4.3	85.5	14.1	83.6	25.7	96.7	7.5	88.6	13.0
33	Carbophenothion	0.001	0.004	0.9999	117.8	66.5	7.0	76.9	2.9	84.8	15.6	85.6	29.9	87.4	15.9	89.9	19.4
34	Carboxin	0.001	0.004	0.9999	100.5	61.2	10.6	77.0	5.5	87.8	12.0	91.1	26.3	92.6	16.0	96.5	16.6
35	Carfentrazone-ethyl	0.001	0.003	0.9993	87.8	83.7	6.7	98.4	4.5	97.5	2.6	86.2	21.1	94.2	39.3	97.8	31.9
36	Chinomethionat	0.001	0.004	0.9997	-23.1	71.9	7.0	83.7	7.9	88.8	12.4	60.4	27.9	62.0	44.6	58.5	57.5
37	Chlorbenside	0.001	0.003	0.9999	58.8	49.6	8.3	62.6	4.2	64.3	1.9	42.5	23.8	53.5	24.1	59.2	12.4
38	Chlorbufam	0.002	0.005	0.9980	126.3	77.9	10.4	95.1	7.1	95.8	3.1	86.7	4.5	87.4	18.3	90.2	16.4
39	Chlordane-*cis*	0.001	0.003	0.9962	37.4	112.2	9.8	96.9	2.1	107.7	6.4	75.1	25.8	74.2	24.0	81.5	15.5

No.	Analyte	LOD	LOQ	R^2	ME.(%)	Repeatability (*n*=5)						Reproducibility (*n*=11)					
						0.01 mg kg^{-1}		0.02 mg kg^{-1}		0.1 mg kg^{-1}		0.01 mg kg^{-1}		0.02 mg kg^{-1}		0.1 mg kg^{-1}	
						Rec. (%)	CV (%)	Rec. (%)	CV (%)	Rec. (%)	CV (%)	Rec. (%)	CV (%)	Rec. (%)	CV (%)	Rec. (%)	CV (%)
	Chlordane-*trans*	0.001	0.002	0.9968	25.9	109.9	7.4	95.2	3.0	107.6	5.2	74.2	26.8	73.5	24.9	80.6	15.8
40	Chlorethoxyfos	0.001	0.002	0.9982	55.7	62.3	11.6	84.5	2.7	87.3	9.6	84.2	24.2	88.6	14.4	89.6	16.0
41	Chlorfenapyr	0.001	0.004	0.9997	66.4	70.1	10.7	85.8	4.3	89.5	9.6	88.9	16.3	90.7	11.5	90.4	16.7
42	Chlorfenson	0.001	0.003	0.9989	55.7	63.5	9.6	81.9	4.3	88.3	11.2	93.0	16.5	89.7	14.6	89.6	13.4
43	Chlorflurenol-methyl	0.001	0.004	0.9999	141.5	68.7	8.5	84.1	4.7	90.1	9.7	94.6	21.2	93.4	15.4	96.6	14.5
44	Chlornitrofen	0.001	0.004	0.9995	93.7	101.3	5.9	107.0	3.6	99.3	2.9	98.3	24.9	98.0	23.3	88.2	22.5
45	Chlorobenzilate	0.001	0.004	0.9998	83.4	65.9	10.0	80.7	3.9	86.0	14.0	87.0	28.0	94.0	11.4	88.1	21.0
46	Chloropropylate	0.001	0.003	0.9998	79.9	65.9	10.0	80.7	3.9	86.0	14.0	84.8	31.3	94.1	11.3	92.3	14.4
47	Chloroneb	0.001	0.003	0.9993	72.6	64.6	13.6	80.8	5.1	85.0	14.0	77.5	25.5	87.0	15.5	88.0	5.6
48	Chlorothalonil	0.002	0.007	0.9934	-63.1	74.7	14.3	69.3	10.1	71.8	44.0	49.8	84.7	61.4	26.1	95.4	94.0
49	Chlorpropham	0.001	0.003	0.9995	160.1	62.6	11.3	79.6	4.3	86.3	13.1	86.2	22.6	88.3	12.3	91.8	9.7
50	Chlorpyrifos	0.001	0.003	0.9988	44.0	60.1	8.7	72.7	3.5	83.1	17.8	78.8	26.6	86.2	12.6	84.4	15.6
51	Chlorpyrifos-methyl	0.001	0.004	0.9994	62.9	71.8	7.8	84.1	4.5	88.3	11.9	87.8	17.5	89.3	12.1	87.4	12.2
52	Chlorthal-dimethyl	0.001	0.003	0.9959	32.7	62.2	10.3	76.5	3.5	85.1	15.1	80.1	26.4	90.7	9.1	85.3	16.2
53	Chlorthion	0.001	0.003	0.9973	172.4	93.3	5.7	102.3	3.6	97.9	4.1	101.2	19.8	94.5	21.4	97.9	19.6
54	Chlorthiophos	0.001	0.003	0.9999	73.0	63.6	9.4	76.9	3.6	84.7	15.4	80.6	28.5	86.8	14.4	87.7	16.0
55	Chlozolinate	0.001	0.004	0.9994	25.5	64.9	11.3	82.0	5.5	89.6	9.6	83.6	28.9	89.9	17.0	92.4	15.4
56	Cinmethylin	0.002	0.005	0.9968	76.2	103.6	13.8	119.7	13.7	119.0	3.8	90.5	28.6	86.7	21.2	82.5	15.0
57	Clomazone	0.001	0.003	0.9997	81.3	68.2	10.1	87.7	4.6	90.7	7.6	88.6	14.1	96.0	4.0	96.8	2.1
58	Coumaphos	0.001	0.003	0.9989	244.8	116.5	24.0	106.3	14.1	107.7	7.3	104.0	7.7	83.4	20.0	105.3	21.0
59	Cyanophos	0.001	0.004	0.9997	81.3	80.6	8.5	94.2	4.2	94.6	4.5	92.5	4.5	94.0	11.9	95.9	8.8
60	Cyflufenamid	0.001	0.003	0.9999	113.2	73.8	9.3	87.2	3.1	89.5	10.2	90.0	16.1	97.8	4.3	96.0	17.7
61	Cyfluthrin	0.001	0.003	0.9997	188.7	81.5	8.0	98.1	4.7	97.9	1.6	90.4	32.1	95.2	30.7	104.1	35.3

No.	Analyte	LOD	LOQ	R^2	ME.(%)	Repeatability (*n*=5)						Reproducibility (*n*=11)					
						0.01 mg kg^{-1}		0.02 mg kg^{-1}		0.1 mg kg^{-1}		0.01 mg kg^{-1}		0.02 mg kg^{-1}		0.1 mg kg^{-1}	
						Rec. (%)	CV (%)	Rec. (%)	CV (%)	Rec. (%)	CV (%)	Rec. (%)	CV (%)	Rec. (%)	CV (%)	Rec. (%)	CV (%)
62	Cyhalofop-butyl	0.001	0.003	0.9995	200.1	71.4	7.1	84.2	3.5	88.8	11.1	89.5	23.0	91.2	22.0	91.9	16.7
63	Cyhalothrin, *γ*-	0.001	0.004	1.0000	113.4	69.7	7.9	86.9	3.9	92.2	6.9	74.0	32.1	78.8	32.7	83.7	34.4
	Cyhalothrin, *λ*-	0.001	0.004	0.9995	220.1	78.1	8.5	92.5	4.1	94.8	4.4	74.9	38.5	79.4	38.6	84.5	39.2
64	Cypermethrin	0.001	0.003	0.9998	160.4	82.9	7.7	98.0	4.6	97.3	2.6	88.6	35.0	92.9	33.2	104.3	38.1
65	Cyprazine	0.001	0.004	0.9985	49.6	62.9	9.7	77.2	5.3	87.5	12.0	82.8	21.5	89.2	10.9	90.9	13.2
66	Cyprodinil	0.001	0.003	0.9990	59.4	103.5	11.5	71.2	6.6	83.8	6.8	76.7	27.0	80.4	18.2	82.2	18.3
67	DDD, *p,p'*-	0.001	0.003	0.9999	13.0	61.3	7.2	73.0	4.2	82.2	19.1	71.9	44.5	76.6	35.3	78.2	33.3
	DDE, *p,p'*-	0.001	0.002	0.9985	52.5	91.2	8.2	108.4	4.5	106.7	1.2	54.0	49.0	60.4	41.1	62.8	34.1
	DDT, *o,p'*-	0.001	0.003	0.9986	3.1	72.8	16.1	67.6	14.0	80.2	32.8	71.8	34.8	71.3	30.6	67.4	21.9
	DDT, *p,p'*-	0.001	0.004	0.9991	-0.9	71.3	21.3	74.2	19.0	85.1	32.9	74.6	36.5	75.1	34.5	73.4	39.0
68	Deltamethrin	0.002	0.005	0.9999	446.9	106.8	1.7	96.3	7.1	105.2	4.5	84.8	31.7	99.4	21.9	94.2	24.6
69	Desmetryn	0.001	0.003	0.9985	62.9	63.2	10.1	75.7	4.5	86.1	14.4	89.8	19.1	91.9	7.3	93.4	8.4
70	Dialifor	0.001	0.003	0.9988	52.2	90.8	8.5	101.6	5.3	98.1	1.5	84.6	28.6	86.9	17.6	97.6	24.1
71	Di-Allate	0.001	0.002	0.9998	74.0	113.6	10.8	74.2	11.2	86.0	9.4	73.7	27.0	86.6	8.0	85.6	11.4
72	Diazinon	0.001	0.003	0.9995	63.1	68.0	9.4	79.7	4.0	86.0	14.3	83.9	15.4	86.1	14.4	87.6	16.5
73	Dichlobenil	0.001	0.002	0.9994	48.7	63.0	13.4	81.2	5.3	86.0	13.1	84.4	15.9	88.5	9.9	88.6	7.4
74	Dichlofenthion	0.001	0.003	0.9988	43.1	69.2	9.3	81.0	3.0	79.5	1.8	57.8	50.1	62.9	46.3	64.7	36.0
75	Dichlofluanid	0.001	0.004	0.9482	28.1	101.5	8.7	92.3	7.6	86.7	18.0	82.6	87.6	80.4	89.5	92.1	106.6
76	Dichlormid	0.001	0.003	0.9992	73.7	80.8	8.8	99.3	5.1	93.4	5.1	99.2	8.5	102.6	6.5	101.9	2.7
77	Diclobutrazol	0.002	0.005	0.9999	309.4	65.3	10.9	81.6	5.3	88.3	11.1	92.2	20.7	92.8	15.4	89.5	7.7
78	Diclofop-methyl	0.001	0.003	0.9998	106.8	67.9	8.3	79.9	3.6	86.5	13.6	85.3	20.8	93.1	10.1	95.0	12.8
79	Dicloran	0.001	0.003	0.9991	91.6	121.1	2.3	133.9	4.0	130.2	2.7	57.3	24.6	63.8	34.3	96.7	14.1
80	Dicofol	0.001	0.003	0.9995	40.4	79.7	11.8	75.2	5.2	76.7	5.1	76.6	21.0	77.0	14.7	82.9	13.8

No.	Analyte	LOD	LOQ	R^2	ME.(%)	Repeatability (*n*=5)						Reproducibility (*n*=11)					
						0.01 mg kg^{-1}		0.02 mg kg^{-1}		0.1 mg kg^{-1}		0.01 mg kg^{-1}		0.02 mg kg^{-1}		0.1 mg kg^{-1}	
						Rec. (%)	CV (%)	Rec. (%)	CV (%)	Rec. (%)	CV (%)	Rec. (%)	CV (%)	Rec. (%)	CV (%)	Rec. (%)	CV (%)
81	Dicrotophos	0.002	0.006	0.9966	421.9	86.7	11.6	106.8	9.0	101.2	5.3	106.3	8.6	104.1	12.5	103.4	12.3
82	Diethatyl-ethyl	0.001	0.004	0.9985	86.6	78.2	8.9	93.6	3.9	92.0	7.0	98.5	10.5	101.5	2.8	100.3	10.8
83	Diethofencarb	0.001	0.004	0.9994	196.6	119.0	8.6	74.0	9.9	88.9	7.5	89.0	24.8	88.5	18.9	94.6	17.2
84	Difenoconazole	0.002	0.006	0.9992	315.5	77.3	7.9	90.5	3.8	91.6	8.3	96.5	20.2	97.8	22.3	98.3	19.7
85	Diflufenican	0.001	0.004	0.9999	187.0	67.7	8.5	79.5	3.6	86.6	13.7	87.7	24.5	88.9	21.2	93.5	22.2
86	Dimepiperate	0.001	0.004	0.9989	67.3	118.2	10.3	72.1	15.4	81.8	6.6	80.5	22.9	89.0	10.4	85.9	20.4
87	Dimethachlor	0.001	0.003	0.9951	38.5	75.3	8.7	89.9	5.2	93.0	6.4	92.9	10.9	95.4	9.2	96.1	10.1
88	Dimethametryn	0.001	0.004	0.9998	60.2	106.6	11.6	72.0	9.2	86.0	6.9	96.0	15.6	83.2	19.3	86.3	19.2
89	Dimethenamid	0.001	0.003	0.9996	56.3	70.5	8.8	84.0	4.8	90.0	9.6	90.9	13.6	90.5	11.9	93.6	11.7
90	Dimethipin	0.003	0.008	0.9997	-29.2	86.4	15.0	99.8	14.0	102.5	9.6	105.5	13.4	105.0	14.8	106.2	11.3
91	Dimethomorph (*E*)	0.002	0.005	0.9998	454.6	80.3	7.0	92.8	5.3	94.9	4.9	101.5	20.3	99.4	21.5	106.6	24.9
	Dimethomorph (*Z*)	0.001	0.004	0.9999	441.5	81.1	6.4	93.5	5.0	94.8	5.0	103.6	18.4	101.2	18.7	108.0	23.1
92	Dimethylvinphos (*E*)	0.002	0.005	0.9991	152.9	88.1	7.4	102.5	5.2	99.0	2.6	97.2	11.8	97.4	20.9	100.5	16.4
	Dimethylvinphos (*Z*)	0.001	0.004	0.9992	233.8	83.0	8.6	98.0	5.3	98.5	3.7	96.6	10.6	97.8	15.1	99.9	16.4
93	Diniconazole	0.001	0.004	0.9989	281.4	64.4	9.2	79.0	3.8	87.0	12.6	90.4	24.7	90.2	20.9	87.8	9.7
94	Dinitramine	0.001	0.003	0.9996	74.7	79.5	8.5	85.5	3.0	89.4	11.1	87.3	19.3	94.4	1.0	93.7	20.1
95	Dioxathion	0.001	0.003	0.9999	56.4	74.4	8.9	85.6	5.8	88.9	9.8	86.6	5.9	92.4	2.8	90.6	14.1
96	Diphenamid	0.001	0.003	0.9996	39.7	91.7	2.9	83.1	8.7	94.0	6.9	87.9	22.8	89.5	11.9	93.6	10.6
97	Diphenylamine	0.001	0.002	0.9993	95.9	95.3	15.7	70.7	2.7	82.1	17.2	72.5	29.9	82.4	12.1	82.0	14.5
98	Dithiopyr	0.001	0.003	0.9996	51.1	68.4	8.4	80.0	2.5	86.3	14.0	88.0	15.4	87.3	17.0	88.6	17.2
99	Edifenphos	0.002	0.006	0.9990	252.6	87.0	8.7	105.4	5.8	102.0	4.5	105.3	12.1	101.2	23.9	77.0	23.9
100	Endosulfan, *α*-	0.001	0.002	0.9998	46.3	60.5	8.9	71.3	3.1	116.5	6.5	88.8	21.3	86.5	9.8	90.6	10.8
	Endosulfan, *β*-	0.001	0.004	0.9995	16.0	69.3	7.0	81.3	3.5	85.8	14.0	86.6	17.5	88.2	13.5	86.5	30.2

No.	Analyte	LOD	LOQ	R^2	ME.(%)	Repeatability (*n*=5)						Reproducibility (*n*=11)					
						0.01 mg kg^{-1}		0.02 mg kg^{-1}		0.1 mg kg^{-1}		0.01 mg kg^{-1}		0.02 mg kg^{-1}		0.1 mg kg^{-1}	
						Rec. (%)	CV (%)	Rec. (%)	CV (%)	Rec. (%)	CV (%)	Rec. (%)	CV (%)	Rec. (%)	CV (%)	Rec. (%)	CV (%)
	Endosulfan, sulfate-	0.001	0.003	0.9999	27.3	104.1	10.6	99.8	2.8	112.4	5.2	85.3	33.7	85.5	23.2	86.6	30.0
101	Endrin	0.001	0.003	0.9989	40.4	80.9	12.8	72.5	1.4	78.1	5.5	84.3	24.3	83.6	12.0	86.5	15.0
	δ-keto-Endrin	0.001	0.004	0.9996	24.3	77.8	6.6	75.3	8.3	78.5	5.5	90.4	9.9	87.3	5.5	92.3	7.2
102	EPN	0.001	0.002	0.9993	146.4	100.4	6.3	107.9	4.4	101.2	1.6	104.0	11.7	100.5	22.1	96.9	21.0
103	Epoxiconazole	0.001	0.004	0.9997	105.1	74.1	7.6	87.4	3.7	92.2	7.1	96.6	14.1	95.9	10.7	96.3	7.9
104	EPTC	0.001	0.003	0.9996	43.9	62.3	15.5	79.0	5.7	83.3	16.2	88.1	18.5	91.0	3.9	90.4	9.3
105	Etaconazole-1	0.001	0.004	0.9993	74.2	64.3	11.7	82.1	3.0	88.4	10.3	94.3	21.1	99.3	7.8	97.3	7.6
	Etaconazole-2	0.002	0.005	0.9993	48.4	62.8	11.3	81.2	5.6	89.5	9.9	88.0	29.4	95.2	7.4	98.9	9.5
106	Ethalfluralin	0.001	0.003	0.9991	89.7	76.9	8.0	85.1	3.3	87.1	12.9	87.4	32.5	89.8	9.2	93.9	11.9
107	Ethion	0.001	0.003	0.9996	88.3	72.3	7.7	85.8	3.3	89.1	10.1	81.7	23.4	92.7	6.2	93.5	23.8
108	Ethofumesate	0.001	0.003	0.9996	43.9	64.4	10.4	78.0	5.2	87.5	12.5	88.4	19.4	90.5	11.8	91.9	12.8
109	Ethoprophos	0.001	0.004	0.9992	151.1	67.0	11.1	83.0	4.6	88.3	10.5	92.4	16.9	90.6	13.1	92.6	9.0
110	Ethychlozate	0.002	0.006	0.9997	161.2	61.3	5.8	74.6	5.6	88.3	12.0	89.0	24.4	91.4	15.4	99.3	17.5
111	Etoxazole	0.001	0.004	0.9994	115.8	65.9	7.6	76.4	3.2	84.8	15.7	80.2	29.7	93.3	10.3	89.3	23.6
112	Etridiazole	0.001	0.002	0.9971	77.8	98.7	16.0	97.0	14.5	87.2	16.7	80.5	18.8	88.9	11.6	88.6	14.3
113	Fenamidone	0.001	0.004	0.9992	91.2	67.1	12.0	79.8	5.8	88.3	11.8	86.8	23.7	96.4	9.0	97.7	16.7
114	Fenarimol	0.001	0.004	0.9986	143.4	68.3	8.4	80.8	4.1	87.9	12.5	89.9	22.5	93.2	17.4	96.5	21.5
115	Fenbuconazole	0.002	0.005	0.9993	209.8	74.1	9.4	89.0	4.5	91.1	8.4	95.8	15.4	97.6	15.4	97.8	19.8
116	Fenchlorphos	0.001	0.003	0.9954	53.0	66.2	8.4	77.2	3.7	84.1	16.9	89.5	15.9	86.4	5.8	86.5	8.9
117	Fenclorim	0.001	0.002	0.9998	79.0	53.5	13.4	70.2	5.4	70.8	3.0	51.8	4.8	62.4	17.6	65.5	11.5
118	Fenfuram	0.001	0.004	0.9989	66.8	65.7	10.0	79.9	4.7	88.6	11.2	91.0	17.8	92.7	7.8	95.3	7.7
119	Fenitrothion	0.001	0.004	0.9988	133.7	85.0	5.8	96.3	3.4	95.1	5.4	92.8	9.5	93.1	16.4	101.4	14.8
120	Fenobucarb	0.002	0.005	0.9997	114.3	68.9	9.4	93.7	5.6	94.3	4.7	92.0	16.4	98.5	9.8	97.6	11.4

No.	Analyte	LOD	LOQ	R^2	ME.(%)	Repeatability (*n*=5)						Reproducibility (*n*=11)					
						0.01 mg kg^{-1}		0.02 mg kg^{-1}		0.1 mg kg^{-1}		0.01 mg kg^{-1}		0.02 mg kg^{-1}		0.1 mg kg^{-1}	
						Rec. (%)	CV (%)	Rec. (%)	CV (%)	Rec. (%)	CV (%)	Rec. (%)	CV (%)	Rec. (%)	CV (%)	Rec. (%)	CV (%)
121	Fenothiocarb	0.001	0.003	0.9986	148.6	62.0	10.0	76.5	5.2	85.4	15.0	84.8	23.4	90.2	12.6	92.1	13.7
122	Fenoxanil	0.001	0.004	0.9999	360.6	93.6	6.8	119.2	5.6	119.9	6.1	49.8	31.5	58.0	29.2	57.2	20.9
123	Fenpropathrin	0.001	0.003	0.9989	94.4	73.6	6.6	84.5	3.2	89.0	10.6	79.9	32.2	83.5	28.0	91.6	25.3
124	Fenpropimorph	0.001	0.003	0.9998	56.9	86.2	9.4	70.8	10.7	77.5	7.1	93.0	10.3	81.4	17.9	85.4	17.1
125	Fenpyrazamine	0.002	0.006	0.9991	65.2	74.9	8.4	91.9	9.7	97.4	3.1	88.7	10.5	89.6	15.7	105.2	28.4
126	Fenson	0.001	0.003	0.9990	26.1	86.3	7.0	84.0	9.9	97.3	6.2	84.7	21.7	86.8	16.1	91.2	11.4
127	Fenthion	0.001	0.003	0.9984	58.6	64.8	9.6	77.5	4.4	86.3	14.0	85.2	17.1	87.3	11.4	87.5	16.7
128	Fenvalerate-1	0.001	0.003	0.9998	217.6	89.4	6.6	102.5	4.6	98.9	2.0	699.3	146.6	328.9	115.2	130.1	13.4
	Fenvalerate-2	0.001	0.003	0.9999	308.9	89.0	6.9	103.6	4.6	100.3	1.0	101.2	13.0	101.7	16.2	110.2	23.9
129	Fipronil	0.001	0.004	0.9994	124.0	61.3	9.3	75.7	5.2	86.7	13.6	88.7	29.3	90.7	21.8	95.7	19.4
130	Flamprop-isopropyl	0.001	0.003	1.0000	53.8	68.4	10.4	84.2	3.5	88.6	10.6	94.7	18.0	97.3	7.8	96.1	11.9
131	Fluacrypyrim	0.001	0.003	0.9996	117.4	69.3	9.2	85.0	4.1	89.1	9.9	94.2	18.0	93.3	14.4	96.4	17.5
132	Fluazifop-butyl	0.001	0.003	0.9998	109.7	68.4	9.7	84.1	3.4	88.7	10.6	86.2	21.1	95.8	6.3	91.5	20.0
133	Fluchloralin	0.001	0.003	0.9996	52.9	95.0	5.7	100.8	3.5	96.9	4.7	91.5	20.7	95.4	17.0	96.3	19.6
134	Flucythrinate-1	0.001	0.003	0.9997	186.2	100.9	7.3	117.4	4.1	115.2	4.3	52.0	38.1	61.5	38.7	62.8	25.2
	Flucythrinate-2	0.001	0.004	0.9999	232.3	100.8	7.7	117.1	0.5	116.2	3.9	48.2	41.6	61.5	39.5	71.3	13.1
135	Fluensulfone	0.001	0.004	0.9996	127.8	67.4	9.9	85.8	7.4	90.7	7.3	89.4	14.5	90.1	12.0	96.5	5.4
136	Flufenpyr-ethyl	0.001	0.003	0.9992	92.7	78.9	7.5	95.4	5.4	95.0	3.9	106.6	15.4	108.1	1.7	108.0	9.8
137	Flumetralin	0.001	0.002	0.9997	73.2	85.3	5.7	92.3	3.3	93.2	7.2	90.9	16.8	93.0	15.3	96.6	22.4
138	Flumioxazin	0.002	0.005	0.9998	247.2	82.8	7.9	94.8	5.6	96.3	4.0	89.5	27.9	94.2	22.0	106.9	25.5
139	Fluopyram	0.001	0.004	0.9996	123.4	63.2	10.4	78.3	5.5	87.1	12.9	92.9	22.4	92.4	13.0	96.8	16.8
140	Fluorochloridone	0.002	0.005	0.9999	42.0	92.3	6.9	103.8	5.8	101.0	1.4	98.3	15.5	100.0	18.6	100.6	14.5
141	Fluquinconazole	0.001	0.003	0.9997	29.5	71.3	9.3	85.6	5.2	91.0	8.6	94.6	21.8	95.5	19.2	98.8	20.0

No.	Analyte	LOD	LOQ	R^2	ME.(%)	Repeatability (*n*=5)						Reproducibility (*n*=11)					
						0.01 mg kg^{-1}		0.02 mg kg^{-1}		0.1 mg kg^{-1}		0.01 mg kg^{-1}		0.02 mg kg^{-1}		0.1 mg kg^{-1}	
						Rec. (%)	CV (%)	Rec. (%)	CV (%)	Rec. (%)	CV (%)	Rec. (%)	CV (%)	Rec. (%)	CV (%)	Rec. (%)	CV (%)
142	Flusilazole	0.002	0.005	1.0000	76.5	69.8	10.4	83.2	4.3	88.8	10.8	92.2	16.7	92.9	11.5	96.0	13.7
143	Flutianil	0.001	0.003	0.9995	182.0	79.1	6.9	89.7	2.9	90.7	9.5	97.0	19.1	95.2	23.0	94.2	17.7
144	Fluvalinate-1	0.001	0.003	0.9998	96.1	97.0	7.9	113.0	6.2	106.8	6.3	91.2	60.1	92.8	57.1	107.2	60.5
	Fluvalinate-2	0.001	0.003	0.9999	79.7	95.0	5.4	109.6	6.2	106.2	6.5	90.7	59.7	92.1	55.8	107.3	59.9
145	Fluxapyroxad	0.001	0.004	0.9996	338.9	71.0	9.1	83.2	4.5	89.6	10.5	94.6	20.1	92.5	18.6	93.0	8.7
146	Fonofos	0.001	0.003	0.9996	65.7	63.9	11.9	77.2	4.4	84.5	15.5	82.3	17.5	84.3	15.0	84.5	16.2
147	Formothion	0.003	0.008	0.9996	41.7	91.2	6.7	100.4	9.6	97.6	7.6	93.2	9.2	87.6	24.0	93.3	14.3
148	Fthalide	0.001	0.003	0.9997	31.8	68.2	8.4	82.7	5.3	90.1	10.3	87.7	17.3	89.6	14.0	92.1	11.1
149	Halfenprox	0.001	0.002	0.9990	164.5	70.4	6.7	75.2	4.5	84.3	17.5	71.7	44.2	73.9	38.0	87.7	49.1
150	Heptachlor	0.001	0.002	0.9998	56.4	79.7	14.5	72.2	9.3	72.9	3.3	84.8	19.6	82.2	7.8	83.6	12.3
	Heptachlor epoxide	0.001	0.002	0.9998	31.9	89.1	10.6	80.8	9.8	81.9	19.8	78.9	20.8	82.3	10.9	84.9	13.0
151	Heptenophos	0.001	0.004	0.9993	162.6	77.9	9.2	98.0	7.0	95.3	3.6	91.2	10.8	94.3	19.7	95.6	15.1
152	Hexythiazox	0.001	0.004	0.9994	174.8	60.5	9.7	70.5	3.7	100.5	2.6	85.2	19.6	87.7	12.3	88.3	19.3
153	Indanofan	0.001	0.004	0.9996	162.3	67.5	12.4	85.3	4.7	91.5	6.5	97.4	20.0	92.7	11.3	96.3	14.1
154	Indoxacarb	0.001	0.003	0.9997	231.8	83.6	9.9	105.5	5.8	112.0	7.5	36.3	19.3	48.4	18.6	55.3	4.8
155	Ipconazole	0.001	0.004	0.9979	220.2	66.5	9.6	79.6	4.9	87.7	12.6	92.1	25.4	93.8	25.1	87.9	11.0
156	Iprobenfos	0.001	0.004	0.9998	181.5	75.9	7.0	87.5	4.3	90.7	9.0	94.5	13.8	94.6	13.3	97.6	16.0
157	Iprodione	0.002	0.007	0.9997	178.8	84.8	7.9	102.4	9.9	101.0	1.9	112.9	23.0	107.4	27.7	113.3	27.8
158	Isazofos	0.001	0.003	0.9986	35.4	70.5	9.1	83.4	4.7	88.7	10.7	88.5	14.8	91.7	9.1	92.0	13.4
159	Isofenphos	0.001	0.003	0.9993	52.1	90.8	11.4	72.4	12.2	88.9	7.3	84.6	24.6	84.6	18.5	89.9	19.4
160	Isofenphos-methyl	0.001	0.004	0.9992	53.1	91.8	5.0	79.1	8.6	90.4	6.8	85.3	23.2	85.8	17.9	90.9	17.7
161	Isoprocarb	0.002	0.005	0.9993	135.7	82.2	7.9	98.8	7.4	95.9	3.6	95.5	10.7	100.6	11.7	99.4	12.8
162	Isopropalin	0.001	0.003	0.9995	58.3	61.3	6.6	107.5	2.8	101.4	6.2	87.1	18.9	81.7	10.1	92.5	18.4

No.	Analyte	LOD	LOQ	R^2	ME.(%)	Repeatability (n=5)						Reproducibility (n=11)					
						0.01 mg kg^{-1}		0.02 mg kg^{-1}		0.1 mg kg^{-1}		0.01 mg kg^{-1}		0.02 mg kg^{-1}		0.1 mg kg^{-1}	
						Rec. (%)	CV (%)	Rec. (%)	CV (%)	Rec. (%)	CV (%)	Rec. (%)	CV (%)	Rec. (%)	CV (%)	Rec. (%)	CV (%)
163	Isoprothiolane	0.001	0.003	0.9996	73.4	67.9	10.3	82.2	4.3	88.4	11.4	91.9	16.9	89.8	15.3	93.0	14.8
164	Isopyrazam	0.001	0.003	0.9963	250.7	77.4	8.5	85.4	4.0	89.3	11.3	96.5	18.9	105.0	12.6	93.7	12.7
165	Isotianil	0.001	0.004	0.9997	344.1	88.2	5.8	101.6	5.2	98.3	2.7	102.7	13.2	105.9	10.0	105.5	15.3
166	Isoxadifen-ethyl	0.001	0.003	0.9999	78.2	75.8	7.8	89.1	4.1	91.2	8.0	87.3	16.5	88.9	16.6	93.8	12.9
167	Kresoxim-methyl	0.001	0.003	0.9997	47.0	68.6	11.1	85.9	4.3	89.4	9.6	88.9	18.1	90.8	18.4	94.9	15.8
168	Leptophos	0.001	0.003	0.9994	61.0	107.1	9.7	90.0	2.1	93.7	4.7	82.2	23.1	82.4	12.6	85.2	15.6
169	Mefenpyr-diethyl	0.001	0.004	0.9995	110.8	72.3	9.3	84.1	4.7	88.8	11.1	91.2	19.8	91.3	20.0	96.5	21.3
170	Mepanipyrim	0.001	0.004	0.9992	131.7	61.8	11.0	78.3	4.7	85.3	15.1	87.1	21.5	88.2	14.1	89.9	16.5
171	Mepronil	0.001	0.004	0.9996	207.7	65.5	10.3	81.3	5.5	88.2	11.1	90.2	23.5	90.8	19.5	97.4	16.8
172	Metalaxyl	0.002	0.005	0.9970	43.8	64.5	8.5	77.7	5.4	88.6	11.2	87.0	19.5	89.3	11.7	94.0	8.4
173	Methidathion	0.002	0.005	0.9996	115.0	93.7	6.5	104.1	6.0	100.5	1.8	105.4	7.9	100.6	13.0	101.2	13.8
174	Methoprotryn	0.001	0.004	0.9999	102.4	61.1	11.7	78.6	5.4	87.7	11.6	92.0	23.0	97.1	8.8	98.7	10.8
175	Methoxychlor	0.001	0.002	0.9989	64.7	78.6	18.5	94.0	10.6	100.3	8.2	83.6	9.3	84.6	21.0	85.8	19.1
176	Methyl trithion	0.001	0.003	0.9997	96.4	80.1	8.5	93.8	4.3	93.6	5.6	87.0	9.5	94.2	9.5	93.2	20.0
177	Metolachlor	0.001	0.003	0.9950	55.1	62.9	10.2	78.4	5.0	86.6	13.0	86.6	19.6	86.9	16.3	90.5	13.1
178	Metribuzin	0.001	0.004	0.9996	71.0	70.3	7.3	82.4	4.3	90.3	9.9	96.1	16.3	94.9	4.4	98.1	9.7
179	MGK-264	0.001	0.003	0.9983	43.9	61.1	9.6	73.2	4.5	83.4	18.0	84.2	18.4	87.0	12.0	88.1	14.7
180	Molinate	0.001	0.002	0.9996	52.2	60.6	13.9	75.6	5.0	83.0	17.2	92.9	37.0	84.5	14.7	85.4	11.6
181	Monolinuron	0.003	0.008	0.9997	146.8	83.5	9.3	96.9	5.9	95.4	5.5	100.6	6.7	99.7	9.7	99.8	8.8
182	Myclobutanil	0.001	0.004	0.9999	76.7	68.9	9.4	84.1	4.3	89.5	9.9	90.6	18.5	93.9	12.1	97.1	12.5
183	Nitrothal-isopropyl	0.001	0.002	0.9997	94.8	91.1	4.9	96.6	3.4	95.9	5.5	93.4	16.1	95.8	12.3	92.4	17.6
184	Nonachlor-*cis*	0.001	0.003	0.9998	10.1	107.7	9.1	127.6	3.8	156.0	24.9	73.8	24.3	70.4	32.9	73.1	31.7
	Nonachlor-*trans*	0.001	0.002	0.9980	31.2	105.5	6.5	125.9	3.5	151.9	28.7	63.9	42.0	69.1	32.8	69.9	33.7

No.	Analyte	LOD	LOQ	R^2	ME.(%)	Repeatability (*n*=5)						Reproducibility (*n*=11)					
						0.01 mg kg^{-1}		0.02 mg kg^{-1}		0.1 mg kg^{-1}		0.01 mg kg^{-1}		0.02 mg kg^{-1}		0.1 mg kg^{-1}	
						Rec. (%)	CV (%)	Rec. (%)	CV (%)	Rec. (%)	CV (%)	Rec. (%)	CV (%)	Rec. (%)	CV (%)	Rec. (%)	CV (%)
185	Nuarimol	0.001	0.004	0.9999	158.6	64.7	8.8	80.0	4.6	87.1	12.7	91.4	21.1	93.8	9.9	95.2	16.7
186	Ortho-phenyl phenol	0.001	0.004	0.9989	237.8	64.6	22.3	74.7	6.5	86.6	12.8	90.5	32.7	89.0	16.0	88.8	10.6
187	Oxadiazon	0.001	0.003	0.9989	57.6	67.2	10.4	82.1	2.8	86.9	13.3	83.8	21.0	93.7	7.1	87.9	18.3
188	Oxadixyl	0.001	0.004	0.9998	54.6	74.4	7.4	93.0	4.8	96.6	2.8	96.4	13.5	99.0	9.4	103.0	10.6
189	Oxyfluorfen	0.001	0.003	0.9997	142.1	92.0	7.8	99.6	1.9	95.7	4.2	92.4	19.8	93.9	20.4	94.4	19.4
190	Paclobutrazol	0.002	0.005	0.9989	218.8	60.5	9.0	76.6	5.4	86.9	12.6	88.8	24.3	91.2	15.6	95.9	20.1
191	Parathion	0.001	0.003	0.9996	105.1	84.8	6.8	92.8	3.9	93.0	7.5	94.5	7.0	92.7	13.6	92.7	12.7
192	Parathion-methyl	0.001	0.002	0.9999	77.7	90.3	8.7	102.4	3.4	98.6	2.3	99.6	0.9	96.7	16.2	103.6	13.7
193	Penconazole	0.002	0.005	0.9998	53.5	61.7	16.3	73.3	5.0	84.7	15.9	84.2	22.9	85.5	17.5	90.0	16.0
194	Pendimethalin	0.001	0.003	0.9995	86.5	72.5	7.0	75.8	3.3	84.2	18.4	81.9	18.7	84.7	7.1	96.2	17.2
195	Penflufen	0.001	0.004	0.9996	147.3	65.0	10.6	81.6	4.9	88.6	10.9	90.6	22.7	91.4	17.6	95.4	17.1
196	Pentachlorobenzonitrile	0.001	0.002	0.9998	66.4	63.0	9.8	75.6	4.6	82.8	18.4	74.1	22.3	84.6	5.5	84.3	7.0
197	Penthiopyrad	0.001	0.003	0.9985	187.2	69.1	8.9	83.8	4.6	88.8	10.4	92.5	20.3	93.4	15.2	98.1	18.2
198	Pentoxazone	0.001	0.003	0.9996	96.4	71.7	8.4	85.6	3.4	89.1	10.6	87.2	25.9	99.9	7.8	92.0	22.6
199	Permethrin-cis	0.002	0.005	0.9995	170.6	81.9	16.5	76.1	8.4	93.7	10.9	71.3	43.7	80.2	36.6	79.3	42.9
	Permethrin-trans	0.001	0.003	0.9992	160.6	61.5	4.1	70.2	6.3	90.6	16.8	80.4	28.9	80.6	31.5	83.5	37.7
200	Perthane	0.001	0.003	0.9999	47.6	60.5	8.5	73.7	2.9	83.4	17.1	87.8	20.3	87.6	9.1	91.0	11.9
201	Phenthoate	0.001	0.003	0.9996	61.1	78.3	8.2	88.3	3.2	90.4	9.8	88.2	13.8	90.3	14.3	96.9	20.6
202	Phosphamidon (*E*)	0.001	0.004	0.9954	>500	100.9	9.5	118.3	12.3	101.5	2.7	98.5	23.3	94.2	21.7	110.9	6.6
	Phosphamidon (*Z*)	0.002	0.006	0.9998	219.6	94.1	7.1	108.2	6.3	102.8	2.5	94.4	25.1	98.2	25.6	112.7	7.4
203	Phosalone	0.002	0.007	0.9990	132.4	94.3	9.3	110.4	5.4	104.0	5.2	94.7	17.1	96.1	23.4	104.3	21.9
204	Phosmet	0.002	0.007	0.9993	41.1	87.8	5.6	112.0	7.1	108.7	6.7	100.6	4.7	100.4	17.2	91.6	20.7
205	Picoxystrobin	0.001	0.004	0.9981	119.4	68.7	9.2	85.7	3.3	88.0	11.3	89.1	19.0	90.4	18.3	93.4	14.8

No.	Analyte	LOD	LOQ	R^2	ME.(%)	Repeatability (*n*=5)						Reproducibility (*n*=11)					
						0.01 mg kg^{-1}		0.02 mg kg^{-1}		0.1 mg kg^{-1}		0.01 mg kg^{-1}		0.02 mg kg^{-1}		0.1 mg kg^{-1}	
						Rec. (%)	CV (%)	Rec. (%)	CV (%)	Rec. (%)	CV (%)	Rec. (%)	CV (%)	Rec. (%)	CV (%)	Rec. (%)	CV (%)
206	Piperonyl butoxide	0.001	0.004	0.9992	194.7	71.2	10.0	81.9	4.4	87.8	12.5	89.8	21.0	89.8	20.0	94.2	22.0
207	Pirimicarb	0.001	0.003	0.9958	59.4	64.2	8.8	78.6	4.3	88.0	11.5	87.2	17.5	89.8	9.7	92.3	9.3
208	Pirimiphos-ethyl	0.001	0.003	0.9983	52.1	110.3	8.8	70.5	9.6	81.3	7.3	76.9	26.0	86.9	12.0	91.1	13.9
209	Pirimiphos-methyl	0.001	0.003	0.9957	58.4	68.4	9.0	76.9	3.7	85.0	16.0	87.2	14.4	89.8	8.2	85.8	18.0
210	Pretilachlor	0.001	0.004	0.9990	91.7	76.5	8.9	90.8	4.1	91.0	8.0	98.0	11.6	99.2	4.9	97.9	7.6
211	Prochloraz	0.001	0.004	0.9985	387.8	65.4	14.4	84.2	3.5	93.3	5.8	90.1	17.0	96.9	11.0	112.3	25.1
	2,4,6-trichlorophenol	0.002	0.007	0.9952	127.6	60.7	9.9	73.3	4.5	81.5	20.3	61.3	9.2	69.0	20.6	64.0	31.2
212	Procymidone	0.001	0.003	0.9993	35.9	62.4	11.0	76.8	4.7	86.1	14.7	82.4	22.6	87.7	14.2	90.4	13.4
213	Prodiamine	0.001	0.002	0.9997	77.7	76.4	6.8	82.9	3.4	87.4	13.6	94.5	8.8	92.2	4.6	100.5	14.1
214	Profenofos	0.002	0.005	0.9997	267.5	75.0	8.5	96.2	5.4	97.1	2.1	91.3	17.0	94.6	20.4	98.4	21.3
215	Profluralin	0.001	0.002	0.9948	54.5	81.6	6.8	85.6	3.7	87.6	13.7	90.9	7.0	87.5	14.4	89.4	19.6
216	Prohydrojasmon	0.001	0.002	0.9949	70.6	64.6	12.5	76.1	4.4	85.5	16.1	90.0	17.5	88.8	9.8	88.3	12.9
217	Prometon	0.001	0.004	0.9997	110.0	61.4	9.8	76.6	4.6	86.4	13.5	87.6	19.8	93.1	7.5	93.9	6.8
218	Prometryn	0.001	0.003	0.9995	51.4	113.5	10.4	75.7	9.0	86.8	10.1	80.6	24.6	82.0	17.4	86.2	15.6
219	Propachlor	0.001	0.004	0.9994	95.9	75.8	8.3	93.2	5.7	92.7	5.7	94.7	9.7	95.9	10.1	95.0	10.1
220	Propanil	0.001	0.004	0.9998	95.6	68.4	7.3	78.2	4.8	87.0	14.0	94.6	17.3	96.2	8.7	96.5	9.7
221	Propazine	0.001	0.003	0.9997	81.6	63.3	10.5	78.7	5.0	86.7	13.2	84.5	17.9	85.9	13.8	89.7	9.3
222	Propetamphos	0.001	0.003	0.9994	97.8	71.2	10.1	83.3	3.8	88.4	11.4	88.1	15.0	89.4	15.5	92.6	12.9
223	Propham	0.001	0.003	0.9992	142.5	64.0	12.1	82.9	5.0	87.2	11.4	88.5	17.5	92.8	7.7	91.5	5.8
224	Propiconazole-1	0.002	0.005	0.9999	95.6	64.3	9.1	79.4	5.4	87.2	12.9	81.5	23.0	88.8	18.9	94.5	16.5
	Propiconazole-2	0.002	0.006	1.0000	104.4	66.8	9.7	81.2	4.1	87.5	12.1	88.0	16.5	91.1	15.7	94.2	17.1
225	Propisochlor	0.001	0.003	0.9952	42.0	72.1	9.6	83.1	4.9	88.3	11.7	88.0	15.7	87.6	16.9	90.8	14.5
226	Propyzamide	0.001	0.003	0.9998	89.4	66.1	9.4	79.3	5.2	86.7	13.1	92.5	18.0	90.7	7.7	91.5	12.7

No.	Analyte	LOD	LOQ	R^2	ME.(%)	Repeatability (*n*=5)						Reproducibility (*n*=11)					
						0.01 mg kg^{-1}		0.02 mg kg^{-1}		0.1 mg kg^{-1}		0.01 mg kg^{-1}		0.02 mg kg^{-1}		0.1 mg kg^{-1}	
						Rec. (%)	CV (%)	Rec. (%)	CV (%)	Rec. (%)	CV (%)	Rec. (%)	CV (%)	Rec. (%)	CV (%)	Rec. (%)	CV (%)
227	Prothiofos	0.001	0.003	0.9981	93.0	105.8	8.6	98.6	3.9	101.3	4.7	84.3	24.7	82.3	13.0	85.2	15.3
228	Pyracarbolid	0.002	0.005	0.9997	113.2	113.3	10.0	76.4	7.6	89.5	7.8	87.5	26.8	92.4	13.0	97.5	12.3
229	Pyraclofos	0.001	0.003	0.9989	496.7	96.5	9.8	124.4	0.7	131.5	4.3	56.6	9.8	62.5	18.4	64.6	11.3
230	Pyraflufen-ethyl	0.001	0.004	1.0000	126.2	71.6	7.8	86.0	3.9	90.2	9.3	92.8	18.5	91.2	19.8	97.5	17.0
231	Pyrazophos	0.001	0.004	0.9980	112.0	82.9	8.6	97.2	4.8	96.3	3.0	96.0	11.6	96.2	17.2	101.2	17.2
232	Pyridalyl	0.001	0.003	0.9997	226.1	105.8	7.7	119.6	5.8	113.3	15.7	65.0	54.9	69.3	44.9	75.8	52.0
233	Pyrifenox	0.001	0.004	0.9999	68.9	60.8	11.9	74.1	5.5	84.7	15.7	83.2	28.8	91.0	12.8	94.9	15.6
234	Pyriftalid	0.001	0.004	0.9992	164.2	77.2	8.2	89.3	5.2	92.6	7.9	96.8	16.2	98.3	13.5	98.5	13.4
235	Pyrimethanil	0.001	0.003	0.9956	65.0	119.6	10.8	79.0	9.4	90.1	8.4	81.0	20.6	83.3	15.3	85.5	12.1
236	Pyriminobac-methyl (*E*)	0.001	0.003	0.9995	109.4	68.5	9.1	83.9	5.4	89.8	9.7	91.3	17.9	91.9	14.1	90.6	5.9
	Pyriminobac-methyl (*Z*)	0.001	0.004	0.9998	65.5	68.4	9.1	84.7	5.2	90.2	9.2	91.7	17.7	91.9	13.8	97.7	16.0
237	Quinalphos	0.001	0.004	0.9998	58.4	65.2	7.7	78.5	5.2	86.9	13.3	82.7	20.6	87.7	14.8	90.3	17.3
238	Quinoxyfen	0.001	0.003	1.0000	47.8	101.5	11.3	94.1	2.9	108.1	4.7	86.2	25.8	85.2	15.9	87.2	17.5
239	Quintozene	0.001	0.003	0.9991	75.5	95.6	8.2	102.0	4.7	93.9	6.6	91.6	14.7	87.6	25.7	82.4	20.2
240	Quizalofop-ethyl	0.001	0.003	0.9985	216.6	77.9	7.9	87.2	3.1	89.8	11.0	77.6	3.0	81.8	20.4	84.0	12.0
241	Silafluofen	0.001	0.003	0.9986	144.6	111.2	7.6	94.7	8.8	91.9	4.1	88.6	26.2	88.0	18.9	90.1	19.6
242	Simeconazole	0.001	0.004	0.9990	104.3	66.8	7.8	78.9	4.6	87.6	12.6	91.3	19.1	91.6	13.8	94.5	15.2
243	Simetryn	0.001	0.004	1.0000	66.5	61.9	8.3	74.6	5.0	85.7	14.9	86.0	20.5	89.1	9.8	91.3	10.3
244	Spiromesifen	0.001	0.003	0.9991	93.5	70.6	11.8	86.0	5.2	90.2	7.4	87.6	22.6	97.2	7.5	101.4	7.7
245	Spiroxamine-1	0.002	0.005	0.9997	95.0	88.1	18.8	70.4	15.0	83.1	7.8	103.7	9.4	93.0	16.8	93.2	12.1
	Spiroxamine-2	0.001	0.004	0.9990	70.6	97.9	15.6	71.8	15.8	76.5	11.0	97.2	30.8	97.2	16.5	95.9	13.8
246	Sulfotep	0.001	0.003	0.9993	80.7	69.1	10.5	83.4	4.1	87.3	12.5	85.4	15.6	92.0	7.4	90.1	12.2
247	Tebuconazole	0.002	0.005	0.9998	249.2	65.7	9.9	79.7	4.7	87.4	12.3	94.6	21.3	95.8	12.1	91.8	9.9

No.	Analyte	LOD	LOQ	R^2	ME.(%)	Repeatability (*n*=5)						Reproducibility (*n*=11)					
						0.01 mg kg^{-1}		0.02 mg kg^{-1}		0.1 mg kg^{-1}		0.01 mg kg^{-1}		0.02 mg kg^{-1}		0.1 mg kg^{-1}	
						Rec. (%)	CV (%)	Rec. (%)	CV (%)	Rec. (%)	CV (%)	Rec. (%)	CV (%)	Rec. (%)	CV (%)	Rec. (%)	CV (%)
248	Tebufenpyrad	0.001	0.003	0.9992	108.9	64.9	8.6	76.9	3.9	85.0	15.7	92.1	20.5	94.8	10.4	97.7	14.1
249	Tebupirimfos	0.001	0.003	0.9997	68.2	62.9	9.6	74.0	3.5	82.9	18.0	81.8	17.4	87.4	9.7	83.9	18.3
250	Tecnazene	0.001	0.003	0.9999	71.4	80.6	9.1	92.1	3.8	88.3	10.4	87.2	8.0	91.1	11.9	90.1	7.8
251	Tefluthrin	0.001	0.002	0.9993	50.4	63.5	9.5	75.6	2.8	84.3	15.8	88.4	17.5	87.0	9.0	87.8	9.8
252	Terbacil	0.002	0.005	0.9998	194.0	78.8	6.0	93.7	4.3	96.8	3.6	101.4	11.9	101.4	4.3	104.3	3.9
253	Terbumeton	0.001	0.003	0.9996	92.7	62.4	9.5	76.5	4.8	86.1	14.1	93.5	22.4	86.9	16.5	91.1	7.2
254	Terbutryn	0.001	0.003	0.9997	70.5	118.2	11.2	77.2	8.7	89.4	7.6	82.1	24.6	84.3	17.8	86.4	15.8
255	Tetrachlorvinphos	0.002	0.005	0.9989	208.0	85.4	10.0	100.1	7.0	99.0	4.8	98.7	10.6	97.7	18.4	99.6	19.3
256	Tetraconazole	0.001	0.004	0.9995	63.6	61.4	9.2	75.6	4.1	87.1	13.0	87.3	24.0	88.0	16.8	93.4	15.7
257	Tetradifon	0.001	0.003	1.0000	47.7	65.6	7.6	76.8	3.7	84.7	16.3	79.6	27.2	92.1	9.4	94.5	11.8
258	Tetramethrin-1	0.002	0.005	0.9999	372.2	85.7	5.9	96.1	2.6	93.6	5.8	85.8	39.0	86.6	41.3	97.6	25.2
	Tetramethrin-2	0.002	0.006	0.9993	194.6	79.8	15.7	77.6	9.2	90.8	7.7	81.0	11.1	87.4	27.6	88.3	25.3
259	Thifluzamide	0.001	0.004	0.9999	238.9	69.7	10.3	86.8	4.9	89.8	9.5	93.4	18.4	93.8	16.0	99.1	19.0
260	Thiometon	0.001	0.003	0.9995	103.7	61.7	10.7	74.1	3.4	85.2	14.2	84.3	20.1	86.4	10.9	89.7	11.9
261	Thionazin	0.001	0.003	0.9995	88.0	65.7	11.6	82.7	5.0	87.7	10.3	96.2	22.1	89.9	10.8	91.1	9.7
262	Tolclofos-methyl	0.001	0.003	0.9998	61.3	68.6	7.5	80.5	4.0	86.1	14.2	83.3	15.7	90.8	6.7	86.0	15.7
263	Triadimefon	0.001	0.004	0.9990	58.4	63.5	8.6	76.5	5.4	86.6	14.3	95.5	22.6	92.7	9.8	94.0	11.8
264	Triadimenol	0.002	0.005	0.9999	128.3	64.8	9.0	78.7	5.2	88.2	12.5	93.8	21.1	101.0	12.0	91.3	11.0
265	Tri-allate	0.001	0.003	0.9997	58.8	93.3	14.4	78.1	9.9	76.2	9.8	85.3	21.6	82.9	11.4	83.2	14.5
266	Triazophos	0.001	0.004	0.9995	160.1	81.1	10.1	98.3	5.9	96.8	2.0	104.2	13.4	98.2	12.9	103.0	19.9
267	Tridiphane	0.001	0.003	0.9990	59.5	78.2	9.7	89.7	6.2	83.5	6.0	56.7	18.3	64.6	22.4	66.7	11.6
268	Trifloxystrobin	0.001	0.003	0.9997	88.1	72.7	8.0	87.7	3.4	89.7	9.4	88.6	19.4	100.2	3.3	95.6	21.3
269	Triflumizole	0.001	0.004	0.9999	74.9	65.1	12.2	76.5	3.3	85.5	14.9	85.3	19.6	86.1	19.0	91.2	17.8

No.	Analyte	LOD	LOQ	R^2	ME.(%)	Repeatability (*n*=5)						Reproducibility (*n*=11)					
						0.01 mg kg^{-1}		0.02 mg kg^{-1}		0.1 mg kg^{-1}		0.01 mg kg^{-1}		0.02 mg kg^{-1}		0.1 mg kg^{-1}	
						Rec. (%)	CV (%)	Rec. (%)	CV (%)	Rec. (%)	CV (%)	Rec. (%)	CV (%)	Rec. (%)	CV (%)	Rec. (%)	CV (%)
270	Trifluralin	0.001	0.002	0.9993	104.7	76.5	8.1	84.8	2.7	86.8	13.5	82.9	19.2	89.2	9.3	94.4	12.6
271	Vinclozolin	0.001	0.002	0.9996	50.8	67.6	8.7	79.3	5.4	87.1	13.7	88.2	14.9	90.8	6.5	88.8	12.6
272	Zoxamide	0.002	0.006	0.9995	138.9	89.1	15.8	108.4	7.3	104.3	4.1	89.1	15.5	103.2	14.4	99.5	22.4

Table S4. Method validation parameter of 272 pesticides in potato.

No.	Analyte	LOD	LOQ	R^2	ME.(%)	Repeatability (*n*=5)						Reproducibility (*n*=11)					
						0.01 mg kg^{-1}		0.02 mg kg^{-1}		0.1 mg kg^{-1}		0.01 mg kg^{-1}		0.02 mg kg^{-1}		0.1 mg kg^{-1}	
						Rec. (%)	CV (%)	Rec. (%)	CV (%)	Rec. (%)	CV (%)	Rec. (%)	CV (%)	Rec. (%)	CV (%)	Rec. (%)	CV (%)
1	2,6-DIPN	0.002	0.006	0.9997	47.5	129.8	11.8	103.1	6.0	81.5	4.8	138.2	5.0	118.0	8.4	90.7	0.2
2	Acetochlor	0.002	0.007	0.9998	31.2	78.5	4.6	78.8	4.5	75.7	1.5	98.0	13.2	92.6	6.3	94.5	10.9
	EMA	0.003	0.008	0.9931	10.6	72.3	13.2	77.6	21.0	88.6	7.5	88.1	17.0	88.9	4.2	92.7	8.4
	HEMA	0.002	0.007	0.9989	373.7	68.8	6.5	66.0	7.4	65.9	2.1	116.3	42.2	62.5	57.6	66.2	39.6
3	Acrinathrin	0.003	0.008	0.9999	55.7	52.4	11.7	72.1	28.0	51.0	4.3	101.2	37.6	90.7	28.1	99.4	41.2
4	Alachlor	0.002	0.007	1.0000	27.6	79.1	4.8	80.2	4.5	78.2	1.3	95.6	12.7	89.4	1.6	90.5	13.3
5	Aldrin	0.002	0.007	0.9991	16.3	86.3	1.7	88.4	3.8	82.8	1.4	88.4	8.1	87.5	10.8	86.8	5.2
	Dieldrin	0.002	0.007	0.9998	11.5	89.0	2.2	91.3	2.0	85.3	0.4	95.6	3.3	93.3	8.0	94.4	1.8
6	Allidochlor	0.003	0.008	0.9970	28.3	75.8	7.3	86.0	5.9	79.9	5.6	95.4	11.7	91.0	5.4	90.8	5.5
7	Ametryn	0.002	0.007	1.0000	54.9	77.0	3.4	78.5	2.6	75.8	0.7	94.6	12.4	87.9	2.4	88.7	12.6
8	Anilofos	0.003	0.008	0.9999	43.2	70.3	14.4	73.9	15.1	72.5	7.4	100.0	21.0	96.2	13.3	102.1	18.6
9	Aramite	0.002	0.007	1.0000	46.1	77.6	7.6	78.5	6.9	72.2	3.1	97.8	17.7	90.7	7.7	91.9	20.0
10	Aspon	0.001	0.003	0.9993	55.5	80.3	3.9	80.8	3.5	76.3	1.4	96.0	9.8	91.1	1.7	93.2	9.3
11	Atrazine	0.002	0.006	0.9996	54.0	78.6	6.6	81.1	5.7	76.3	1.2	98.3	10.5	91.5	3.3	92.2	7.8
12	Azaconazole	0.001	0.004	1.0000	26.6	82.8	4.4	85.2	2.8	81.7	0.9	104.1	10.1	98.9	5.7	98.9	7.6
13	Benfluralin	0.002	0.007	0.9999	46.9	81.7	3.0	82.4	3.3	77.3	3.1	97.6	15.2	88.2	3.3	92.3	10.6
14	Benfuresate	0.003	0.008	0.9997	23.9	80.5	2.8	82.4	2.5	78.9	1.2	98.4	10.5	92.6	2.2	92.5	8.5
15	Benodanil	0.002	0.007	0.9999	69.4	70.4	3.1	72.3	4.7	70.5	2.3	93.3	24.8	89.5	12.9	89.1	28.6
16	Benzoylprop-ethyl	0.003	0.008	0.9998	34.8	78.1	4.2	78.1	3.2	76.9	0.7	101.1	13.7	96.1	8.9	95.8	13.5
17	BHC, α-	0.002	0.007	0.9990	12.9	86.2	4.5	95.8	5.9	90.0	3.4	99.2	11.1	94.9	10.5	93.3	10.1
	BHC, β-	0.002	0.007	1.0000	15.5	100.7	5.6	110.1	4.1	116.1	5.2	105.3	10.4	100.7	16.2	94.8	5.7

No.	Analyte	LOD	LOQ	R^2	ME.(%)	Repeatability (n=5)						Reproducibility (n=11)					
						0.01 mg kg^{-1}		0.02 mg kg^{-1}		0.1 mg kg^{-1}		0.01 mg kg^{-1}		0.02 mg kg^{-1}		0.1 mg kg^{-1}	
						Rec. (%)	CV (%)	Rec. (%)	CV (%)	Rec. (%)	CV (%)	Rec. (%)	CV (%)	Rec. (%)	CV (%)	Rec. (%)	CV (%)
	BHC, *δ*-	0.002	0.007	0.9987	-28.2	66.0	7.6	77.7	10.5	78.5	4.5	90.2	21.7	88.8	2.5	90.0	7.3
18	BHC, *γ*-	0.002	0.007	0.9989	8.4	96.4	8.9	106.3	5.9	108.0	2.2	98.6	7.6	104.6	11.4	102.2	15.6
19	Bifenox	0.002	0.007	0.9992	43.0	91.6	0.8	84.1	6.6	77.1	3.1	106.3	14.1	101.7	8.6	104.2	15.5
20	Bifenthrin	0.003	0.008	0.9998	59.5	80.0	4.8	78.9	3.6	75.1	1.5	96.8	9.4	92.9	5.5	90.8	10.1
21	Boscalid	0.002	0.007	1.0000	94.7	92.6	4.7	91.8	1.6	83.9	6.8	84.9	26.4	99.5	24.8	90.6	24.9
22	Bromobutide	0.003	0.008	0.9998	23.0	90.3	4.8	90.2	2.5	87.9	1.5	99.5	10.8	94.3	6.2	97.1	9.3
23	Bromophos-methyl	0.002	0.006	0.9999	31.9	81.2	5.8	83.2	4.3	80.0	0.8	93.0	10.8	89.0	10.1	88.1	8.7
24	Bromophos-ethyl	0.002	0.007	1.0000	37.9	73.4	7.4	74.0	7.1	71.5	2.1	97.2	6.6	92.3	2.7	92.1	4.9
25	Bromopropylate	0.003	0.008	0.9994	98.8	72.3	5.5	71.4	3.4	70.0	1.7	101.1	19.2	95.5	14.7	95.0	19.5
26	Bupirimate	0.002	0.006	1.0000	47.4	80.2	5.3	78.9	2.9	78.2	0.9	101.6	11.8	94.7	6.5	94.5	11.6
27	Buprofezin	0.002	0.007	1.0000	28.9	81.6	4.1	81.5	3.5	79.4	0.7	95.7	9.7	90.5	3.9	92.5	8.8
28	Butachlor	0.002	0.007	1.0000	42.5	78.7	4.4	76.8	4.6	72.6	1.4	99.7	11.3	91.5	5.9	92.3	15.7
29	Butafenacil	0.003	0.009	0.9993	115.7	61.9	6.4	96.9	2.7	70.5	1.2	100.6	31.5	96.1	24.3	96.8	22.9
30	Butralin	0.002	0.006	0.9993	47.1	83.6	5.4	78.2	2.8	74.7	2.0	90.5	10.2	88.6	3.5	95.7	15.9
31	Butylate	0.003	0.008	0.9950	31.3	77.7	2.0	87.4	4.7	79.3	7.2	89.3	10.7	89.0	11.6	89.6	3.9
32	Cadusafos	0.003	0.008	0.9995	81.5	71.2	4.8	75.6	3.6	73.1	3.0	94.7	15.5	88.2	4.7	89.8	11.3
33	Carbophenothion	0.002	0.007	1.0000	59.3	70.5	8.0	83.8	2.3	72.2	8.5	99.2	19.0	88.0	11.5	91.6	20.0
34	Carboxin	0.001	0.004	1.0000	59.1	75.5	4.2	75.5	3.3	75.0	1.3	105.9	18.1	98.1	13.2	105.4	18.1
35	Carfentrazone-ethyl	0.002	0.007	0.9999	46.5	97.2	5.6	94.6	7.5	94.4	4.0	91.1	20.1	92.9	30.2	92.7	33.0
36	Chinomethionat	0.002	0.007	1.0000	5.0	70.4	11.9	76.3	4.8	75.6	6.9	77.4	34.9	74.9	38.3	70.0	63.1
37	Chlorbenside	0.002	0.006	0.9995	28.7	80.6	3.5	82.7	2.9	78.4	1.1	91.9	5.6	86.5	6.7	86.1	8.8
38	Chlorbufam	0.002	0.007	0.9997	86.3	71.2	10.3	72.0	5.9	77.2	10.5	80.4	2.8	80.0	11.3	86.1	21.2
39	Chlordane-*cis*	0.002	0.005	0.9991	19.2	85.8	0.7	92.0	2.3	87.7	0.8	95.9	0.5	95.5	7.7	93.4	4.3

No.	Analyte	LOD	LOQ	R^2	ME.(%)	Repeatability (*n*=5)						Reproducibility (*n*=11)					
						0.01 mg kg^{-1}		0.02 mg kg^{-1}		0.1 mg kg^{-1}		0.01 mg kg^{-1}		0.02 mg kg^{-1}		0.1 mg kg^{-1}	
						Rec. (%)	CV (%)	Rec. (%)	CV (%)	Rec. (%)	CV (%)	Rec. (%)	CV (%)	Rec. (%)	CV (%)	Rec. (%)	CV (%)
	Chlordane-*trans*	0.002	0.007	0.9997	12.9	89.3	1.9	93.5	3.5	89.7	1.0	97.8	1.5	95.8	8.5	92.8	7.7
40	Chlorethoxyfos	0.003	0.008	0.9988	22.7	83.2	8.4	97.8	7.5	100.2	5.1	98.6	7.9	98.3	12.4	102.6	8.1
41	Chlorfenapyr	0.003	0.009	0.9999	33.9	80.5	4.7	80.4	6.4	76.4	1.8	95.0	9.4	93.1	4.6	94.7	10.4
42	Chlorfenson	0.002	0.006	0.9993	26.2	81.6	4.4	83.1	4.2	77.4	1.2	96.8	9.0	91.2	4.8	90.4	11.5
43	Chlorflurenol-methyl	0.002	0.006	1.0000	89.3	66.8	6.0	95.0	3.0	77.0	7.0	97.6	21.9	91.8	13.4	94.2	18.5
44	Chlornitrofen	0.002	0.007	0.9997	44.5	81.4	4.9	80.3	3.9	72.5	0.9	99.0	11.7	93.4	3.9	96.0	15.8
45	Chlorobenzilate	0.002	0.007	0.9997	51.4	75.5	4.3	78.2	3.0	76.8	1.3	99.3	15.7	94.3	7.0	94.5	12.3
46	Chloropropylate	0.003	0.008	0.9998	49.1	75.5	4.3	78.2	3.0	76.8	1.3	99.4	15.7	92.1	6.9	93.5	13.1
47	Chloroneb	0.002	0.007	0.9969	30.0	84.6	2.5	93.2	3.2	85.2	4.7	89.1	5.1	92.8	10.5	97.6	6.3
48	Chlorothalonil	0.001	0.004	0.9455	-21.1	73.9	10.2	70.1	22.6	54.0	11.7	58.1	83.4	80.2	11.9	115.3	62.7
49	Chlorpropham	0.003	0.008	0.9987	81.8	82.7	5.1	81.5	4.1	74.4	2.1	102.2	8.7	92.3	8.1	93.3	10.5
50	Chlorpyrifos	0.002	0.007	0.9994	32.4	79.8	4.3	81.1	4.4	76.3	2.1	94.9	7.7	92.3	4.6	91.0	7.9
51	Chlorpyrifos-methyl	0.002	0.007	0.9999	24.3	73.8	6.3	75.5	7.4	71.9	2.3	96.5	13.2	89.9	8.6	89.1	9.3
52	Chlorthal-dimethyl	0.002	0.007	0.9996	22.5	82.9	3.8	85.7	3.3	80.4	1.1	98.1	6.3	94.1	3.3	93.4	5.8
53	Chlorthion	0.002	0.007	0.9997	105.8	63.6	13.7	87.6	2.8	72.3	9.4	95.9	25.7	84.5	12.9	93.0	16.9
54	Chlorthiophos	0.002	0.007	1.0000	38.0	74.3	6.7	75.5	5.4	73.0	1.3	96.3	13.0	91.5	7.3	91.8	12.1
55	Chlozolinate	0.002	0.007	1.0000	19.8	63.7	9.0	70.2	2.8	70.3	4.0	77.7	39.6	79.6	16.8	83.2	23.5
56	Cinmethylin	0.003	0.008	0.9998	24.5	74.9	3.8	79.8	2.7	77.7	1.5	95.7	21.9	93.7	4.9	90.6	6.1
57	Clomazone	0.002	0.007	0.9996	44.6	96.8	4.3	101.5	4.8	99.8	1.7	98.9	9.9	94.3	17.2	97.2	15.3
58	Coumaphos	0.003	0.008	0.9999	51.0	68.5	17.2	96.9	7.4	84.6	8.5	95.9	18.2	95.4	19.8	92.9	23.6
59	Cyanophos	0.003	0.008	0.9993	35.7	75.1	10.5	73.9	7.6	90.2	9.4	95.0	11.2	90.8	14.6	90.7	13.8
60	Cyflufenamid	0.002	0.007	0.9999	67.8	83.0	4.6	85.8	3.8	83.3	1.1	98.2	7.7	96.5	1.6	99.6	12.9
61	Cyfluthrin	0.003	0.009	0.9989	102.2	53.0	10.4	62.3	5.8	60.5	5.1	87.1	33.3	88.0	25.3	96.8	36.8

No.	Analyte	LOD	LOQ	R^2	ME.(%)	Repeatability (*n*=5)						Reproducibility (*n*=11)					
						0.01 mg kg^{-1}		0.02 mg kg^{-1}		0.1 mg kg^{-1}		0.01 mg kg^{-1}		0.02 mg kg^{-1}		0.1 mg kg^{-1}	
						Rec. (%)	CV (%)	Rec. (%)	CV (%)	Rec. (%)	CV (%)	Rec. (%)	CV (%)	Rec. (%)	CV (%)	Rec. (%)	CV (%)
62	Cyhalofop-butyl	0.003	0.008	0.9999	108.5	54.9	7.0	57.0	5.0	60.7	2.0	53.4	49.4	67.2	53.1	77.0	50.9
63	Cyhalothrin, *γ*-	0.003	0.008	0.9996	78.3	79.5	6.6	72.4	4.5	62.3	4.3	96.0	6.6	91.2	10.7	89.2	18.7
	Cyhalothrin, *λ*-	0.003	0.009	1.0000	118.7	56.2	8.0	58.5	6.1	59.4	2.6	82.9	21.6	87.0	7.1	85.3	21.6
64	Cypermethrin	0.002	0.007	0.9999	54.5	56.0	8.8	61.8	6.2	62.1	3.5	87.1	32.4	87.7	24.3	98.2	38.7
65	Cyprazine	0.002	0.005	1.0000	29.7	80.1	4.0	78.7	3.8	74.4	1.3	94.0	8.3	90.7	4.5	95.2	11.7
66	Cyprodinil	0.002	0.007	0.9999	41.6	81.1	3.4	83.3	2.9	79.1	0.7	98.4	7.6	94.4	7.0	94.0	6.2
67	DDD, *p,p'*-	0.002	0.007	1.0000	2.4	91.7	3.1	93.6	2.4	90.4	1.1	101.0	5.0	98.5	4.8	98.8	6.4
	DDE, *p,p'*-	0.001	0.004	0.9998	22.9	81.4	3.2	85.9	1.9	79.7	0.8	89.3	7.5	89.8	8.3	87.6	1.6
	DDT, *o,p'*-	0.002	0.006	0.9994	-1.6	90.0	9.9	112.2	25.8	126.6	20.2	95.6	4.7	95.8	8.4	82.2	12.1
	DDT, *p,p'*-	0.003	0.008	0.9990	-4.4	98.3	9.3	107.3	32.5	101.7	28.5	103.1	5.1	99.6	9.4	107.8	9.1
68	Deltamethrin	0.003	0.008	0.9988	119.5	77.0	10.5	81.7	3.3	75.2	5.2	77.9	17.4	75.0	23.2	79.0	5.0
69	Desmetryn	0.002	0.007	1.0000	46.4	82.0	3.8	82.2	3.3	78.2	2.0	97.0	5.6	92.7	7.8	94.3	7.8
70	Dialifor	0.003	0.008	1.0000	20.1	71.8	10.6	71.3	6.8	70.2	2.5	98.8	17.4	92.7	12.7	97.6	21.7
71	Di-Allate	0.003	0.008	0.9991	39.9	77.8	1.6	84.4	3.9	78.7	2.9	90.9	5.7	86.6	7.2	87.9	9.7
72	Diazinon	0.003	0.008	0.9998	33.2	83.0	3.9	84.8	2.9	80.6	2.1	95.2	3.4	93.7	8.1	96.5	6.8
73	Dichlobenil	0.002	0.006	0.9956	11.7	78.9	4.8	91.3	4.2	86.3	4.3	94.7	6.7	94.0	10.5	93.0	4.7
74	Dichlofenthion	0.002	0.007	0.9998	35.1	79.3	3.0	82.5	3.8	79.2	1.7	94.4	5.8	90.3	4.9	90.8	6.3
75	Dichlofluanid	0.001	0.003	0.9319	65.1	75.5	4.9	77.6	2.3	71.8	5.6	97.7	33.4	87.9	19.6	111.4	66.0
76	Dichlormid	0.003	0.008	0.9974	33.8	85.3	9.8	100.3	6.1	98.1	5.4	97.5	4.0	98.5	13.4	104.1	4.1
77	Diclobutrazol	0.001	0.004	1.0000	195.1	64.1	7.3	87.1	2.1	70.4	2.1	94.2	21.7	91.1	16.6	82.9	9.1
78	Diclofop-methyl	0.002	0.007	0.9998	66.3	67.9	2.3	98.6	12.9	88.2	14.8	77.1	32.4	87.4	14.2	97.7	13.2
79	Dicloran	0.003	0.008	0.9999	64.6	72.7	10.6	73.1	11.2	94.8	5.6	91.7	11.5	92.0	15.8	91.8	17.5
80	Dicofol	0.002	0.006	1.0000	28.7	65.7	4.4	85.4	3.0	97.3	3.9	88.7	15.5	92.5	6.2	98.8	8.6

No.	Analyte	LOD	LOQ	R^2	ME.(%)	Repeatability (n=5)						Reproducibility (n=11)					
						0.01 mg kg^{-1}		0.02 mg kg^{-1}		0.1 mg kg^{-1}		0.01 mg kg^{-1}		0.02 mg kg^{-1}		0.1 mg kg^{-1}	
						Rec. (%)	CV (%)	Rec. (%)	CV (%)	Rec. (%)	CV (%)	Rec. (%)	CV (%)	Rec. (%)	CV (%)	Rec. (%)	CV (%)
81	Dicrotophos	0.002	0.007	0.9988	187.2	64.6	13.9	71.2	19.8	88.6	11.7	93.7	20.7	84.6	5.6	93.3	13.7
82	Diethatyl-ethyl	0.002	0.007	0.9995	50.3	87.4	5.1	86.8	5.0	82.2	1.1	101.0	5.1	98.2	4.8	101.3	10.5
83	Diethofencarb	0.002	0.007	0.9999	155.7	69.5	6.8	89.5	2.2	73.8	8.9	97.6	18.2	91.8	14.2	94.8	19.9
84	Difenoconazole	0.003	0.008	1.0000	305.5	65.4	1.5	92.2	7.1	73.9	7.7	118.0	0.8	101.3	19.6	91.5	21.3
85	Diflufenican	0.002	0.007	1.0000	122.0	62.4	7.1	75.1	2.4	93.7	7.9	97.2	24.8	92.9	20.7	93.8	24.7
86	Dimepiperate	0.002	0.007	1.0000	47.5	72.6	3.6	73.5	3.8	72.4	0.8	92.3	11.4	87.6	5.9	91.9	13.9
87	Dimethachlor	0.002	0.007	0.9999	23.3	86.4	3.6	85.9	4.4	80.2	0.8	101.6	4.8	95.3	5.6	94.7	8.3
88	Dimethametryn	0.001	0.003	1.0000	42.4	77.1	3.4	77.9	3.0	76.4	0.9	95.8	10.3	91.2	4.5	92.7	12.0
89	Dimethenamid	0.002	0.007	0.9999	25.3	82.1	3.5	83.6	3.5	80.5	1.2	98.3	6.4	93.8	5.8	94.5	9.2
90	Dimethipin	0.003	0.009	0.9954	-31.9	82.2	11.0	74.2	11.3	90.3	13.6	106.5	14.3	92.6	10.6	94.6	28.7
91	Dimethomorph (*E*)	0.003	0.008	1.0000	136.2	30.7	20.7	94.2	6.1	76.4	8.9	71.1	55.2	91.5	40.4	98.0	37.9
	Dimethomorph (*Z*)	0.003	0.009	0.9992	137.3	23.7	28.7	93.2	8.1	82.3	9.0	85.9	61.1	93.7	45.5	101.9	38.0
92	Dimethylvinphos (*E*)	0.002	0.007	0.9996	52.2	73.7	12.7	74.8	15.8	70.8	6.0	99.4	15.2	92.7	10.2	96.7	16.6
	Dimethylvinphos (*Z*)	0.002	0.007	0.9996	89.8	81.4	11.8	77.4	14.7	76.5	11.8	102.9	10.6	95.7	11.8	96.3	17.4
93	Diniconazole	0.002	0.006	0.9996	166.9	95.3	9.2	93.2	2.7	74.3	6.9	94.7	30.9	91.5	23.1	82.8	14.6
94	Dinitramine	0.002	0.007	0.9999	55.9	82.8	5.0	80.8	2.8	76.6	1.2	94.9	8.1	85.1	5.0	95.8	16.1
95	Dioxathion	0.002	0.007	1.0000	37.5	87.5	1.6	87.0	3.6	78.6	2.1	95.4	2.8	91.0	10.8	95.6	8.1
96	Diphenamid	0.002	0.007	1.0000	24.8	84.5	2.9	83.6	2.5	79.8	1.0	98.6	6.3	93.0	0.9	93.9	10.1
97	Diphenylamine	0.003	0.008	0.9985	48.7	79.7	3.1	80.7	4.5	76.2	3.6	92.9	4.1	90.5	10.3	90.1	5.4
98	Dithiopyr	0.002	0.007	0.9999	23.2	79.0	4.4	82.5	3.0	79.6	0.7	95.3	7.9	93.8	5.0	93.3	10.7
99	Edifenphos	0.001	0.004	0.9986	54.6	67.4	12.7	88.8	4.5	83.1	9.1	98.1	23.0	100.0	6.9	105.6	26.8
100	Endosulfan, α-	0.003	0.008	0.9992	15.3	85.6	3.5	90.4	3.2	83.4	1.3	95.6	1.0	92.0	8.1	94.8	5.2
	Endosulfan, β-	0.002	0.007	0.9998	3.7	92.9	2.9	93.6	6.0	88.1	3.7	97.3	11.3	98.1	5.5	97.3	5.1

No.	Analyte	LOD	LOQ	R^2	ME.(%)	Repeatability (n=5)						Reproducibility (n=11)					
						0.01 mg kg^{-1}		0.02 mg kg^{-1}		0.1 mg kg^{-1}		0.01 mg kg^{-1}		0.02 mg kg^{-1}		0.1 mg kg^{-1}	
						Rec. (%)	CV (%)	Rec. (%)	CV (%)	Rec. (%)	CV (%)	Rec. (%)	CV (%)	Rec. (%)	CV (%)	Rec. (%)	CV (%)
	Endosulfan, sulfate-	0.002	0.006	1.0000	11.5	78.9	2.7	80.9	2.8	76.4	0.6	95.0	20.6	93.0	7.9	100.2	15.9
101	Endrin	0.002	0.006	0.9999	18.1	100.3	1.8	107.0	5.2	105.2	2.3	100.3	9.6	101.2	15.5	100.8	15.4
	δ-keto-Endrin	0.002	0.007	0.9999	18.9	79.4	4.3	79.8	4.1	75.6	4.7	89.0	9.9	88.0	12.8	89.3	9.4
102	EPN	0.003	0.008	0.9992	46.2	76.1	6.7	72.2	5.1	79.5	12.4	95.3	14.3	91.7	11.9	84.7	13.7
103	Epoxiconazole	0.002	0.007	0.9999	56.4	85.0	4.5	86.0	2.9	86.6	1.8	103.5	7.7	102.0	6.7	96.3	12.0
104	EPTC	0.003	0.009	0.9972	10.6	76.1	2.0	87.4	5.9	81.2	6.3	88.7	8.7	89.5	8.6	92.6	7.0
105	Etaconazole-1	0.002	0.005	1.0000	45.6	68.8	16.7	77.3	2.3	77.0	1.4	91.6	16.3	92.9	6.5	95.8	10.4
	Etaconazole-2	0.003	0.008	0.9999	28.6	85.2	4.6	81.3	6.1	79.5	1.1	100.4	8.2	93.3	4.5	96.9	12.6
106	Ethalfluralin	0.003	0.008	0.9997	43.8	80.2	4.0	81.3	4.5	77.0	3.5	92.6	5.2	87.6	4.7	93.1	12.7
107	Ethion	0.002	0.007	0.9997	47.3	68.3	7.7	93.0	2.1	76.1	7.7	92.4	15.6	89.0	11.8	95.4	21.0
108	Ethofumesate	0.002	0.007	1.0000	31.3	81.1	3.4	82.1	3.0	78.9	0.8	97.4	8.1	93.7	4.1	93.9	10.2
109	Ethoprophos	0.002	0.007	0.9989	87.2	70.2	6.4	75.6	5.0	73.2	3.5	88.4	14.1	86.2	2.2	87.4	13.7
110	Ethychlozate	0.002	0.007	0.9998	127.5	74.3	5.4	93.4	3.2	75.0	5.6	96.3	15.0	87.8	10.2	93.5	24.5
111	Etoxazole	0.002	0.007	0.9996	63.6	76.4	4.5	73.0	3.9	74.3	1.3	98.1	11.8	92.9	11.2	94.9	16.7
112	Etridiazole	0.003	0.009	0.9966	33.4	80.9	7.1	93.8	7.3	87.8	12.6	92.9	2.8	93.2	11.3	98.3	2.2
113	Fenamidone	0.002	0.007	0.9992	47.2	80.0	3.9	76.9	2.6	77.6	0.9	103.5	12.2	96.3	9.8	98.4	16.3
114	Fenarimol	0.002	0.007	0.9996	78.8	68.7	5.0	96.4	3.4	71.1	1.6	93.9	19.6	90.0	17.7	92.4	25.7
115	Fenbuconazole	0.002	0.007	0.9983	95.7	103.4	5.9	93.4	5.4	76.8	6.1	91.0	25.3	89.4	23.0	91.1	27.0
116	Fenchlorphos	0.002	0.007	0.9999	34.3	71.3	5.9	74.7	5.7	72.4	2.3	88.0	8.9	86.2	6.4	88.2	8.5
117	Fenclorim	0.003	0.008	0.9986	38.9	83.3	2.6	87.2	3.6	77.7	3.7	94.8	6.4	90.8	12.0	88.4	2.1
118	Fenfuram	0.002	0.007	1.0000	48.6	77.7	3.0	79.8	1.6	77.1	1.7	96.6	9.5	88.3	1.5	91.9	11.2
119	Fenitrothion	0.003	0.008	0.9997	83.5	72.4	7.8	89.5	2.5	78.2	9.3	93.1	11.9	84.1	8.3	91.9	23.9
120	Fenobucarb	0.003	0.008	0.9979	57.5	77.2	9.3	76.8	10.1	92.3	17.5	94.2	11.8	86.7	3.5	85.9	16.1

No.	Analyte	LOD	LOQ	R^2	ME.(%)	Repeatability (n=5)						Reproducibility (n=11)					
						0.01 mg kg^{-1}		0.02 mg kg^{-1}		0.1 mg kg^{-1}		0.01 mg kg^{-1}		0.02 mg kg^{-1}		0.1 mg kg^{-1}	
						Rec. (%)	CV (%)	Rec. (%)	CV (%)	Rec. (%)	CV (%)	Rec. (%)	CV (%)	Rec. (%)	CV (%)	Rec. (%)	CV (%)
121	Fenothiocarb	0.002	0.006	0.9994	93.7	74.3	3.1	75.3	3.4	74.8	0.8	91.3	15.0	83.5	13.8	87.1	20.5
122	Fenoxanil	0.002	0.007	0.9996	176.2	81.0	6.8	80.6	5.1	70.2	2.9	100.5	13.3	92.9	8.3	92.0	20.4
123	Fenpropathrin	0.002	0.007	0.9989	46.3	81.5	4.5	75.1	3.5	74.7	0.9	98.3	7.3	91.6	7.9	96.3	17.1
124	Fenpropimorph	0.002	0.007	1.0000	40.0	81.3	2.4	82.3	2.4	78.6	1.4	93.5	4.0	87.6	5.2	89.6	12.2
125	Fenpyrazamine	0.002	0.007	0.9999	99.2	90.8	4.6	90.7	5.5	78.9	5.7	80.4	26.9	95.2	25.1	82.5	19.0
126	Fenson	0.002	0.007	0.9999	12.8	85.9	3.9	86.7	3.9	79.5	1.0	101.6	5.6	98.6	8.5	96.8	7.6
127	Fenthion	0.003	0.008	0.9998	37.5	75.0	6.5	74.3	5.6	70.9	1.9	93.8	9.8	88.8	7.2	92.0	13.1
128	Fenvalerate-1	0.003	0.008	0.9998	51.7	59.7	7.2	62.6	6.7	65.8	3.3	92.9	26.6	90.0	20.4	99.0	32.2
	Fenvalerate-2	0.003	0.008	0.9997	128.5	58.4	6.8	61.3	5.2	61.4	4.0	93.3	27.7	90.9	21.6	99.2	33.0
129	Fipronil	0.002	0.007	0.9998	116.9	75.8	5.8	77.2	4.0	76.4	1.5	99.4	17.2	97.2	10.2	87.9	7.7
130	Flamprop-isopropyl	0.003	0.008	1.0000	29.8	78.7	4.2	81.8	3.0	79.3	0.8	97.0	11.7	96.2	4.8	97.8	10.8
131	Fluacrypyrim	0.003	0.008	0.9998	72.3	75.0	5.5	74.3	3.5	73.2	0.9	97.3	14.1	93.0	9.9	95.1	18.5
132	Fluazifop-butyl	0.002	0.007	0.9998	69.3	77.7	5.1	78.4	4.1	76.5	0.8	90.7	16.3	86.9	10.3	94.2	14.5
133	Fluchloralin	0.002	0.006	0.9999	28.0	83.6	5.5	80.0	5.8	74.4	2.1	95.1	3.6	86.1	3.7	92.1	16.2
134	Flucythrinate-1	0.003	0.008	1.0000	91.5	60.6	9.5	90.7	5.4	94.5	10.5	94.1	25.9	92.0	21.3	80.0	14.2
	Flucythrinate-2	0.003	0.009	1.0000	99.7	63.4	6.5	92.7	5.5	75.9	10.0	97.0	24.2	91.5	20.7	79.4	13.3
135	Fluensulfone	0.003	0.008	0.9994	75.8	83.1	3.6	85.4	5.5	79.7	2.5	99.6	7.2	95.3	11.6	97.3	7.8
136	Flufenpyr-ethyl	0.003	0.008	0.9994	54.8	61.3	7.2	70.6	2.5	70.5	3.0	94.5	26.1	90.4	17.3	98.4	21.9
137	Flumetralin	0.002	0.006	0.9995	36.6	82.3	4.8	77.3	3.8	71.0	0.9	95.2	7.9	91.9	6.2	97.9	19.6
138	Flumioxazin	0.003	0.009	1.0000	80.5	98.6	5.6	93.6	6.7	77.6	10.0	93.9	22.5	100.1	8.3	89.4	27.3
139	Fluopyram	0.002	0.007	0.9994	86.9	81.2	5.0	78.4	3.5	76.8	1.3	102.1	10.5	95.2	7.9	97.7	16.2
140	Fluorochloridone	0.002	0.007	0.9999	25.1	109.9	4.6	102.8	9.1	96.9	3.0	112.9	4.6	105.6	9.4	95.4	12.9
141	Fluquinconazole	0.002	0.007	1.0000	31.3	70.6	4.2	71.6	4.2	72.2	1.3	99.9	20.1	97.9	16.6	87.0	9.6

No.	Analyte	LOD	LOQ	R^2	ME.(%)	Repeatability (n=5)						Reproducibility (n=11)					
						0.01 mg kg^{-1}		0.02 mg kg^{-1}		0.1 mg kg^{-1}		0.01 mg kg^{-1}		0.02 mg kg^{-1}		0.1 mg kg^{-1}	
						Rec. (%)	CV (%)	Rec. (%)	CV (%)	Rec. (%)	CV (%)	Rec. (%)	CV (%)	Rec. (%)	CV (%)	Rec. (%)	CV (%)
142	Flusilazole	0.001	0.004	1.0000	44.3	79.1	3.8	78.6	3.2	76.7	0.6	98.4	10.4	94.9	7.2	97.0	13.3
143	Flutianil	0.003	0.008	0.9997	81.1	70.2	4.6	71.0	2.3	71.7	1.5	101.8	20.7	99.6	18.6	89.1	11.6
144	Fluvalinate-1	0.003	0.009	0.9977	51.3	25.9	29.7	44.6	8.9	53.4	6.9	112.6	18.6	97.2	31.3	107.4	48.0
	Fluvalinate-2	0.003	0.009	0.9989	43.0	35.0	19.1	46.7	14.0	55.8	7.4	113.9	17.0	96.1	28.8	106.6	47.7
145	Fluxapyroxad	0.003	0.008	0.9995	145.5	69.8	6.0	97.0	3.4	74.3	5.7	100.5	20.1	94.7	16.7	85.8	12.0
146	Fonofos	0.002	0.007	0.9996	33.0	81.4	3.7	84.8	2.4	80.6	2.4	96.5	5.6	91.6	5.9	91.6	6.7
147	Formothion	0.003	0.008	0.9993	47.8	62.7	14.7	80.9	5.9	74.5	11.8	79.9	11.1	78.7	16.2	96.7	16.1
148	Fthalide	0.002	0.007	1.0000	20.3	81.9	3.9	80.2	3.3	74.9	0.7	98.3	7.0	96.1	10.6	93.3	10.0
149	Halfenprox	0.002	0.007	0.9994	65.4	81.6	5.1	82.0	3.6	79.1	2.0	92.0	7.7	93.3	5.7	103.3	25.4
150	Heptachlor	0.002	0.006	0.9999	31.5	96.3	1.2	105.1	7.9	96.6	4.9	98.6	8.2	98.9	17.5	97.2	9.3
	Heptachlor epoxide	0.002	0.007	0.9998	11.5	92.1	1.5	96.1	3.5	89.1	1.2	102.9	7.9	97.8	11.1	94.2	7.6
151	Heptenophos	0.003	0.008	0.9981	71.7	65.6	9.0	73.9	10.2	71.4	4.9	84.8	18.4	81.1	8.3	86.5	13.3
152	Hexythiazox	0.002	0.006	0.9996	107.1	67.0	6.6	106.2	9.0	79.2	6.2	90.9	15.8	85.5	10.7	91.1	17.3
153	Indanofan	0.002	0.007	1.0000	73.2	72.7	6.4	76.1	6.3	75.3	4.9	98.9	15.9	96.9	11.7	102.6	16.2
154	Indoxacarb	0.002	0.007	0.9974	31.8	75.0	6.9	74.9	3.9	71.2	3.9	100.1	18.5	97.3	19.1	87.7	8.5
155	Ipconazole	0.002	0.007	0.9996	123.1	68.5	7.3	94.7	5.0	74.6	4.6	98.0	21.6	93.7	23.4	80.0	11.3
156	Iprobenfos	0.003	0.008	0.9998	136.9	68.1	6.5	96.2	3.3	82.0	9.8	94.8	18.2	89.6	12.4	94.9	19.5
157	Iprodione	0.003	0.009	0.9974	44.8	58.1	15.1	65.5	8.1	66.7	7.5	101.5	38.6	94.7	27.4	99.8	37.0
158	Isazofos	0.002	0.007	0.9999	28.7	84.0	3.4	83.0	4.4	79.6	1.4	97.3	5.4	90.4	2.3	92.7	11.2
159	Isofenphos	0.002	0.007	1.0000	36.1	76.2	3.3	77.3	3.4	75.8	0.9	96.5	11.4	92.1	6.5	95.0	13.8
160	Isofenphos-methyl	0.003	0.008	1.0000	36.9	78.9	3.6	77.9	3.3	75.8	0.9	98.7	9.9	93.0	7.0	94.5	13.6
161	Isoprocarb	0.003	0.008	0.9958	58.6	65.9	9.8	71.5	10.7	99.7	19.5	91.6	18.7	85.6	6.5	86.3	17.9
162	Isopropalin	0.002	0.007	0.9998	39.5	82.8	3.0	79.0	2.8	75.3	1.5	95.2	2.9	87.6	3.5	95.1	14.2

No.	Analyte	LOD	LOQ	R^2	ME.(%)	Repeatability (*n*=5)						Reproducibility (*n*=11)					
						0.01 mg kg^{-1}		0.02 mg kg^{-1}		0.1 mg kg^{-1}		0.01 mg kg^{-1}		0.02 mg kg^{-1}		0.1 mg kg^{-1}	
						Rec. (%)	CV (%)	Rec. (%)	CV (%)	Rec. (%)	CV (%)	Rec. (%)	CV (%)	Rec. (%)	CV (%)	Rec. (%)	CV (%)
163	Isoprothiolane	0.002	0.006	0.9999	43.7	79.4	4.1	81.4	3.5	78.2	0.8	99.8	10.1	95.6	5.7	96.1	11.3
164	Isopyrazam	0.003	0.008	0.9988	143.7	69.1	5.0	100.3	3.2	77.4	7.7	99.2	20.9	94.9	22.8	83.9	12.4
165	Isotianil	0.003	0.008	0.9993	272.5	68.8	5.4	102.4	3.0	73.3	6.6	98.9	21.2	90.9	16.2	93.8	25.4
166	Isoxadifen-ethyl	0.002	0.007	1.0000	41.9	73.8	4.6	73.2	6.1	72.7	1.9	86.3	17.3	85.1	7.1	85.5	4.8
167	Kresoxim-methyl	0.002	0.007	1.0000	25.6	83.6	3.5	84.2	3.4	80.3	0.5	98.3	7.7	96.1	2.3	98.0	11.6
168	Leptophos	0.002	0.007	0.9998	34.4	73.6	6.6	70.9	6.8	76.6	8.8	95.3	12.9	96.4	20.0	97.3	20.2
169	Mefenpyr-diethyl	0.002	0.007	0.9998	61.7	74.8	4.4	72.8	4.1	71.6	1.6	88.3	24.5	86.2	17.4	93.3	24.0
170	Mepanipyrim	0.001	0.003	0.9995	84.5	79.4	4.6	80.4	3.4	76.4	0.6	101.8	11.7	96.0	8.7	96.9	11.4
171	Mepronil	0.002	0.007	0.9998	129.6	68.6	5.5	95.0	3.7	73.7	6.7	101.6	21.6	95.6	17.7	101.3	20.6
172	Metalaxyl	0.002	0.007	1.0000	31.8	77.0	3.4	78.9	3.5	76.6	1.1	95.1	10.4	89.9	2.3	91.0	11.5
173	Methidathion	0.003	0.008	0.9999	53.0	75.1	12.7	75.0	14.6	77.7	10.5	92.3	8.4	85.0	2.2	88.7	17.0
174	Methoprotryn	0.002	0.007	0.9999	63.0	77.9	4.5	78.3	3.9	76.9	0.9	97.2	10.8	96.0	9.3	99.8	12.9
175	Methoxychlor	0.002	0.005	0.9989	50.6	84.0	10.7	96.2	3.3	88.5	8.5	95.1	1.9	98.0	11.5	106.3	15.8
176	Methyl trithion	0.002	0.007	0.9999	37.8	65.3	11.8	82.5	3.7	74.7	7.8	87.9	15.4	82.3	10.6	87.6	20.8
177	Metolachlor	0.002	0.007	0.9993	42.2	82.1	3.1	84.1	2.9	79.2	0.8	98.1	6.5	93.3	2.3	93.5	9.2
178	Metribuzin	0.002	0.006	0.9998	45.8	79.4	2.7	78.9	3.4	75.7	1.8	96.1	7.9	88.9	1.4	93.1	15.0
179	MGK-264	0.002	0.007	0.9999	30.3	77.8	3.3	80.3	3.2	77.1	1.5	91.2	7.3	92.0	4.2	93.3	9.5
180	Molinate	0.002	0.007	0.9970	17.3	81.3	2.6	89.6	4.1	81.1	4.7	107.3	20.3	92.5	7.8	91.9	4.4
181	Monolinuron	0.002	0.007	0.9997	89.3	75.4	11.8	82.3	6.9	75.8	5.7	92.7	11.3	89.2	2.8	91.3	14.2
182	Myclobutanil	0.002	0.005	1.0000	45.3	79.3	3.1	79.7	2.0	77.6	0.5	101.0	11.2	95.9	6.8	96.8	13.1
183	Nitrothal-isopropyl	0.002	0.007	0.9995	59.7	85.8	5.3	78.1	3.5	82.7	6.9	96.7	5.7	86.5	1.6	83.6	6.4
184	Nonachlor-*cis*	0.002	0.007	1.0000	7.1	93.5	2.2	95.3	2.4	90.2	1.0	98.5	4.9	97.6	8.7	98.9	1.3
	Nonachlor-*trans*	0.002	0.006	0.9992	14.6	92.9	4.4	98.1	2.0	92.2	0.9	93.4	9.8	94.6	13.3	94.6	8.6

No.	Analyte	LOD	LOQ	R^2	ME.(%)	Repeatability (*n*=5)						Reproducibility (*n*=11)					
						0.01 mg kg^{-1}		0.02 mg kg^{-1}		0.1 mg kg^{-1}		0.01 mg kg^{-1}		0.02 mg kg^{-1}		0.1 mg kg^{-1}	
						Rec. (%)	CV (%)	Rec. (%)	CV (%)	Rec. (%)	CV (%)	Rec. (%)	CV (%)	Rec. (%)	CV (%)	Rec. (%)	CV (%)
185	Nuarimol	0.002	0.007	0.9999	109.5	64.0	8.1	72.1	5.0	72.4	6.6	93.6	21.6	89.2	17.1	90.9	22.9
186	Ortho-phenyl phenol	0.003	0.008	0.9989	132.6	76.8	2.1	81.4	5.1	77.6	2.4	101.5	18.5	93.2	4.6	91.2	7.0
187	Oxadiazon	0.002	0.007	0.9998	35.1	80.5	3.8	82.8	2.7	78.6	0.7	96.9	7.6	94.0	2.0	93.9	9.7
188	Oxadixyl	0.002	0.007	0.9999	16.7	83.1	3.9	81.9	3.2	77.4	1.1	100.6	8.1	96.6	5.1	97.9	13.0
189	Oxyfluorfen	0.002	0.005	0.9999	79.0	77.8	8.9	77.9	4.9	73.0	1.3	95.0	9.1	89.6	3.3	86.9	9.9
190	Paclobutrazol	0.002	0.006	0.9990	150.8	102.5	5.1	93.3	2.8	73.6	4.2	91.1	28.7	88.5	18.1	93.6	25.2
191	Parathion	0.002	0.007	0.9999	67.8	80.2	7.4	75.9	6.3	70.1	1.1	98.0	7.9	88.8	5.4	85.2	8.4
192	Parathion-methyl	0.003	0.008	1.0000	38.4	68.4	10.4	90.0	4.1	85.2	9.4	95.5	18.7	88.7	18.4	97.2	24.3
193	Penconazole	0.002	0.007	1.0000	35.3	78.4	2.7	79.4	3.0	76.2	0.7	98.0	9.7	94.1	6.8	93.8	12.1
194	Pendimethalin	0.002	0.006	0.9996	58.3	81.6	2.8	77.6	2.8	73.9	2.0	93.4	4.2	86.4	2.5	96.1	17.0
195	Penflufen	0.002	0.007	0.9998	90.6	69.3	5.9	71.1	3.8	72.0	1.2	95.9	18.8	92.3	12.7	94.3	18.2
196	Pentachlorobenzonitrile	0.002	0.007	0.9993	32.0	81.0	3.6	85.5	3.1	79.0	2.5	95.2	7.5	91.4	11.2	89.6	1.8
197	Penthiopyrad	0.002	0.007	0.9991	117.1	68.1	6.2	98.7	2.8	72.9	2.3	96.8	19.8	91.7	13.4	96.2	20.4
198	Pentoxazone	0.003	0.008	0.9998	50.9	80.2	3.6	78.1	3.8	76.9	1.3	100.7	11.6	96.3	9.4	96.2	15.2
199	Permethrin-cis	0.002	0.007	0.9997	78.9	73.2	19.9	74.1	13.0	74.8	7.6	95.2	13.7	96.1	12.6	95.3	22.3
	Permethrin-trans	0.003	0.008	0.9992	77.6	70.6	8.1	71.4	1.5	78.8	10.8	82.5	27.6	92.4	16.3	91.7	25.2
200	Perthane	0.002	0.006	1.0000	25.4	82.8	2.7	85.0	2.7	81.0	0.5	95.1	3.1	93.0	1.8	93.1	8.9
201	Phenthoate	0.002	0.007	1.0000	35.3	77.3	8.0	75.8	6.3	72.2	2.0	96.1	9.2	90.4	7.2	96.0	20.4
202	Phosphamidon (*E*)	0.002	0.007	0.9988	133.6	78.0	28.4	95.5	17.9	87.6	14.9	98.9	11.3	93.2	19.8	98.8	18.8
	Phosphamidon (*Z*)	0.002	0.007	0.9993	54.2	90.7	14.0	92.5	8.9	86.8	13.3	103.1	14.4	99.3	13.9	85.1	19.0
203	Phosalone	0.003	0.008	1.0000	46.4	67.9	8.2	95.7	5.3	81.3	13.6	90.9	15.6	92.2	15.0	89.3	17.9
204	Phosmet	0.003	0.009	0.9999	-19.0	78.4	14.9	95.0	6.3	85.5	13.4	99.2	12.7	96.5	17.5	101.5	22.9
205	Picoxystrobin	0.002	0.007	0.9990	79.2	80.1	4.6	81.8	3.2	77.6	0.8	100.1	10.0	95.3	4.3	94.7	13.0

No.	Analyte	LOD	LOQ	R^2	ME.(%)	Repeatability (*n*=5)						Reproducibility (*n*=11)					
						0.01 mg kg^{-1}		0.02 mg kg^{-1}		0.1 mg kg^{-1}		0.01 mg kg^{-1}		0.02 mg kg^{-1}		0.1 mg kg^{-1}	
						Rec. (%)	CV (%)	Rec. (%)	CV (%)	Rec. (%)	CV (%)	Rec. (%)	CV (%)	Rec. (%)	CV (%)	Rec. (%)	CV (%)
206	Piperonyl butoxide	0.002	0.007	0.9998	127.0	87.4	4.8	81.7	3.6	80.3	1.9	104.8	6.9	99.0	7.3	101.1	14.2
207	Pirimicarb	0.002	0.007	0.9999	45.3	76.2	3.6	78.8	2.6	75.0	2.1	94.7	9.4	91.5	6.5	92.2	10.0
208	Pirimiphos-ethyl	0.003	0.008	1.0000	38.2	80.5	3.0	79.6	3.4	76.8	1.2	97.2	7.1	90.3	3.1	94.9	10.0
209	Pirimiphos-methyl	0.002	0.007	0.9999	40.8	75.5	4.6	76.6	4.3	74.2	1.4	91.0	9.6	90.5	7.2	90.9	11.2
210	Pretilachlor	0.001	0.003	0.9997	49.0	85.6	5.1	85.3	5.4	78.7	1.4	103.4	7.1	94.9	0.3	98.7	10.1
211	Prochloraz	0.002	0.007	0.9994	135.6	53.1	11.0	51.1	9.1	55.4	2.5	82.3	24.4	84.6	29.0	102.3	40.5
	2,4,6-trichlorophenol	0.002	0.007	0.9997	61.4	66.6	3.3	73.3	1.3	71.2	2.6	75.4	26.1	73.4	28.2	67.0	33.5
212	Procymidone	0.002	0.007	1.0000	20.2	81.8	3.7	83.4	2.7	79.3	0.4	98.6	6.9	95.7	5.1	94.5	9.1
213	Prodiamine	0.002	0.006	0.9996	49.3	82.0	2.3	79.9	3.5	76.8	1.1	94.8	5.1	89.9	3.6	97.3	16.8
214	Profenofos	0.001	0.002	0.9987	97.4	68.2	13.2	80.9	3.5	75.4	11.0	96.8	18.9	90.2	15.5	94.6	22.4
215	Profluralin	0.002	0.007	1.0000	35.1	88.6	3.3	85.1	3.0	79.3	3.2	94.5	6.3	91.4	6.6	96.6	10.4
216	Prohydrojasmon	0.002	0.007	0.9999	58.0	73.2	4.5	76.7	1.9	76.5	2.4	80.5	25.0	83.1	6.7	89.0	12.0
217	Prometon	0.003	0.008	0.9999	69.2	80.9	2.7	85.1	2.0	78.6	1.1	93.9	7.3	92.9	6.6	93.0	6.3
218	Prometryn	0.002	0.007	0.9999	38.5	76.0	3.0	78.6	2.3	77.1	0.9	95.9	10.3	90.2	5.0	90.8	10.4
219	Propachlor	0.003	0.008	0.9987	37.5	78.2	6.6	83.9	6.0	78.4	3.0	96.3	8.4	92.5	6.2	92.5	7.3
220	Propanil	0.002	0.007	0.9997	76.5	80.6	5.4	78.0	3.4	75.8	2.1	98.4	8.7	90.2	3.4	92.4	12.3
221	Propazine	0.002	0.006	0.9996	45.7	84.6	3.4	86.2	3.4	79.6	1.7	98.2	3.7	92.5	6.9	92.6	6.0
222	Propetamphos	0.002	0.007	0.9999	59.3	81.4	3.1	81.1	2.7	76.7	1.8	96.8	6.6	90.4	2.0	92.5	12.1
223	Propham	0.003	0.009	0.9978	84.0	76.5	3.3	84.4	3.2	79.3	3.8	96.1	10.0	93.4	7.1	92.3	5.0
224	Propiconazole-1	0.002	0.007	1.0000	53.0	69.8	5.5	72.7	4.6	71.8	1.8	91.9	14.2	87.8	13.1	90.9	20.4
	Propiconazole-2	0.002	0.007	1.0000	67.4	78.6	3.7	77.6	4.3	74.8	2.5	95.2	8.4	90.1	10.7	91.7	19.5
225	Propisochlor	0.003	0.009	0.9999	28.5	85.5	4.2	85.3	5.2	80.4	1.4	100.0	4.6	92.3	3.1	93.3	10.1
226	Propyzamide	0.002	0.007	0.9999	53.6	80.5	6.6	80.5	5.9	76.8	1.3	96.6	6.4	90.9	5.3	94.5	10.3

No.	Analyte	LOD	LOQ	R^2	ME.(%)	Repeatability (n=5)						Reproducibility (n=11)					
						0.01 mg kg^{-1}		0.02 mg kg^{-1}		0.1 mg kg^{-1}		0.01 mg kg^{-1}		0.02 mg kg^{-1}		0.1 mg kg^{-1}	
						Rec. (%)	CV (%)	Rec. (%)	CV (%)	Rec. (%)	CV (%)	Rec. (%)	CV (%)	Rec. (%)	CV (%)	Rec. (%)	CV (%)
227	Prothiofos	0.001	0.002	0.9996	59.4	77.3	4.2	78.5	3.5	75.6	1.1	94.9	8.9	92.4	8.3	90.7	7.5
228	Pyracarbolid	0.002	0.006	0.9999	79.7	79.3	3.7	75.1	2.3	73.0	1.4	98.3	11.0	88.2	6.8	92.4	17.2
229	Pyraclofos	0.003	0.008	0.9985	86.8	80.1	8.4	94.5	9.8	85.7	17.1	91.7	29.7	89.9	15.6	79.3	5.2
230	Pyraflufen-ethyl	0.003	0.008	1.0000	74.6	63.2	2.0	70.8	12.0	72.7	3.8	85.1	28.8	81.8	20.3	92.1	23.4
231	Pyrazophos	0.003	0.008	0.9994	35.8	66.0	11.4	98.2	4.5	77.1	9.8	94.3	19.7	93.3	19.7	88.0	17.1
232	Pyridalyl	0.003	0.008	0.9998	100.1	69.8	4.9	98.3	3.8	82.1	7.0	94.5	15.6	94.9	16.7	97.4	22.5
233	Pyrifenox	0.002	0.007	0.9999	45.2	76.5	5.0	77.9	3.9	77.7	0.4	95.8	14.0	90.0	4.8	93.6	16.9
234	Pyriftalid	0.003	0.008	0.9992	96.5	78.3	5.8	72.7	9.2	71.8	3.3	100.8	13.8	93.3	14.7	83.5	3.9
235	Pyrimethanil	0.002	0.006	0.9994	47.7	80.5	3.2	83.0	5.0	79.5	2.0	98.2	8.5	94.8	10.8	96.5	7.9
236	Pyriminobac-methyl (*E*)	0.003	0.008	0.9998	64.1	79.6	5.8	80.0	4.2	79.4	0.7	100.2	10.5	95.4	5.9	89.5	3.9
	Pyriminobac-methyl (*Z*)	0.002	0.007	0.9999	36.8	80.3	4.1	82.0	3.7	79.6	0.5	100.9	10.2	97.8	7.0	91.1	5.4
237	Quinalphos	0.002	0.007	1.0000	33.1	75.5	6.7	74.8	6.2	71.6	1.3	94.7	10.1	88.2	5.3	90.6	15.9
238	Quinoxyfen	0.002	0.007	1.0000	22.3	77.5	2.8	79.7	2.2	76.3	0.9	97.7	11.2	93.5	5.6	93.1	8.0
239	Quintozene	0.002	0.007	0.9996	34.1	88.1	3.2	87.2	5.5	74.5	4.6	87.4	10.8	85.0	14.4	80.5	4.9
240	Quizalofop-ethyl	0.003	0.008	0.9993	96.9	59.6	7.4	59.3	5.8	60.0	3.7	44.2	58.2	39.0	88.2	54.3	34.2
241	Silafluofen	0.003	0.008	0.9993	73.6	76.9	4.7	73.2	2.7	70.6	2.2	95.1	9.1	93.7	12.5	90.3	13.7
242	Simeconazole	0.002	0.007	0.9998	70.2	71.8	3.4	72.9	2.9	73.3	1.2	94.8	15.4	87.9	7.4	93.8	16.2
243	Simetryn	0.002	0.007	1.0000	48.0	81.2	2.7	80.3	2.9	77.8	1.6	96.4	7.2	87.6	2.6	90.2	11.7
244	Spiromesifen	0.002	0.005	1.0000	35.0	76.5	12.0	71.4	10.7	74.1	7.3	96.8	13.2	93.6	13.2	91.4	20.6
245	Spiroxamine-1	0.003	0.009	0.9999	74.9	67.6	4.2	72.9	1.8	71.9	3.0	93.2	21.6	85.6	11.3	87.8	13.1
	Spiroxamine-2	0.003	0.008	0.9998	56.8	76.8	2.5	78.9	2.3	76.0	2.2	98.3	15.4	87.5	10.6	89.0	11.6
246	Sulfotep	0.003	0.008	0.9992	40.4	77.0	3.1	81.9	3.8	77.8	2.5	95.2	8.8	92.4	7.6	92.6	8.4
247	Tebuconazole	0.002	0.007	1.0000	166.5	86.7	6.9	74.6	2.4	77.3	6.4	95.1	29.3	94.0	22.2	85.2	18.0

No.	Analyte	LOD	LOQ	R^2	ME.(%)	Repeatability (*n*=5)						Reproducibility (*n*=11)					
						0.01 mg kg^{-1}		0.02 mg kg^{-1}		0.1 mg kg^{-1}		0.01 mg kg^{-1}		0.02 mg kg^{-1}		0.1 mg kg^{-1}	
						Rec. (%)	CV (%)	Rec. (%)	CV (%)	Rec. (%)	CV (%)	Rec. (%)	CV (%)	Rec. (%)	CV (%)	Rec. (%)	CV (%)
248	Tebufenpyrad	0.003	0.008	0.9995	65.3	77.5	5.0	73.7	3.2	73.9	0.9	97.2	12.1	94.6	11.7	94.6	15.9
249	Tebupirimfos	0.003	0.008	0.9998	33.5	72.6	2.0	79.1	3.4	77.6	2.5	91.8	10.6	89.7	4.8	91.4	8.8
250	Tecnazene	0.003	0.008	0.9990	33.7	82.6	2.8	85.0	4.2	78.2	4.4	91.1	2.1	88.1	8.7	89.7	5.3
251	Tefluthrin	0.002	0.007	0.9998	22.7	80.4	2.0	85.3	2.4	81.4	2.1	94.1	4.4	90.8	5.2	91.7	6.0
252	Terbacil	0.003	0.008	0.9999	115.4	70.0	8.1	73.9	4.5	71.1	1.1	99.5	19.2	91.4	10.1	97.3	16.4
253	Terbumeton	0.002	0.007	0.9995	53.1	83.0	3.1	85.7	3.0	80.0	0.8	93.1	14.5	88.6	8.1	92.7	5.5
254	Terbutryn	0.002	0.006	1.0000	42.3	80.7	3.0	81.2	2.7	79.0	0.9	96.5	7.8	90.6	0.4	92.5	9.1
255	Tetrachlorvinphos	0.002	0.007	0.9999	51.2	74.5	13.2	88.0	5.7	82.8	13.2	98.7	14.5	95.9	7.2	94.1	22.5
256	Tetraconazole	0.002	0.007	1.0000	44.6	82.2	4.0	80.6	3.0	78.1	0.5	101.4	9.2	94.3	5.0	95.5	13.2
257	Tetradifon	0.002	0.007	1.0000	20.0	83.6	3.0	83.1	1.7	78.2	0.7	98.8	5.6	97.2	4.3	95.6	9.5
258	Tetramethrin-1	0.003	0.008	0.9998	116.1	63.7	17.4	87.2	2.2	73.3	7.9	108.3	34.8	87.1	17.0	92.2	27.4
	Tetramethrin-2	0.003	0.009	0.9998	82.9	66.3	17.2	78.0	6.3	62.0	1.2	95.2	2.7	103.1	9.6	116.0	0.0
259	Thifluzamide	0.002	0.007	1.0000	157.2	70.2	5.8	73.8	3.3	72.9	1.3	96.4	17.6	93.4	10.6	97.7	20.6
260	Thiometon	0.003	0.008	0.9988	56.1	75.8	5.0	77.7	5.3	73.3	2.9	89.9	11.9	84.1	4.8	86.5	15.5
261	Thionazin	0.003	0.008	0.9991	44.6	79.2	3.1	82.8	4.3	76.6	2.6	96.5	7.7	91.3	5.6	90.7	8.5
262	Tolclofos-methyl	0.002	0.007	0.9997	28.7	76.7	4.7	80.2	3.8	76.7	1.2	94.2	8.3	91.2	5.6	90.5	8.5
263	Triadimefon	0.002	0.007	1.0000	41.6	83.6	2.9	81.8	2.3	78.9	0.8	99.1	7.1	90.3	3.5	92.0	14.2
264	Triadimenol	0.002	0.006	0.9999	86.6	69.7	5.8	74.9	4.1	71.4	1.7	93.0	17.9	89.7	11.7	81.5	14.1
265	Tri-allate	0.002	0.007	0.9994	26.8	79.7	3.8	85.6	1.8	81.1	3.5	93.0	4.1	90.2	6.8	89.7	7.4
266	Triazophos	0.003	0.008	1.0000	61.9	73.6	14.9	73.6	11.3	70.2	3.2	97.8	14.2	85.5	10.4	98.0	21.2
267	Tridiphane	0.002	0.005	0.9999	29.3	76.0	10.0	96.2	14.5	107.6	6.7	92.1	7.7	93.0	14.0	106.4	12.0
268	Trifloxystrobin	0.002	0.006	0.9998	49.7	82.5	4.6	81.1	3.6	79.0	1.0	100.8	8.4	93.7	4.9	97.1	17.8
269	Triflumizole	0.002	0.006	1.0000	50.4	76.0	4.5	77.8	4.1	73.3	2.5	99.1	13.0	94.0	8.0	95.4	14.8

No.	Analyte	LOD	LOQ	R^2	ME.(%)	Repeatability (*n*=5)						Reproducibility (*n*=11)					
						0.01 mg kg^{-1}		0.02 mg kg^{-1}		0.1 mg kg^{-1}		0.01 mg kg^{-1}		0.02 mg kg^{-1}		0.1 mg kg^{-1}	
						Rec. (%)	CV (%)	Rec. (%)	CV (%)	Rec. (%)	CV (%)	Rec. (%)	CV (%)	Rec. (%)	CV (%)	Rec. (%)	CV (%)
270	Trifluralin	0.002	0.007	0.9998	51.9	82.2	2.3	84.0	3.5	78.9	3.0	93.2	4.2	89.1	6.9	93.7	13.4
271	Vinclozolin	0.002	0.007	0.9996	22.3	77.8	3.8	81.4	3.7	75.9	3.1	90.4	10.7	91.2	5.1	92.4	8.9
272	Zoxamide	0.003	0.008	0.9983	78.6	70.3	11.4	87.5	6.0	83.4	16.4	92.4	14.2	91.9	21.6	96.1	23.3

Table S5. Method validation parameter of 272 pesticides in mandarin.

No.	Analyte	LOD	LOQ	R^2	ME.(%)	Repeatability (*n*=5)						Reproducibility (*n*=11)					
						0.01 mg kg^{-1}		0.02 mg kg^{-1}		0.1 mg kg^{-1}		0.01 mg kg^{-1}		0.02 mg kg^{-1}		0.1 mg kg^{-1}	
						Rec. (%)	CV (%)	Rec. (%)	CV (%)	Rec. (%)	CV (%)	Rec. (%)	CV (%)	Rec. (%)	CV (%)	Rec. (%)	CV (%)
1	2,6-DIPN	0.002	0.006	0.9954	119.0	113.5	11.7	102.8	6.0	103.7	8.9	130.8	15.3	120.2	0.2	95.6	6.3
2	Acetochlor	0.001	0.003	0.9983	97.0	106.5	5.1	107.4	0.6	104.8	5.4	96.6	11.5	88.5	18.2	89.4	15.8
	EMA	0.001	0.003	0.9988	52.8	100.9	8.4	79.5	13.3	117.9	8.4	89.9	3.5	98.5	8.7	98.1	11.3
	HEMA	0.001	0.003	0.9985	>500	30.4	10.2	41.0	12.9	41.7	7.7	49.8	73.7	57.6	71.9	45.7	25.7
3	Acrinathrin	0.002	0.005	0.9990	>500	91.6	5.0	90.2	3.9	72.9	4.9	110.7	13.8	101.5	20.5	123.9	22.0
4	Alachlor	0.001	0.004	0.9988	92.9	107.4	5.1	116.6	2.4	110.1	3.7	92.7	11.0	91.1	18.6	87.8	14.2
5	Aldrin	0.001	0.003	0.9984	72.3	105.5	3.1	97.4	3.1	95.9	4.8	95.5	8.3	87.3	9.6	88.4	5.1
	Dieldrin	0.001	0.002	0.9996	48.1	114.6	1.9	112.4	1.8	117.0	2.9	95.5	8.3	92.3	11.3	89.6	8.3
6	Allidochlor	0.001	0.003	0.9990	95.1	99.3	6.8	99.0	3.2	115.7	7.7	95.7	13.4	94.3	7.4	96.3	5.7
7	Ametryn	0.001	0.002	0.9991	147.6	110.2	2.2	105.0	2.1	96.2	4.0	92.8	1.1	89.5	8.4	86.8	12.5
8	Anilofos	0.001	0.004	0.9991	334.9	106.4	5.6	92.2	5.2	80.2	2.0	96.7	8.5	91.9	11.8	91.9	7.0
9	Aramite	0.001	0.002	0.9998	176.7	111.8	2.7	106.0	3.4	77.9	3.7	95.8	14.3	91.7	20.7	90.3	10.3
10	Aspon	0.001	0.002	0.9990	138.3	107.5	4.9	108.1	2.8	98.8	4.7	92.1	13.2	94.4	12.5	92.3	6.1
11	Atrazine	0.001	0.002	0.9988	134.9	108.5	5.9	101.3	2.5	95.3	4.6	92.0	19.0	91.0	13.2	91.0	3.4
12	Azaconazole	0.001	0.002	0.9999	94.9	117.6	3.0	107.5	2.5	97.5	3.1	94.1	2.5	93.3	9.5	91.0	6.6
13	Benfluralin	0.001	0.004	0.9976	150.6	106.2	4.5	108.9	1.8	111.7	5.2	97.2	8.5	91.7	9.6	92.2	7.1
14	Benfuresate	0.001	0.002	0.9985	72.5	108.3	1.8	103.5	3.1	99.4	4.9	91.0	8.7	88.5	17.0	92.6	3.6
15	Benodanil	0.001	0.003	0.9999	247.8	61.7	0.5	101.6	2.6	73.6	2.2	101.9	2.3	96.0	11.4	93.3	5.9
16	Benzoylprop-ethyl	0.001	0.002	0.9999	111.1	102.9	2.2	100.1	2.8	85.6	2.1	99.6	1.5	97.6	6.0	93.0	4.3
17	BHC, *α*-	0.001	0.003	0.9999	58.0	103.6	7.8	100.2	3.3	100.3	5.1	94.5	18.2	96.3	13.4	97.8	8.1
	BHC, *β*-	0.001	0.003	0.9982	47.6	113.6	2.0	97.6	3.7	98.5	1.8	83.9	14.6	90.3	7.9	102.4	13.8

No.	Analyte	LOD	LOQ	R^2	ME.(%)	Repeatability (*n*=5)						Reproducibility (*n*=11)					
						0.01 mg kg^{-1}		0.02 mg kg^{-1}		0.1 mg kg^{-1}		0.01 mg kg^{-1}		0.02 mg kg^{-1}		0.1 mg kg^{-1}	
						Rec. (%)	CV (%)	Rec. (%)	CV (%)	Rec. (%)	CV (%)	Rec. (%)	CV (%)	Rec. (%)	CV (%)	Rec. (%)	CV (%)
	BHC, δ-	0.001	0.003	0.9993	56.4	83.6	6.8	78.8	7.8	74.5	6.8	77.5	13.3	100.4	17.7	100.6	13.4
18	BHC, γ-	0.001	0.002	0.9973	54.9	109.5	3.6	89.4	1.5	100.6	4.5	95.4	14.0	90.0	20.0	86.9	14.9
19	Bifenox	0.001	0.004	0.9987	338.8	120.9	2.4	102.3	5.1	93.0	2.9	117.0	19.2	111.8	18.7	107.4	17.6
20	Bifenthrin	0.001	0.003	0.9999	210.9	113.5	2.8	99.9	3.4	88.3	2.2	96.8	5.3	95.2	11.7	92.7	5.3
21	Boscalid	0.001	0.003	0.9998	478.4	118.4	3.7	101.2	1.1	85.0	1.9	101.2	6.4	93.7	16.4	94.4	4.7
22	Bromobutide	0.001	0.003	0.9967	71.7	104.3	11.7	99.0	2.4	101.7	5.6	100.4	7.6	90.3	9.7	93.3	6.2
23	Bromophos-methyl	0.001	0.003	0.9988	137.8	110.5	4.5	101.2	1.5	90.6	3.3	96.3	13.2	94.0	14.8	87.0	13.4
24	Bromophos-ethyl	0.001	0.003	0.9993	116.7	109.8	5.8	105.8	4.7	92.8	3.5	92.0	7.0	92.7	15.8	88.0	8.0
25	Bromopropylate	0.001	0.003	0.9998	362.5	119.6	2.7	101.7	2.5	83.3	2.9	98.8	2.7	97.1	8.2	93.1	3.5
26	Bupirimate	0.001	0.002	0.9995	139.9	87.2	4.5	100.6	7.8	91.4	3.5	96.1	6.9	91.8	13.5	90.4	7.2
27	Buprofezin	0.001	0.004	0.9993	93.6	107.5	0.8	99.2	5.1	92.3	2.3	95.9	5.2	95.3	12.3	89.7	6.5
28	Butachlor	0.002	0.006	0.9988	130.0	112.9	3.3	108.8	2.9	97.5	3.5	100.6	8.5	96.6	14.6	92.6	6.9
29	Butafenacil	0.001	0.004	0.9994	>500	117.0	4.1	94.0	1.5	79.4	3.0	101.2	1.7	96.3	10.3	100.3	6.7
30	Butralin	0.001	0.003	0.9959	162.4	112.7	6.2	118.5	1.4	99.5	4.9	101.6	6.6	94.7	12.3	98.9	2.8
31	Butylate	0.001	0.003	0.9974	101.3	110.6	7.9	93.9	6.5	115.8	9.6	93.8	4.1	95.8	3.4	94.7	3.7
32	Cadusafos	0.001	0.003	0.9985	202.0	106.4	6.0	109.7	0.5	109.7	5.1	92.4	5.0	89.3	15.5	94.7	1.7
33	Carbophenothion	0.001	0.003	0.9995	218.1	114.5	4.1	99.1	2.0	76.2	2.9	97.1	10.8	94.4	17.4	91.7	6.8
34	Carboxin	0.001	0.003	0.9998	185.8	119.1	4.2	100.6	6.0	89.3	3.4	97.1	3.4	93.8	14.0	89.3	11.6
35	Carfentrazone-ethyl	0.001	0.004	0.9991	178.4	116.6	4.0	102.0	2.8	79.2	3.2	123.6	33.5	125.6	37.1	114.0	33.9
36	Chinomethionat	0.001	0.003	0.9996	91.7	76.0	7.4	64.8	2.0	70.3	2.7	109.1	13.8	106.7	17.0	102.3	11.7
37	Chlorbenside	0.001	0.002	0.9995	99.4	108.2	2.1	102.4	4.2	92.3	2.3	94.8	4.7	91.5	10.9	86.9	7.6
38	Chlorbufam	0.001	0.004	0.9989	308.8	104.5	1.7	115.2	3.6	106.1	4.1	103.9	15.1	99.8	12.3	94.0	12.2
39	Chlordane-*cis*	0.001	0.002	0.9992	63.1	113.9	4.3	99.0	5.4	93.8	3.0	93.7	8.5	93.7	13.6	89.7	7.4

No.	Analyte	LOD	LOQ	R^2	ME.(%)	Repeatability (*n*=5)						Reproducibility (*n*=11)					
						0.01 mg kg^{-1}		0.02 mg kg^{-1}		0.1 mg kg^{-1}		0.01 mg kg^{-1}		0.02 mg kg^{-1}		0.1 mg kg^{-1}	
						Rec. (%)	CV (%)	Rec. (%)	CV (%)	Rec. (%)	CV (%)	Rec. (%)	CV (%)	Rec. (%)	CV (%)	Rec. (%)	CV (%)
	Chlordane-*trans*	0.001	0.002	0.9991	53.0	111.6	4.2	102.4	2.8	95.4	3.1	96.7	8.8	93.2	13.8	90.2	7.9
40	Chlorethoxyfos	0.002	0.005	0.9987	68.9	106.1	7.4	104.2	4.6	108.0	6.6	88.9	10.6	98.2	23.4	97.3	23.8
41	Chlorfenapyr	0.001	0.002	0.9997	135.8	115.8	0.5	103.5	4.4	91.6	3.3	102.1	13.8	98.4	14.2	93.8	12.7
42	Chlorfenson	0.001	0.002	0.9999	98.5	89.0	6.4	105.1	3.7	108.4	0.5	100.2	16.0	95.1	18.2	89.4	14.1
43	Chlorflurenol-methyl	0.001	0.002	0.9999	268.7	113.3	4.7	103.1	4.9	87.4	4.9	96.6	5.0	93.8	13.3	91.5	8.6
44	Chlornitrofen	0.001	0.003	0.9989	178.0	116.4	2.6	101.6	6.3	81.7	2.7	105.2	8.5	100.9	12.8	98.9	3.1
45	Chlorobenzilate	0.001	0.002	0.9997	148.9	117.5	1.7	108.8	3.4	96.6	3.2	97.5	1.9	94.9	9.6	91.8	4.7
46	Chloropropylate	0.001	0.002	0.9994	145.2	103.4	6.9	84.9	2.9	105.1	6.0	96.8	2.6	94.8	9.7	92.4	4.6
47	Chloroneb	0.001	0.002	0.9986	84.6	117.5	1.7	108.8	3.4	96.6	3.2	91.7	7.1	88.2	14.5	97.0	1.6
48	Chlorothalonil	0.001	0.003	0.9989	89.9	84.8	2.4	N.D.	N.D.	N.D.	N.D.	81.1	25.2	43.7	87.0	80.1	23.8
49	Chlorpropham	0.001	0.003	0.9987	217.6	113.8	1.0	104.3	5.4	107.4	5.0	91.5	16.8	92.2	15.3	92.4	1.9
50	Chlorpyrifos	0.001	0.002	0.9987	104.5	97.3	1.2	107.1	1.2	100.4	2.8	94.9	12.1	89.8	19.6	87.3	12.5
51	Chlorpyrifos-methyl	0.001	0.003	0.9985	114.2	106.4	7.6	98.6	3.5	95.6	4.8	91.0	15.1	93.8	11.8	95.8	6.6
52	Chlorthal-dimethyl	0.001	0.002	0.9992	69.8	115.0	4.5	105.3	2.4	96.8	4.2	93.5	8.6	90.1	16.5	87.4	11.7
53	Chlorthion	0.001	0.004	0.9980	453.7	107.7	8.2	106.7	4.1	77.3	4.6	102.9	15.7	94.8	24.3	92.9	10.3
54	Chlorthiophos	0.001	0.002	0.9996	151.0	114.6	2.0	102.7	3.5	83.2	3.0	93.0	9.7	91.8	16.8	90.2	7.9
55	Chlozolinate	0.001	0.002	0.9992	72.3	110.9	3.1	102.9	3.3	87.8	3.5	92.4	14.6	93.8	12.4	91.4	5.9
56	Cinmethylin	0.002	0.006	0.9985	85.8	97.4	14.6	95.5	5.4	99.6	1.0	89.2	7.9	89.6	15.8	94.3	3.3
57	Clomazone	0.001	0.003	0.9983	107.8	109.7	4.3	101.2	2.9	99.0	3.9	92.3	17.4	94.0	14.5	93.9	8.7
58	Coumaphos	0.001	0.004	0.9993	>500	116.4	4.6	80.1	3.4	70.9	2.7	102.0	9.5	96.5	21.4	97.3	10.6
59	Cyanophos	0.001	0.002	0.9990	156.0	109.2	5.2	94.4	3.6	83.6	6.3	87.9	22.0	88.7	18.8	91.6	5.9
60	Cyflufenamid	0.001	0.002	0.9997	210.0	95.9	3.1	104.8	5.2	86.6	3.6	100.6	6.0	95.8	11.9	94.0	4.5
61	Cyfluthrin	0.002	0.006	0.9907	>500	113.6	3.1	114.8	1.7	101.3	2.3	107.3	11.8	104.7	13.6	108.0	10.8

No.	Analyte	LOD	LOQ	R^2	ME.(%)	Repeatability (*n*=5)						Reproducibility (*n*=11)					
						0.01 mg kg^{-1}		0.02 mg kg^{-1}		0.1 mg kg^{-1}		0.01 mg kg^{-1}		0.02 mg kg^{-1}		0.1 mg kg^{-1}	
						Rec. (%)	CV (%)	Rec. (%)	CV (%)	Rec. (%)	CV (%)	Rec. (%)	CV (%)	Rec. (%)	CV (%)	Rec. (%)	CV (%)
62	Cyhalofop-butyl	0.001	0.002	0.9996	392.0	107.7	2.3	99.1	1.6	82.8	2.4	94.5	7.7	91.7	14.6	89.4	5.7
63	Cyhalothrin, *γ*-	0.002	0.005	0.9998	461.1	107.5	3.3	95.6	1.6	88.5	3.4	102.2	6.7	102.4	9.3	111.5	12.5
	Cyhalothrin, *λ*-	0.001	0.004	0.9996	>500	93.3	4.1	21.4	3.6	72.8	4.9	107.7	13.4	105.6	12.6	111.7	11.2
64	Cypermethrin	0.002	0.007	0.9996	>500	114.4	4.2	103.0	2.9	111.2	2.6	109.8	13.4	107.2	13.5	108.2	10.4
65	Cyprazine	0.001	0.002	0.9991	83.9	111.3	3.9	99.7	2.9	89.1	3.5	85.8	9.2	88.0	11.2	90.7	3.6
66	Cyprodinil	0.001	0.002	0.9996	113.2	110.3	5.6	108.5	2.3	94.4	3.7	92.6	3.6	91.9	10.9	88.1	7.8
67	DDD, *p,p'*-	0.001	0.002	0.9998	81.0	113.1	2.5	104.2	5.5	98.4	2.8	100.2	13.3	96.8	18.7	92.5	11.1
	DDE, *p,p'*-	0.001	0.002	0.9998	66.7	107.4	2.7	116.7	1.8	112.2	2.2	92.4	0.7	91.5	7.0	87.6	6.2
	DDT, *o,p'*-	0.001	0.004	0.9986	10.3	113.3	3.3	95.0	5.0	72.3	4.5	101.4	30.8	101.5	33.4	111.3	29.5
	DDT, *p,p'*-	0.001	0.004	0.9984	18.4	115.5	3.0	76.8	9.6	83.9	2.4	114.8	23.1	109.9	29.6	110.2	21.9
68	Deltamethrin	0.003	0.009	0.9997	>500	101.2	2.5	87.2	2.5	83.9	0.7	97.1	0.8	86.2	12.7	89.3	9.3
69	Desmetryn	0.001	0.003	0.9991	120.2	111.0	3.0	107.6	1.7	97.7	4.5	90.1	13.1	93.6	11.9	97.1	2.5
70	Dialifor	0.002	0.005	0.9991	379.6	112.9	6.6	96.0	4.7	82.3	1.2	95.3	21.0	94.5	21.5	95.2	9.1
71	Di-Allate	0.001	0.003	0.9993	79.7	104.8	6.2	100.1	2.1	102.4	5.1	93.2	4.4	90.6	12.4	86.3	15.0
72	Diazinon	0.001	0.004	0.9986	90.2	106.4	8.9	104.4	3.0	101.0	4.4	94.2	10.3	87.7	16.2	92.8	4.0
73	Dichlobenil	0.002	0.006	0.9993	53.2	105.0	7.2	98.6	2.7	113.7	6.1	93.9	1.7	90.6	12.7	92.8	3.0
74	Dichlofenthion	0.001	0.002	0.9978	92.3	107.8	4.9	105.4	3.1	98.7	4.2	92.6	7.6	89.5	15.8	92.8	1.9
75	Dichlofluanid	0.001	0.003	0.9690	219.5	100.0	11.8	116.0	4.0	70.5	4.4	83.0	28.2	69.0	36.6	81.0	11.1
76	Dichlormid	0.001	0.004	0.9993	99.5	103.0	9.3	103.8	2.8	113.9	7.2	101.4	15.9	101.1	12.4	101.2	9.0
77	Diclobutrazol	0.001	0.002	0.9998	>500	116.1	3.6	109.3	2.1	84.5	4.9	97.5	5.5	96.6	8.1	95.0	4.4
78	Diclofop-methyl	0.001	0.002	0.9998	159.5	117.4	3.7	99.3	2.9	82.8	2.2	93.7	2.6	93.3	11.4	88.2	6.4
79	Dicloran	0.001	0.004	0.9991	176.6	108.1	6.2	112.6	4.9	103.3	5.0	98.5	13.1	94.0	22.4	95.3	10.4
80	Dicofol	0.001	0.002	0.9999	54.4	109.7	6.6	105.9	1.2	96.2	2.6	92.7	5.9	85.2	9.5	90.2	10.0

No.	Analyte	LOD	LOQ	R^2	ME.(%)	Repeatability (*n*=5)						Reproducibility (*n*=11)					
						0.01 mg kg^{-1}		0.02 mg kg^{-1}		0.1 mg kg^{-1}		0.01 mg kg^{-1}		0.02 mg kg^{-1}		0.1 mg kg^{-1}	
						Rec. (%)	CV (%)	Rec. (%)	CV (%)	Rec. (%)	CV (%)	Rec. (%)	CV (%)	Rec. (%)	CV (%)	Rec. (%)	CV (%)
81	Dicrotophos	0.002	0.005	0.9997	>500	108.6	2.6	86.4	5.6	81.7	2.8	89.2	28.8	87.6	27.3	84.1	24.2
82	Diethatyl-ethyl	0.002	0.005	0.9998	84.3	114.6	2.0	111.8	2.0	99.8	2.9	101.4	11.3	99.4	16.6	97.1	9.3
83	Diethofencarb	0.001	0.002	0.9988	371.8	112.0	5.1	117.3	3.0	94.5	6.3	99.2	8.2	94.6	15.3	90.9	10.7
84	Difenoconazole	0.001	0.004	0.9976	>500	101.7	4.7	92.0	3.9	73.7	5.6	100.3	6.9	97.7	8.5	97.7	2.0
85	Diflufenican	0.001	0.002	0.9997	301.8	117.8	3.4	101.2	2.2	80.8	3.0	98.6	4.7	95.5	2.9	92.4	2.5
86	Dimepiperate	0.001	0.003	0.9993	122.8	113.8	5.0	106.9	2.3	93.5	2.8	93.7	7.3	90.1	15.3	87.1	10.5
87	Dimethachlor	0.001	0.002	0.9988	85.1	118.4	1.5	114.7	2.3	112.5	5.1	98.2	13.9	96.7	12.6	87.5	19.1
88	Dimethametryn	0.001	0.002	0.9994	123.1	112.6	5.3	108.7	1.5	93.0	3.4	96.4	2.9	94.1	9.6	91.0	6.1
89	Dimethenamid	0.001	0.003	0.9987	80.2	111.5	3.7	109.8	2.2	104.7	4.1	92.8	7.9	89.9	17.1	87.2	15.1
90	Dimethipin	0.002	0.005	1.0000	92.5	79.9	0.7	72.3	2.2	75.1	0.7	118.0	31.6	105.1	34.7	106.4	31.5
91	Dimethomorph (*E*)	0.001	0.004	0.9995	>500	117.1	3.9	104.3	1.0	74.8	3.5	103.4	6.3	99.3	8.4	101.8	8.3
	Dimethomorph (*Z*)	0.001	0.004	0.9999	>500	117.1	4.2	91.5	0.8	77.4	2.8	102.4	5.4	98.8	7.9	102.6	7.1
92	Dimethylvinphos (*E*)	0.001	0.004	0.9988	331.6	107.9	7.3	117.0	5.7	110.7	5.0	90.5	10.7	88.1	11.4	83.1	10.1
	Dimethylvinphos (*Z*)	0.001	0.004	0.9990	416.1	111.1	8.2	101.7	5.0	85.6	3.2	91.7	10.2	86.9	10.3	83.5	10.3
93	Diniconazole	0.001	0.003	0.9996	>500	116.8	2.2	105.6	1.5	75.8	5.0	102.9	2.2	98.4	6.1	96.9	6.0
94	Dinitramine	0.002	0.005	0.9977	165.8	107.7	2.5	116.4	1.1	102.3	3.4	100.3	9.7	92.5	15.4	90.7	9.8
95	Dioxathion	0.001	0.002	0.9991	59.7	116.2	2.0	91.0	7.4	83.5	2.8	85.8	23.1	93.1	10.2	91.2	9.1
96	Diphenamid	0.001	0.003	0.9994	79.4	115.5	2.8	114.5	2.0	98.0	3.9	95.3	3.6	92.7	11.4	90.1	7.6
97	Diphenylamine	0.001	0.003	0.9994	153.3	107.3	5.2	103.1	3.5	106.8	4.6	89.7	11.4	88.8	15.7	89.8	7.3
98	Dithiopyr	0.001	0.002	0.9983	71.6	112.3	4.7	108.4	2.9	99.2	3.3	96.4	2.3	92.6	7.1	88.4	6.0
99	Edifenphos	0.002	0.006	0.9997	>500	108.0	5.0	86.0	3.2	71.8	2.8	96.7	3.5	93.5	7.2	90.9	14.2
100	Endosulfan, *α*-	0.001	0.002	0.9993	30.4	113.4	0.4	101.1	3.2	95.2	3.2	89.7	8.3	90.2	14.4	88.5	9.8
	Endosulfan, *β*-	0.001	0.003	0.9996	44.4	109.1	1.7	102.1	5.8	93.7	1.2	91.7	19.0	94.8	16.2	94.4	6.8

No.	Analyte	LOD	LOQ	R^2	ME.(%)	Repeatability (*n*=5)						Reproducibility (*n*=11)					
						0.01 mg kg^{-1}		0.02 mg kg^{-1}		0.1 mg kg^{-1}		0.01 mg kg^{-1}		0.02 mg kg^{-1}		0.1 mg kg^{-1}	
						Rec. (%)	CV (%)	Rec. (%)	CV (%)	Rec. (%)	CV (%)	Rec. (%)	CV (%)	Rec. (%)	CV (%)	Rec. (%)	CV (%)
	Endosulfan, sulfate-	0.001	0.002	0.9996	92.9	114.3	2.8	107.7	4.4	91.4	2.8	96.2	4.1	90.4	14.2	98.2	12.9
101	Endrin	0.001	0.002	0.9997	74.9	114.2	2.2	105.7	4.3	95.0	2.5	96.5	15.2	93.3	19.0	90.1	12.1
	δ-keto-Endrin	0.001	0.003	0.9966	52.1	111.7	2.5	101.5	5.3	89.7	1.4	100.1	3.0	93.9	9.6	95.2	9.1
102	EPN	0.002	0.005	0.9989	311.8	113.5	3.8	99.4	3.1	72.3	3.5	93.7	12.5	102.0	17.1	105.8	6.4
103	Epoxiconazole	0.001	0.003	0.9996	185.5	113.1	1.6	104.0	2.3	84.6	2.4	101.4	12.3	99.0	15.9	96.7	10.6
104	EPTC	0.001	0.003	0.9966	50.6	102.6	14.4	91.3	9.8	113.8	8.3	100.0	4.5	93.8	11.8	88.5	14.7
105	Etaconazole-1	0.001	0.003	0.9997	136.2	115.9	2.8	110.7	2.2	90.6	3.6	103.5	7.4	100.3	15.2	96.8	7.4
	Etaconazole-2	0.001	0.002	0.9999	100.4	114.1	2.7	114.7	1.0	95.1	3.9	99.4	1.6	93.6	15.0	93.5	6.4
106	Ethalfluralin	0.001	0.004	0.9971	146.6	106.8	6.4	111.9	1.9	113.0	5.5	100.7	2.3	94.4	11.4	91.2	9.4
107	Ethion	0.001	0.003	0.9994	198.5	114.4	3.8	99.4	3.1	76.5	4.0	98.9	10.0	94.6	18.0	93.3	4.7
108	Ethofumesate	0.002	0.006	0.9992	95.3	114.1	2.0	113.1	2.6	98.5	3.3	93.1	9.7	88.9	14.2	86.7	12.6
109	Ethoprophos	0.001	0.002	0.9985	227.7	106.0	5.3	112.1	2.0	112.4	8.5	88.1	11.0	89.1	11.5	91.1	7.0
110	Ethychlozate	0.001	0.002	0.9998	406.2	110.4	5.9	112.0	2.4	84.7	6.1	93.3	11.5	97.9	12.8	88.0	6.7
111	Etoxazole	0.001	0.002	0.9996	218.4	104.8	3.4	98.3	2.3	82.4	2.4	96.2	6.5	91.9	11.5	93.1	3.5
112	Etridiazole	0.002	0.007	0.9960	85.7	100.8	5.6	90.5	4.0	91.3	8.0	97.2	6.6	97.4	16.2	94.8	21.1
113	Fenamidone	0.001	0.002	0.9996	159.3	106.3	1.0	108.6	1.5	88.3	2.3	99.7	3.3	95.2	8.7	95.6	4.5
114	Fenarimol	0.001	0.003	0.9996	297.8	112.1	2.4	103.7	1.7	86.9	2.7	98.5	4.9	97.9	4.2	96.9	4.3
115	Fenbuconazole	0.001	0.003	0.9997	451.8	109.1	1.7	98.4	1.5	83.8	2.1	96.0	6.4	90.8	15.7	91.5	5.0
116	Fenchlorphos	0.001	0.002	0.9977	125.3	103.6	6.7	101.3	3.5	94.4	4.0	88.4	12.6	92.6	10.1	93.0	2.8
117	Fenclorim	0.001	0.002	0.9958	108.8	97.4	9.4	95.3	5.3	93.0	5.4	94.5	9.6	94.7	7.3	91.4	2.1
118	Fenfuram	0.001	0.002	0.9993	151.5	113.3	3.5	116.7	2.1	103.5	4.1	92.0	3.1	89.1	11.0	88.0	8.5
119	Fenitrothion	0.002	0.005	0.9985	345.4	109.8	5.0	106.5	2.9	86.0	4.6	94.9	14.4	91.0	20.3	88.7	14.5
120	Fenobucarb	0.002	0.006	0.9987	236.5	108.7	6.3	117.5	5.2	104.5	3.6	95.7	22.4	97.6	11.4	101.7	13.3

No.	Analyte	LOD	LOQ	R^2	ME.(%)	Repeatability (*n*=5)						Reproducibility (*n*=11)					
						0.01 mg kg^{-1}		0.02 mg kg^{-1}		0.1 mg kg^{-1}		0.01 mg kg^{-1}		0.02 mg kg^{-1}		0.1 mg kg^{-1}	
						Rec. (%)	CV (%)	Rec. (%)	CV (%)	Rec. (%)	CV (%)	Rec. (%)	CV (%)	Rec. (%)	CV (%)	Rec. (%)	CV (%)
121	Fenothiocarb	0.001	0.002	0.9998	216.7	116.3	3.9	97.6	2.9	82.6	2.3	93.6	4.5	93.0	11.4	89.2	9.1
122	Fenoxanil	0.001	0.003	0.9998	>500	62.6	7.6	109.6	2.6	118.2	3.7	101.0	7.4	99.9	13.7	87.0	6.2
123	Fenpropathrin	0.001	0.003	0.9992	175.3	116.9	2.5	101.3	2.6	88.1	2.1	99.7	12.0	95.8	14.4	94.7	4.3
124	Fenpropimorph	0.001	0.002	0.9984	125.4	112.8	4.5	113.0	1.4	101.6	2.1	94.0	6.2	91.0	12.8	92.0	10.0
125	Fenpyrazamine	0.002	0.006	0.9984	>500	92.6	4.3	74.9	2.4	100.1	4.4	92.1	6.3	88.1	18.0	98.7	16.6
126	Fenson	0.001	0.002	0.9996	60.2	113.4	3.1	112.4	1.9	98.5	2.8	96.2	9.6	92.6	17.0	89.1	13.1
127	Fenthion	0.001	0.003	0.9989	133.0	113.6	5.4	106.4	2.6	91.1	4.2	90.8	14.5	94.6	13.7	94.4	3.2
128	Fenvalerate-1	0.002	0.006	0.9996	>500	109.8	3.6	118.5	2.4	101.9	3.5	107.1	14.1	102.5	17.9	106.0	6.8
	Fenvalerate-2	0.002	0.006	0.9995	>500	120.9	3.5	109.3	3.5	96.7	4.9	107.6	11.6	99.0	14.0	105.6	4.9
129	Fipronil	0.001	0.003	0.9996	356.9	117.4	7.0	117.8	2.8	90.6	6.5	102.5	4.0	98.0	8.5	97.7	5.2
130	Flamprop-isopropyl	0.001	0.003	0.9998	99.9	118.0	3.8	115.6	3.1	92.8	2.9	100.0	0.2	99.3	8.1	96.7	4.0
131	Fluacrypyrim	0.001	0.003	0.9996	200.4	111.0	1.4	104.1	2.5	82.3	3.1	99.3	4.2	98.2	9.3	93.9	4.0
132	Fluazifop-butyl	0.001	0.002	0.9995	201.2	115.6	1.8	105.2	2.7	80.9	4.0	97.3	6.0	95.3	13.5	90.1	7.6
133	Fluchloralin	0.002	0.005	0.9963	137.1	108.8	7.9	119.3	0.6	111.9	5.2	108.7	6.4	99.2	12.0	93.9	9.6
134	Flucythrinate-1	0.002	0.005	0.9995	>500	116.6	2.0	110.2	2.0	92.1	3.4	99.1	18.5	99.6	16.9	107.5	11.3
	Flucythrinate-2	0.002	0.006	0.9993	>500	116.9	1.0	105.2	3.9	117.4	3.0	100.3	17.0	104.9	15.7	107.7	9.7
135	Fluensulfone	0.001	0.003	0.9985	182.4	97.0	5.6	99.1	2.0	103.5	5.4	90.7	16.4	90.4	15.4	93.7	5.3
136	Flufenpyr-ethyl	0.001	0.002	0.9993	254.4	113.0	4.5	110.7	2.3	82.7	4.7	108.9	5.9	104.3	11.0	103.2	3.3
137	Flumetralin	0.001	0.004	0.9984	157.7	114.1	6.9	113.5	1.8	91.9	4.2	106.0	8.0	102.7	11.4	103.0	3.9
138	Flumioxazin	0.001	0.004	0.9991	>500	111.7	2.4	73.6	1.0	85.4	5.2	107.0	11.8	99.8	20.9	104.7	1.4
139	Fluopyram	0.001	0.002	0.9998	227.0	117.3	5.7	114.4	1.6	92.4	3.9	98.8	2.0	96.6	7.3	92.5	5.8
140	Fluorochloridone	0.001	0.002	0.9999	129.7	109.8	3.1	90.4	3.9	80.6	0.8	97.5	5.4	91.2	12.6	87.1	2.7
141	Fluquinconazole	0.001	0.003	0.9976	232.1	108.9	4.8	118.4	1.7	100.3	3.7	99.5	3.6	99.0	9.4	97.7	6.5

No.	Analyte	LOD	LOQ	R^2	ME.(%)	Repeatability (*n*=5)						Reproducibility (*n*=11)					
						0.01 mg kg^{-1}		0.02 mg kg^{-1}		0.1 mg kg^{-1}		0.01 mg kg^{-1}		0.02 mg kg^{-1}		0.1 mg kg^{-1}	
						Rec. (%)	CV (%)	Rec. (%)	CV (%)	Rec. (%)	CV (%)	Rec. (%)	CV (%)	Rec. (%)	CV (%)	Rec. (%)	CV (%)
142	Flusilazole	0.001	0.002	0.9995	137.5	117.9	2.7	117.2	2.4	94.4	3.9	98.5	2.9	94.2	10.2	92.3	5.7
143	Flutianil	0.001	0.003	0.9996	365.1	116.1	4.7	97.1	0.9	81.6	2.5	102.3	4.7	97.1	10.6	97.9	6.5
144	Fluvalinate-1	0.003	0.008	0.9994	>500	105.0	8.2	94.6	1.6	81.7	3.3	116.4	10.7	109.9	8.4	122.5	21.4
	Fluvalinate-2	0.003	0.008	0.9992	>500	108.3	7.7	94.6	3.1	78.8	7.0	117.1	10.4	108.1	6.5	121.3	22.4
145	Fluxapyroxad	0.001	0.003	0.9997	496.5	110.0	2.3	100.1	1.2	80.1	3.5	100.0	2.6	95.5	8.3	94.1	3.6
146	Fonofos	0.001	0.002	0.9977	92.1	109.2	5.4	105.4	2.2	101.1	4.3	92.9	7.1	88.0	16.8	92.0	4.2
147	Formothion	0.002	0.005	1.0000	263.4	82.3	9.4	78.4	5.4	76.7	1.9	80.9	9.6	92.7	25.6	91.9	17.0
148	Fthalide	0.001	0.002	0.9994	78.7	105.2	14.1	97.9	2.1	92.3	3.7	101.2	12.9	94.8	21.8	90.6	16.5
149	Halfenprox	0.001	0.004	0.9991	303.5	116.2	3.3	92.2	2.4	79.3	1.9	102.9	11.8	97.6	17.1	100.8	3.6
150	Heptachlor	0.002	0.005	0.9961	73.0	109.2	5.8	97.0	3.2	92.2	5.3	95.8	11.1	91.9	10.2	86.7	9.8
	Heptachlor epoxide	0.001	0.003	0.9996	40.9	110.1	6.4	102.8	4.9	98.8	2.5	96.0	9.3	95.7	12.9	91.0	7.8
151	Heptenophos	0.001	0.003	0.9991	249.7	100.9	6.3	105.7	3.3	115.8	7.9	94.1	23.2	94.4	17.1	99.0	7.8
152	Hexythiazox	0.001	0.002	0.9998	269.5	115.6	5.5	100.1	4.2	79.2	3.9	98.0	5.7	96.1	8.3	91.1	7.8
153	Indanofan	0.001	0.004	0.9994	290.4	105.5	2.8	97.9	2.5	81.8	2.1	102.3	6.1	96.1	11.6	95.2	5.4
154	Indoxacarb	0.001	0.002	0.9998	357.5	115.8	6.1	106.1	3.5	104.4	2.1	94.5	19.2	92.8	15.7	101.2	10.8
155	Ipconazole	0.001	0.003	0.9995	473.0	111.3	1.6	102.3	1.9	85.6	2.5	98.2	2.3	97.1	5.9	96.4	3.8
156	Iprobenfos	0.001	0.004	0.9988	388.1	99.2	3.0	108.9	2.2	96.5	5.4	95.9	8.8	90.1	17.2	86.6	15.3
157	Iprodione	0.002	0.007	0.9998	>500	92.6	5.5	71.3	3.5	63.6	1.8	103.7	17.3	96.9	21.8	105.8	17.0
158	Isazofos	0.001	0.002	0.9978	91.8	116.2	1.0	109.1	1.2	103.7	3.9	88.0	11.1	91.4	11.1	93.5	3.5
159	Isofenphos	0.001	0.003	0.9992	109.3	114.2	4.9	109.4	2.2	94.5	3.5	97.3	4.6	94.4	10.2	91.7	5.2
160	Isofenphos-methyl	0.001	0.002	0.9992	114.6	111.1	3.9	112.6	2.8	94.6	3.8	97.6	6.9	93.6	13.6	91.4	6.0
161	Isoprocarb	0.002	0.005	0.9985	250.0	101.0	8.0	117.8	8.2	102.3	2.7	86.2	18.2	99.4	17.5	99.2	15.2
162	Isopropalin	0.001	0.004	0.9974	136.4	110.9	3.8	116.8	3.3	97.1	3.8	101.8	4.8	96.3	10.3	97.8	3.5

No.	Analyte	LOD	LOQ	R^2	ME.(%)	Repeatability (*n*=5)						Reproducibility (*n*=11)					
						0.01 mg kg^{-1}		0.02 mg kg^{-1}		0.1 mg kg^{-1}		0.01 mg kg^{-1}		0.02 mg kg^{-1}		0.1 mg kg^{-1}	
						Rec. (%)	CV (%)	Rec. (%)	CV (%)	Rec. (%)	CV (%)	Rec. (%)	CV (%)	Rec. (%)	CV (%)	Rec. (%)	CV (%)
163	Isoprothiolane	0.003	0.008	0.9998	130.3	115.1	1.6	112.3	3.0	111.9	2.7	96.9	6.7	92.4	12.5	90.3	8.8
164	Isopyrazam	0.001	0.003	0.9996	>500	118.1	1.0	106.9	2.7	91.3	2.1	100.3	5.1	98.9	6.3	99.8	7.5
165	Isotianil	0.001	0.004	0.9994	>500	114.6	4.9	99.5	1.9	72.9	3.6	106.2	10.5	100.6	12.5	96.0	7.2
166	Isoxadifen-ethyl	0.001	0.002	0.9994	145.2	117.5	4.2	102.9	3.1	81.4	3.0	95.5	10.7	92.0	16.9	93.7	5.4
167	Kresoxim-methyl	0.001	0.002	0.9997	89.2	116.1	3.6	117.4	3.1	102.3	2.0	94.5	6.0	91.8	13.7	91.0	6.7
168	Leptophos	0.001	0.003	0.9995	233.4	110.8	4.6	94.7	4.4	84.0	1.4	98.6	15.2	98.0	16.8	96.1	5.5
169	Mefenpyr-diethyl	0.001	0.003	0.9995	197.8	112.2	1.5	101.3	2.0	81.0	2.7	101.3	1.9	96.9	6.6	93.6	4.2
170	Mepanipyrim	0.001	0.004	1.0000	202.1	98.6	3.5	95.5	2.0	81.8	3.4	98.4	6.1	95.6	13.8	90.5	10.3
171	Mepronil	0.001	0.002	0.9998	454.7	117.4	2.7	104.4	2.3	82.7	3.7	98.3	2.7	95.6	10.2	92.3	6.3
172	Metalaxyl	0.001	0.002	0.9992	90.0	114.3	4.1	110.6	2.9	97.3	4.1	90.6	1.3	89.0	10.4	85.7	10.5
173	Methidathion	0.001	0.003	0.9993	307.0	86.5	6.7	82.6	7.8	87.4	2.1	98.4	19.1	91.8	26.5	88.7	19.1
174	Methoprotryn	0.001	0.002	0.9998	190.1	115.6	3.5	110.3	2.8	82.1	4.4	99.5	5.9	95.7	12.9	93.4	8.0
175	Methoxychlor	0.002	0.005	0.9961	108.2	117.4	3.7	78.4	6.3	85.2	1.1	93.4	12.9	92.2	15.8	90.5	14.7
176	Methyl trithion	0.001	0.003	0.9994	268.3	113.8	5.3	94.6	5.0	73.5	3.8	95.4	21.3	101.9	26.2	95.2	16.6
177	Metolachlor	0.001	0.003	0.9997	109.7	97.4	2.0	115.8	2.7	103.2	3.6	95.3	7.9	91.7	16.2	90.0	10.9
178	Metribuzin	0.001	0.002	0.9992	117.2	116.6	4.4	106.7	2.7	94.9	4.7	91.9	3.7	90.5	12.0	87.3	13.1
179	MGK-264	0.001	0.002	0.9994	92.6	112.3	3.8	107.0	1.7	95.1	3.4	98.7	5.1	94.8	10.9	89.4	8.8
180	Molinate	0.001	0.003	0.9979	78.2	107.2	3.8	99.8	3.6	105.9	7.0	96.3	7.6	93.6	11.7	85.5	19.0
181	Monolinuron	0.001	0.004	0.9989	214.3	111.3	5.5	111.1	6.8	94.8	7.7	82.4	16.2	77.5	25.7	82.7	14.0
182	Myclobutanil	0.001	0.002	0.9998	135.2	116.1	3.5	116.6	2.8	93.8	3.1	98.2	2.1	96.2	8.5	93.5	4.9
183	Nitrothal-isopropyl	0.001	0.004	0.9971	208.3	113.5	6.0	107.0	2.5	101.7	4.7	108.8	9.7	100.3	11.5	103.0	2.1
184	Nonachlor-*cis*	0.001	0.002	0.9997	51.9	115.6	3.3	104.9	3.8	96.6	1.9	87.3	27.0	92.8	14.3	90.6	7.9
	Nonachlor-*trans*	0.001	0.002	0.9998	43.7	111.6	3.9	98.8	0.6	94.3	2.9	88.2	25.0	93.9	15.6	92.5	7.8

No.	Analyte	LOD	LOQ	R^2	ME.(%)	Repeatability (*n*=5)						Reproducibility (*n*=11)					
						0.01 mg kg^{-1}		0.02 mg kg^{-1}		0.1 mg kg^{-1}		0.01 mg kg^{-1}		0.02 mg kg^{-1}		0.1 mg kg^{-1}	
						Rec. (%)	CV (%)	Rec. (%)	CV (%)	Rec. (%)	CV (%)	Rec. (%)	CV (%)	Rec. (%)	CV (%)	Rec. (%)	CV (%)
185	Nuarimol	0.001	0.002	0.9999	242.0	113.5	1.4	104.5	2.0	87.1	3.2	96.1	5.1	95.2	2.8	92.1	2.9
186	Ortho-phenyl phenol	0.001	0.003	0.9987	358.6	98.7	8.0	97.5	1.9	100.8	6.6	91.1	11.1	82.1	24.6	76.1	32.7
187	Oxadiazon	0.001	0.002	0.9996	105.5	117.0	2.4	117.0	2.9	105.8	2.9	97.5	5.7	96.4	11.5	91.4	6.6
188	Oxadixyl	0.001	0.002	0.9998	112.0	119.4	3.8	110.5	3.1	114.8	2.7	102.3	13.7	99.3	18.3	97.7	12.7
189	Oxyfluorfen	0.001	0.004	0.9989	272.7	114.8	5.1	110.5	2.2	83.3	3.9	103.0	11.5	96.8	15.7	99.6	1.2
190	Paclobutrazol	0.001	0.002	0.9994	>500	111.6	5.0	105.2	2.0	80.8	5.9	98.4	3.1	96.8	6.7	93.7	3.8
191	Parathion	0.001	0.004	0.9983	249.0	114.1	4.4	109.3	3.5	103.0	4.7	104.0	9.3	93.2	17.8	94.8	7.6
192	Parathion-methyl	0.002	0.005	0.9984	246.8	107.6	7.1	105.9	3.7	84.9	5.3	98.0	10.1	91.4	20.2	94.3	5.5
193	Penconazole	0.001	0.002	0.9996	104.2	112.7	5.4	110.9	2.4	96.0	3.8	97.5	2.0	94.4	9.2	90.9	6.7
194	Pendimethalin	0.001	0.003	0.9978	201.0	110.8	6.8	119.7	2.9	95.8	4.3	104.3	7.5	93.5	13.0	96.9	0.6
195	Penflufen	0.001	0.002	0.9997	249.4	116.8	2.9	101.8	2.8	80.8	3.6	97.9	3.3	95.6	9.9	93.0	4.4
196	Pentachlorobenzonitrile	0.001	0.003	0.9989	81.3	102.9	8.3	95.4	2.6	97.7	3.0	90.9	13.6	93.3	10.9	93.7	3.7
197	Penthiopyrad	0.001	0.002	0.9997	405.5	110.9	2.8	103.1	2.2	83.4	3.7	102.7	2.2	99.1	6.5	96.8	3.0
198	Pentoxazone	0.001	0.002	0.9997	192.8	116.6	3.3	102.5	2.6	88.8	1.8	100.1	2.1	97.3	7.2	92.7	3.7
199	Permethrin-cis	0.001	0.004	0.9998	417.8	113.8	4.3	113.7	5.4	105.5	2.6	106.9	11.7	96.1	9.7	91.4	3.2
	Permethrin-trans	0.001	0.002	0.9995	369.7	97.0	5.1	93.3	9.3	85.3	1.5	108.9	5.8	97.8	7.6	94.7	3.3
200	Perthane	0.001	0.002	0.9995	93.8	115.7	3.5	103.9	4.2	86.9	2.7	96.9	8.5	94.4	15.5	91.1	8.8
201	Phenthoate	0.002	0.005	0.9991	144.7	111.4	7.4	106.6	2.8	86.4	4.6	99.4	11.7	92.9	20.8	89.7	12.6
202	Phosphamidon (*E*)	0.001	0.004	0.9987	>500	106.7	10.3	103.2	15.2	82.4	5.8	85.8	10.9	79.7	12.3	88.0	13.2
	Phosphamidon (*Z*)	0.002	0.007	0.9990	416.5	110.6	10.3	89.5	9.1	85.0	7.6	90.8	13.7	81.8	13.4	85.7	10.2
203	Phosalone	0.001	0.004	0.9991	>500	113.5	3.8	95.8	5.0	79.0	2.5	89.6	11.1	98.8	21.1	98.4	10.0
204	Phosmet	0.001	0.004	0.9992	236.8	106.7	9.9	87.3	3.8	74.4	2.6	98.7	19.8	79.4	11.3	100.8	16.7
205	Picoxystrobin	0.001	0.003	0.9999	148.3	114.3	3.9	113.7	2.2	86.2	2.4	98.0	4.1	95.8	11.3	91.6	7.1

No.	Analyte	LOD	LOQ	R^2	ME.(%)	Repeatability (*n*=5)						Reproducibility (*n*=11)					
						0.01 mg kg^{-1}		0.02 mg kg^{-1}		0.1 mg kg^{-1}		0.01 mg kg^{-1}		0.02 mg kg^{-1}		0.1 mg kg^{-1}	
						Rec. (%)	CV (%)	Rec. (%)	CV (%)	Rec. (%)	CV (%)	Rec. (%)	CV (%)	Rec. (%)	CV (%)	Rec. (%)	CV (%)
206	Piperonyl butoxide	0.001	0.002	0.9996	364.4	108.3	1.2	102.1	2.5	80.7	3.3	98.8	9.2	94.1	13.4	91.9	6.6
207	Pirimicarb	0.001	0.002	0.9989	132.2	110.2	5.1	104.8	2.8	98.7	5.3	90.6	10.5	89.3	9.9	92.3	1.0
208	Pirimiphos-ethyl	0.001	0.002	0.9982	115.8	111.1	4.6	112.4	1.8	95.0	4.1	94.5	8.2	93.5	14.7	91.0	7.0
209	Pirimiphos-methyl	0.001	0.002	0.9990	154.3	108.2	3.6	106.3	2.2	97.8	3.9	90.4	11.7	89.6	11.1	86.2	13.2
210	Pretilachlor	0.001	0.004	0.9998	162.7	97.2	1.5	108.6	4.8	100.9	3.2	98.1	9.7	98.3	16.0	98.1	11.2
211	Prochloraz	0.001	0.003	0.9982	>500	97.1	5.6	82.6	1.2	97.7	4.2	93.1	26.6	88.0	39.5	92.9	18.7
	2,4,6-trichlorophenol	0.001	0.002	0.9985	233.7	80.7	12.8	95.7	5.3	94.0	5.1	86.9	10.0	83.5	23.0	78.1	25.5
212	Procymidone	0.001	0.002	0.9995	72.4	115.8	4.1	104.1	3.1	92.3	3.7	94.2	8.3	94.5	12.1	89.1	8.7
213	Prodiamine	0.001	0.003	0.9981	201.0	113.5	4.4	119.0	2.5	103.1	4.7	98.8	2.4	95.8	11.8	95.3	1.6
214	Profenofos	0.001	0.003	0.9995	>500	116.1	3.5	105.1	3.0	90.0	5.9	91.8	7.4	88.9	13.8	86.0	7.6
215	Profluralin	0.001	0.004	0.9971	118.3	108.9	2.5	115.3	4.2	105.5	4.8	101.7	2.7	97.7	9.9	95.5	6.4
216	Prohydrojasmon	0.001	0.004	0.9984	144.8	107.4	5.4	113.7	3.0	101.6	3.4	95.1	10.3	90.2	16.7	92.0	3.4
217	Prometon	0.001	0.002	0.9990	168.0	112.3	4.1	108.7	2.3	101.1	4.8	92.4	8.9	89.4	18.4	92.7	3.9
218	Prometryn	0.001	0.002	0.9992	106.9	106.9	5.9	104.7	2.4	95.8	3.7	91.2	3.3	91.3	10.0	86.8	10.6
219	Propachlor	0.001	0.003	0.9989	131.6	102.3	4.0	110.6	2.5	115.0	2.5	88.1	20.9	95.1	13.4	97.2	7.3
220	Propanil	0.001	0.003	0.9995	181.1	113.0	5.2	112.2	4.8	97.3	4.4	94.6	7.0	90.7	10.2	89.8	4.3
221	Propazine	0.001	0.002	0.9987	104.7	110.5	3.8	102.0	1.9	96.6	4.5	91.1	13.0	90.1	11.3	90.3	3.3
222	Propetamphos	0.001	0.003	0.9979	154.9	108.6	6.0	107.9	2.6	98.9	5.9	90.8	11.0	97.5	0.7	91.9	2.2
223	Propham	0.001	0.003	0.9994	187.5	94.6	8.0	103.2	10.7	108.8	7.3	83.9	12.3	87.2	11.7	90.6	5.3
224	Propiconazole-1	0.001	0.002	0.9996	168.8	111.6	2.4	104.5	3.2	86.0	3.1	93.9	4.2	94.8	5.9	93.4	4.0
	Propiconazole-2	0.001	0.002	0.9999	176.9	116.7	5.3	105.5	2.4	88.0	2.4	97.6	2.1	97.1	7.2	92.9	4.8
225	Propisochlor	0.001	0.002	0.9983	89.9	112.5	1.7	117.3	3.5	108.0	3.3	103.4	8.9	95.7	11.0	88.5	11.2
226	Propyzamide	0.001	0.002	0.9990	137.1	96.9	2.2	111.1	1.4	99.8	4.1	90.4	8.7	91.6	14.3	93.1	3.2

No.	Analyte	LOD	LOQ	R^2	ME.(%)	Repeatability (*n*=5)						Reproducibility (*n*=11)					
						0.01 mg kg^{-1}		0.02 mg kg^{-1}		0.1 mg kg^{-1}		0.01 mg kg^{-1}		0.02 mg kg^{-1}		0.1 mg kg^{-1}	
						Rec. (%)	CV (%)	Rec. (%)	CV (%)	Rec. (%)	CV (%)	Rec. (%)	CV (%)	Rec. (%)	CV (%)	Rec. (%)	CV (%)
227	Prothiofos	0.001	0.002	0.9996	167.3	110.5	7.8	110.8	3.7	110.0	2.3	92.7	7.7	88.0	13.6	85.5	8.7
228	Pyracarbolid	0.002	0.007	0.9999	222.9	114.7	2.1	111.4	4.8	100.2	5.1	87.9	17.2	87.8	13.9	88.4	8.6
229	Pyraclofos	0.002	0.005	0.9995	>500	110.9	4.5	103.9	3.7	94.5	3.4	82.3	6.6	78.0	9.3	94.5	24.0
230	Pyraflufen-ethyl	0.001	0.002	0.9998	216.9	110.9	1.0	100.6	2.5	77.4	3.6	98.0	4.3	94.6	9.3	90.6	4.3
231	Pyrazophos	0.001	0.003	0.9986	416.8	114.0	4.3	90.4	1.1	71.6	3.4	101.0	18.2	95.0	23.7	96.0	7.9
232	Pyridalyl	0.001	0.004	0.9995	>500	109.9	3.3	94.1	0.6	77.2	2.3	102.3	3.8	96.9	9.4	96.8	4.6
233	Pyrifenox	0.001	0.002	0.9995	127.0	109.4	4.3	101.6	1.5	84.9	4.4	100.5	2.3	93.0	11.0	91.1	8.6
234	Pyriftalid	0.001	0.002	0.9994	380.1	110.0	2.4	98.1	2.5	79.4	3.0	96.3	13.3	91.2	18.9	90.7	9.3
235	Pyrimethanil	0.001	0.002	0.9990	125.4	108.5	3.3	105.6	2.0	99.9	4.7	91.5	10.2	88.0	15.7	91.6	3.7
236	Pyriminobac-methyl (*E*)	0.001	0.003	0.9997	195.8	109.2	2.5	101.0	2.2	79.0	3.8	97.1	8.0	94.6	13.4	93.6	5.4
	Pyriminobac-methyl (*Z*)	0.001	0.002	0.9994	121.6	111.7	2.1	107.0	2.6	88.0	3.2	97.1	6.0	94.9	13.6	93.3	6.0
237	Quinalphos	0.003	0.009	0.9996	137.0	96.5	4.5	109.7	3.4	90.7	4.4	96.8	8.7	90.5	17.1	88.1	12.0
238	Quinoxyfen	0.001	0.002	0.9998	85.9	115.0	3.2	101.6	2.8	84.7	2.3	94.7	3.6	93.0	9.1	88.6	6.0
239	Quintozene	0.002	0.005	0.9988	104.2	100.8	7.6	119.4	7.7	116.5	5.6	99.9	16.7	94.8	17.1	93.1	9.4
240	Quizalofop-ethyl	0.001	0.003	0.9994	461.7	106.9	2.5	94.6	0.8	78.5	2.2	98.7	1.4	99.5	0.4	92.3	0.5
241	Silafluofen	0.001	0.002	0.9993	282.6	116.8	2.2	92.3	1.9	80.8	1.9	97.9	6.6	94.2	10.4	95.5	3.1
242	Simeconazole	0.001	0.003	0.9994	198.4	114.6	3.4	119.9	1.9	105.4	4.0	94.8	1.6	93.2	10.1	90.0	9.2
243	Simetryn	0.001	0.002	0.9993	129.0	110.1	1.5	111.5	2.1	97.2	4.2	90.4	3.0	90.0	11.7	87.0	14.5
244	Spiromesifen	0.001	0.003	0.9994	203.2	110.0	4.1	88.9	7.6	75.3	3.2	104.6	11.4	103.5	13.4	101.8	11.6
245	Spiroxamine-1	0.002	0.006	0.9974	181.5	103.1	15.2	91.6	4.1	97.7	6.0	93.9	4.4	93.7	9.0	92.7	14.3
	Spiroxamine-2	0.001	0.002	0.9946	149.4	114.3	9.9	107.3	6.6	97.5	3.0	98.4	11.0	95.8	11.4	93.9	15.9
246	Sulfotep	0.001	0.002	0.9983	112.2	112.0	5.1	111.0	1.5	108.6	4.5	92.4	9.2	89.6	18.2	85.5	15.9
247	Tebuconazole	0.001	0.003	0.9997	406.9	113.9	2.7	105.4	2.0	82.8	3.2	97.9	0.9	95.2	4.7	93.9	2.0

No.	Analyte	LOD	LOQ	R^2	ME.(%)	Repeatability (*n*=5)						Reproducibility (*n*=11)					
						0.01 mg kg^{-1}		0.02 mg kg^{-1}		0.1 mg kg^{-1}		0.01 mg kg^{-1}		0.02 mg kg^{-1}		0.1 mg kg^{-1}	
						Rec. (%)	CV (%)	Rec. (%)	CV (%)	Rec. (%)	CV (%)	Rec. (%)	CV (%)	Rec. (%)	CV (%)	Rec. (%)	CV (%)
248	Tebufenpyrad	0.001	0.002	0.9995	217.1	117.8	3.5	98.5	2.2	82.8	2.7	98.4	4.6	97.2	8.2	92.2	3.8
249	Tebupirimfos	0.001	0.003	0.9991	106.8	111.9	5.4	104.0	2.8	102.0	3.2	93.4	5.4	90.1	12.2	86.2	12.0
250	Tecnazene	0.001	0.004	0.9978	98.8	97.0	4.8	105.3	3.7	107.1	4.6	99.8	5.3	93.5	15.1	97.2	3.9
251	Tefluthrin	0.001	0.002	0.9981	71.0	110.1	5.1	103.9	4.9	105.3	3.6	96.4	2.8	95.6	8.7	87.8	11.6
252	Terbacil	0.001	0.003	0.9995	387.5	112.4	5.1	114.5	1.8	94.9	4.7	94.0	15.1	86.5	18.6	89.9	10.8
253	Terbumeton	0.001	0.002	0.9989	132.8	108.4	5.5	107.8	2.1	99.3	4.6	89.8	7.9	87.8	17.2	91.9	3.3
254	Terbutryn	0.003	0.009	0.9993	127.6	113.7	4.6	110.3	2.9	97.2	3.9	95.0	3.4	92.0	9.9	87.6	9.0
255	Tetrachlorvinphos	0.002	0.005	0.9992	421.5	108.6	8.0	114.8	4.8	78.3	2.0	92.7	8.0	87.6	15.1	87.2	0.4
256	Tetraconazole	0.001	0.003	0.9995	119.8	116.8	0.9	112.1	1.7	96.8	3.4	97.5	2.6	96.1	11.1	91.9	4.6
257	Tetradifon	0.001	0.002	0.9998	102.7	117.4	4.0	105.7	2.2	93.7	1.1	96.3	6.0	97.6	13.0	90.9	8.8
258	Tetramethrin-1	0.001	0.003	0.9997	>500	115.5	6.3	77.7	6.3	70.0	3.7	102.3	8.2	100.3	13.9	97.2	6.2
	Tetramethrin-2	0.001	0.004	0.9997	399.9	114.3	3.7	106.9	2.9	73.4	3.6	117.9	13.1	127.7	20.9	133.6	29.4
259	Thifluzamide	0.001	0.002	0.9997	427.9	116.8	3.3	110.3	2.5	80.8	4.5	100.2	3.1	97.4	8.8	95.6	5.2
260	Thiometon	0.001	0.004	0.9995	152.0	109.7	0.9	103.7	3.6	102.2	6.5	86.6	10.5	88.8	11.5	92.4	3.1
261	Thionazin	0.001	0.003	0.9983	116.4	111.7	0.7	102.3	1.8	106.8	5.9	88.3	10.1	88.1	8.8	91.3	3.6
262	Tolclofos-methyl	0.001	0.002	0.9986	93.1	112.9	4.1	106.1	2.8	99.4	3.5	90.1	11.4	93.2	10.0	92.9	3.9
263	Triadimefon	0.001	0.002	0.9994	110.5	115.6	2.7	112.6	2.1	97.8	4.2	97.8	2.3	95.3	12.3	94.3	8.9
264	Triadimenol	0.001	0.003	0.9998	267.3	113.7	3.9	110.2	1.1	94.5	4.2	99.9	5.2	93.6	7.8	96.5	3.8
265	Tri-allate	0.001	0.003	0.9989	90.3	104.8	6.3	105.8	3.4	104.1	2.9	94.2	3.2	90.5	11.5	84.8	13.0
266	Triazophos	0.001	0.003	0.9994	374.3	114.1	5.5	100.7	3.7	82.0	3.3	100.7	21.1	93.5	24.7	93.1	16.1
267	Tridiphane	0.003	0.008	0.9988	70.4	107.1	6.0	96.2	4.3	86.0	4.9	90.8	6.1	86.9	16.2	87.3	17.7
268	Trifloxystrobin	0.001	0.002	0.9996	158.5	116.9	2.2	103.5	2.6	82.3	2.9	98.0	5.8	96.5	10.6	96.1	1.7
269	Triflumizole	0.001	0.003	0.9997	142.4	111.5	4.1	104.5	1.2	88.0	3.4	96.7	3.3	92.2	10.0	90.4	5.9

No.	Analyte	LOD	LOQ	R^2	ME.(%)	Repeatability (*n*=5)						Reproducibility (*n*=11)					
						0.01 mg kg^{-1}		0.02 mg kg^{-1}		0.1 mg kg^{-1}		0.01 mg kg^{-1}		0.02 mg kg^{-1}		0.1 mg kg^{-1}	
						Rec. (%)	CV (%)	Rec. (%)	CV (%)	Rec. (%)	CV (%)	Rec. (%)	CV (%)	Rec. (%)	CV (%)	Rec. (%)	CV (%)
270	Trifluralin	0.001	0.004	0.9980	133.4	107.8	7.4	111.6	3.8	111.4	5.7	101.7	1.8	95.2	8.3	92.8	7.3
271	Vinclozolin	0.001	0.002	0.9999	73.6	109.4	4.8	111.3	2.7	103.3	3.7	93.9	6.8	89.5	15.8	92.8	5.0
272	Zoxamide	0.002	0.005	0.9993	>500	104.3	5.3	106.5	5.0	72.1	1.4	98.8	4.4	94.7	2.0	87.8	6.5

Table S6. Method validation parameter of 272 pesticides in green pepper.

No.	Analyte	LOD	LOQ	R^2	ME.(%)	Repeatability (*n*=5)						Reproducibility (*n*=11)					
						0.01 mg kg^{-1}		0.02 mg kg^{-1}		0.1 mg kg^{-1}		0.01 mg kg^{-1}		0.02 mg kg^{-1}		0.1 mg kg^{-1}	
						Rec. (%)	CV (%)	Rec. (%)	CV (%)	Rec. (%)	CV (%)	Rec. (%)	CV (%)	Rec. (%)	CV (%)	Rec. (%)	CV (%)
1	2,6-DIPN	0.003	0.008	0.9974	95.7	122.7	10.8	166.2	12.1	96.4	8.4	142.7	6.9	108.7	4.9	91.9	5.1
2	Acetochlor	0.001	0.004	0.9995	79.6	101.6	3.0	111.1	2.5	105.8	5.0	96.8	3.1	98.2	5.7	96.2	1.8
	EMA	0.003	0.008	0.9977	38.5	56.1	25.3	105.8	9.8	89.3	18.1	91.7	25.1	91.3	22.5	87.2	8.6
	HEMA	0.002	0.005	0.9995	454.9	35.2	9.7	81.1	10.3	64.1	6.3	37.6	46.5	47.2	60.1	47.2	60.8
3	Acrinathrin	0.001	0.004	0.9992	>500	450.8	7.7	96.2	4.3	89.9	1.5	60.3	92.0	83.9	21.8	93.7	12.0
4	Alachlor	0.001	0.003	0.9992	70.4	99.4	3.1	111.7	1.7	105.4	4.3	93.9	6.6	95.5	1.4	93.5	5.0
5	Aldrin	0.001	0.003	0.9981	54.1	84.8	3.1	101.6	1.7	101.8	5.5	90.4	0.2	89.7	5.5	88.9	9.0
	Dieldrin	0.001	0.004	0.9999	32.6	90.2	2.3	116.7	2.4	107.6	3.3	88.4	12.2	92.7	7.5	91.0	5.4
6	Allidochlor	0.002	0.007	0.9983	73.4	104.1	5.3	118.1	2.3	97.6	13.5	96.4	6.7	98.6	7.3	100.1	7.6
7	Ametryn	0.001	0.002	0.9993	110.0	88.2	2.9	104.0	2.9	100.2	3.1	94.5	6.8	92.9	4.3	91.6	3.1
8	Anilofos	0.001	0.004	0.9994	>500	103.2	8.7	93.4	4.6	83.9	2.1	84.5	10.1	87.4	8.3	83.1	4.9
9	Aramite	0.001	0.004	0.9999	112.4	114.6	2.9	111.2	3.8	108.0	1.1	91.7	11.3	95.3	0.6	94.3	2.1
10	Aspon	0.001	0.004	0.9995	107.5	104.4	3.1	116.2	1.9	110.0	3.8	92.3	6.9	93.9	5.0	92.5	3.9
11	Atrazine	0.001	0.004	0.9986	112.1	95.7	2.5	111.3	1.4	104.8	5.4	93.9	7.4	91.1	3.8	92.5	4.3
12	Azaconazole	0.001	0.003	1.0000	61.1	101.0	3.2	112.8	4.1	108.8	1.8	93.4	7.0	94.7	3.7	93.4	3.8
13	Benfluralin	0.001	0.003	0.9990	52.5	99.9	2.9	108.0	4.1	102.7	9.1	102.4	5.5	96.0	3.6	99.4	3.2
14	Benfuresate	0.001	0.002	0.9995	47.3	90.1	2.4	114.8	0.3	108.3	3.8	92.7	8.3	94.6	3.7	93.8	4.2
15	Benodanil	0.001	0.003	0.9998	182.9	116.0	4.2	111.1	5.3	106.4	1.7	90.6	11.6	93.1	3.2	92.4	3.6
16	Benzoylprop-ethyl	0.002	0.005	0.9999	91.6	91.4	2.5	112.5	5.0	105.8	1.9	104.7	4.2	99.2	4.6	92.1	4.1
17	BHC, *α*-	0.002	0.005	0.9997	48.8	118.5	2.8	119.9	4.2	104.4	6.8	95.4	16.1	93.9	12.5	88.3	9.3
	BHC, *β*-	0.001	0.002	0.9992	36.1	90.0	6.0	115.5	1.4	106.3	3.9	92.6	7.7	88.2	2.0	92.4	19.2

No.	Analyte	LOD	LOQ	R^2	ME.(%)	Repeatability (n=5)						Reproducibility (n=11)					
						0.01 mg kg^{-1}		0.02 mg kg^{-1}		0.1 mg kg^{-1}		0.01 mg kg^{-1}		0.02 mg kg^{-1}		0.1 mg kg^{-1}	
						Rec. (%)	CV (%)	Rec. (%)	CV (%)	Rec. (%)	CV (%)	Rec. (%)	CV (%)	Rec. (%)	CV (%)	Rec. (%)	CV (%)
	BHC, *δ*-	0.001	0.004	0.9999	38.3	119.8	3.6	106.0	4.1	95.0	3.7	84.0	8.7	89.2	7.7	85.7	5.8
18	BHC, *γ*-	0.002	0.005	0.9995	36.1	115.0	8.6	115.1	1.8	101.7	9.4	95.2	22.2	89.5	10.9	92.7	22.6
19	Bifenox	0.001	0.003	0.9994	207.4	183.9	11.4	108.4	4.7	102.4	0.7	105.6	5.6	102.0	5.8	94.4	7.0
20	Bifenthrin	0.001	0.004	1.0000	145.5	89.3	2.1	108.3	5.3	102.5	1.7	92.1	6.5	93.7	6.4	91.6	5.6
21	Boscalid	0.001	0.004	0.9998	322.1	106.0	4.1	107.8	5.8	104.0	0.9	90.1	8.4	91.5	3.6	90.6	0.7
22	Bromobutide	0.001	0.003	0.9985	54.5	85.9	4.3	107.0	4.0	105.1	4.9	89.0	2.4	92.0	5.2	95.1	9.5
23	Bromophos-methyl	0.001	0.003	0.9998	92.5	114.6	4.0	109.2	3.6	105.6	2.2	86.0	9.0	89.5	1.3	86.9	3.9
24	Bromophos-ethyl	0.001	0.004	0.9995	93.7	100.2	3.1	108.7	1.5	104.5	2.8	89.7	5.9	95.2	0.3	90.2	5.1
25	Bromopropylate	0.001	0.004	0.9999	264.3	107.3	2.8	109.5	4.5	103.1	1.5	92.7	7.6	94.1	2.1	91.5	4.5
26	Bupirimate	0.001	0.003	0.9998	94.6	105.4	3.4	110.9	4.3	106.5	1.7	95.1	8.6	93.6	5.6	91.7	5.4
27	Buprofezin	0.001	0.004	0.9998	61.3	101.2	3.1	111.3	4.2	106.7	1.7	94.6	10.5	98.9	2.3	91.5	5.3
28	Butachlor	0.002	0.007	0.9997	102.1	109.2	1.2	110.3	1.7	103.9	2.8	90.1	2.5	93.8	2.5	89.3	5.2
29	Butafenacil	0.001	0.004	0.9990	444.0	103.0	3.9	106.1	7.6	103.4	1.7	90.8	4.9	90.4	5.0	92.6	1.1
30	Butralin	0.001	0.003	0.9985	80.8	119.9	8.0	110.4	2.2	103.7	3.3	97.1	3.7	92.1	5.2	97.8	3.0
31	Butylate	0.002	0.006	0.9982	94.6	90.7	4.3	98.7	3.9	96.7	15.0	95.1	4.2	92.7	3.5	94.6	10.7
32	Cadusafos	0.002	0.005	0.9986	170.1	90.8	2.4	111.5	6.5	108.4	7.9	94.7	6.2	94.1	5.5	94.2	6.7
33	Carbophenothion	0.001	0.004	0.9998	173.0	101.3	4.2	104.6	6.4	99.9	1.5	89.9	12.8	90.8	1.4	91.0	1.7
34	Carboxin	0.003	0.009	0.9997	37.1	75.1	20.1	104.4	3.0	87.8	1.9	76.9	10.6	78.4	7.5	84.8	1.2
35	Carfentrazone-ethyl	0.001	0.003	0.9998	136.1	124.2	7.4	107.4	4.3	103.9	1.5	45.1	139.7	94.6	16.7	76.3	2.9
36	Chinomethionat	0.001	0.004	0.9999	86.5	299.9	7.0	96.1	2.3	87.3	0.7	75.3	23.9	74.5	32.0	79.8	11.4
37	Chlorbenside	0.001	0.003	1.0000	91.9	83.5	2.6	111.7	2.7	105.4	2.1	86.6	6.0	90.4	3.1	88.3	3.1
38	Chlorbufam	0.002	0.005	0.9998	199.6	116.4	8.9	108.3	1.6	101.7	3.2	92.0	19.8	87.0	13.4	83.3	7.6
39	Chlordane-*cis*	0.001	0.003	0.9999	46.0	86.8	4.0	113.9	2.1	108.8	3.2	95.2	4.2	95.5	4.4	90.9	6.9

No.	Analyte	LOD	LOQ	R^2	ME.(%)	Repeatability (*n*=5)						Reproducibility (*n*=11)					
						0.01 mg kg^{-1}		0.02 mg kg^{-1}		0.1 mg kg^{-1}		0.01 mg kg^{-1}		0.02 mg kg^{-1}		0.1 mg kg^{-1}	
						Rec. (%)	CV (%)	Rec. (%)	CV (%)	Rec. (%)	CV (%)	Rec. (%)	CV (%)	Rec. (%)	CV (%)	Rec. (%)	CV (%)
	Chlordane-*trans*	0.001	0.003	0.9999	41.7	89.9	3.5	113.8	2.7	106.6	3.1	95.7	7.9	95.4	7.3	90.9	7.0
40	Chlorethoxyfos	0.001	0.004	0.9978	55.2	87.0	9.0	113.7	1.3	99.9	12.9	92.6	6.2	103.9	5.4	102.3	6.7
41	Chlorfenapyr	0.001	0.003	1.0000	90.0	102.8	5.7	114.7	5.6	108.1	1.4	89.1	5.6	92.3	10.6	94.3	5.1
42	Chlorfenson	0.001	0.003	0.9999	71.2	108.7	2.8	116.6	4.0	110.2	2.4	91.9	7.0	94.8	3.3	90.4	3.1
43	Chlorflurenol-methyl	0.001	0.004	0.9999	205.0	115.7	10.9	108.6	3.2	104.7	1.7	90.3	7.0	92.1	2.9	92.3	3.5
44	Chlornitrofen	0.001	0.003	0.9996	120.3	114.3	4.2	110.6	3.2	105.7	1.8	97.0	3.7	93.9	8.4	92.9	2.6
45	Chlorobenzilate	0.001	0.004	0.9999	104.4	102.3	2.3	109.3	3.7	108.4	1.8	91.9	7.6	94.7	5.0	92.0	5.3
46	Chloropropylate	0.002	0.005	0.9998	101.8	102.3	2.3	109.9	4.2	108.4	2.2	94.0	7.2	96.7	1.3	93.7	2.0
47	Chloroneb	0.001	0.004	0.9985	74.2	91.4	2.3	104.7	6.0	105.0	12.4	92.5	16.1	93.7	14.0	96.1	10.8
48	Chlorothalonil	0.003	0.009	0.9984	221.3	333.4	23.5	80.1	10.4	58.3	3.5	63.0	40.9	66.8	52.7	91.3	29.1
49	Chlorpropham	0.002	0.005	0.9987	133.4	94.3	3.5	113.2	3.5	104.9	10.2	88.0	10.5	89.3	4.6	92.9	8.3
50	Chlorpyrifos	0.001	0.004	0.9990	79.8	107.8	3.6	115.8	2.3	108.9	3.8	90.1	4.6	92.6	2.0	91.2	3.3
51	Chlorpyrifos-methyl	0.002	0.005	0.9979	96.1	115.5	4.0	107.8	1.9	101.1	4.9	87.6	6.2	91.3	1.7	88.8	2.0
52	Chlorthal-dimethyl	0.001	0.003	0.9991	50.5	94.7	3.0	117.3	1.9	109.2	4.0	92.5	7.8	90.3	11.9	90.2	8.0
53	Chlorthion	0.001	0.004	0.9988	341.9	114.1	2.6	103.2	1.7	95.9	3.9	83.6	10.0	89.1	15.6	87.7	7.6
54	Chlorthiophos	0.001	0.004	1.0000	108.0	98.3	3.7	112.1	3.9	106.2	1.4	92.2	10.3	94.1	1.0	91.0	3.7
55	Chlozolinate	0.001	0.003	0.9996	50.1	116.0	7.6	108.8	2.1	104.8	2.1	88.4	8.0	91.2	1.5	89.7	1.7
56	Cinmethylin	0.001	0.003	0.9970	41.1	88.5	3.8	110.2	7.7	99.5	4.9	96.9	11.0	98.9	13.3	94.9	3.9
57	Clomazone	0.001	0.004	0.9999	67.6	89.9	3.7	115.5	1.6	107.7	6.6	98.3	10.3	98.6	4.0	97.1	6.7
58	Coumaphos	0.002	0.005	0.9993	>500	103.4	8.6	98.0	4.9	91.4	0.4	80.5	21.9	81.5	14.0	90.3	1.8
59	Cyanophos	0.001	0.004	0.9992	137.4	107.0	4.2	111.8	2.0	104.4	4.9	88.1	10.8	90.0	1.1	89.3	2.4
60	Cyflufenamid	0.001	0.003	0.9999	144.7	113.2	3.2	113.1	4.0	108.8	1.3	94.9	5.5	95.6	4.8	93.1	5.9
61	Cyfluthrin	0.001	0.004	0.9997	421.1	368.3	6.4	106.1	4.7	101.5	3.4	90.1	10.5	88.5	6.1	91.2	6.5

No.	Analyte	LOD	LOQ	R^2	ME.(%)	Repeatability (n=5)						Reproducibility (n=11)					
						0.01 mg kg^{-1}		0.02 mg kg^{-1}		0.1 mg kg^{-1}		0.01 mg kg^{-1}		0.02 mg kg^{-1}		0.1 mg kg^{-1}	
						Rec. (%)	CV (%)	Rec. (%)	CV (%)	Rec. (%)	CV (%)	Rec. (%)	CV (%)	Rec. (%)	CV (%)	Rec. (%)	CV (%)
62	Cyhalofop-butyl	0.001	0.004	0.9997	296.0	94.6	3.3	103.8	7.8	95.5	1.7	92.5	11.1	95.4	2.2	93.2	1.0
63	Cyhalothrin, *γ*-	0.001	0.004	0.9998	323.5	202.7	5.7	95.6	5.8	95.0	0.9	64.9	85.9	89.2	23.2	97.6	4.9
	Cyhalothrin, *λ*-	0.002	0.005	0.9995	429.8	314.2	5.1	80.5	9.2	105.2	1.3	57.4	86.5	83.6	19.0	91.5	2.7
64	Cypermethrin	0.001	0.004	0.9997	346.2	365.4	5.9	97.8	4.7	96.2	1.1	91.6	10.5	90.4	9.3	90.6	6.2
65	Cyprazine	0.001	0.004	0.9992	77.2	99.7	2.8	108.8	1.8	104.8	3.6	89.1	8.5	89.7	5.0	91.3	1.8
66	Cyprodinil	0.001	0.004	0.9998	79.3	93.4	2.8	108.9	3.0	103.9	2.3	91.4	6.3	92.1	2.9	89.1	4.8
67	DDD, *p,p'*-	0.001	0.003	1.0000	51.5	108.7	4.3	108.2	3.8	102.7	2.2	93.5	12.4	95.2	9.6	92.2	9.1
	DDE, *p,p'*-	0.001	0.003	1.0000	46.8	78.9	2.4	108.0	4.3	103.4	2.8	88.3	6.1	90.9	7.9	86.9	7.0
	DDT, *o,p'*-	0.001	0.003	0.9997	3.3	117.9	15.9	102.3	5.9	100.8	2.3	94.8	33.3	76.8	14.5	68.5	18.0
	DDT, *p,p'*-	0.001	0.003	0.9992	17.5	134.8	12.9	98.3	9.3	95.3	3.2	92.1	54.3	77.3	22.0	56.7	26.2
68	Deltamethrin	0.001	0.002	0.9996	>500	99.8	3.5	86.8	3.3	90.7	1.3	101.9	8.5	96.0	5.8	99.7	6.9
69	Desmetryn	0.001	0.004	0.9992	90.8	88.0	3.1	108.6	0.7	103.7	3.9	95.8	5.9	97.8	7.6	96.1	3.4
70	Dialifor	0.001	0.003	0.9993	266.8	116.9	8.7	102.6	4.4	95.8	0.5	85.1	19.0	87.4	12.6	86.9	8.0
71	Di-Allate	0.002	0.005	0.9986	76.8	86.4	3.3	116.1	2.9	100.4	9.3	93.0	8.8	95.4	5.4	94.6	8.8
72	Diazinon	0.003	0.008	0.9985	50.2	91.1	2.3	110.5	2.0	107.3	6.7	94.5	6.1	93.2	5.6	94.9	6.9
73	Dichlobenil	0.002	0.006	0.9984	48.8	89.6	2.4	105.8	9.6	103.1	14.7	94.2	5.8	98.0	7.8	97.9	10.1
74	Dichlofenthion	0.001	0.004	0.9961	72.9	88.2	2.1	110.4	2.2	104.4	6.9	92.3	5.7	94.4	4.3	92.1	6.6
75	Dichlofluanid	0.002	0.006	0.9546	227.5	55.2	8.5	471.3	2.2	26.4	18.3	120.1	68.2	118.9	38.8	124.5	52.8
76	Dichlormid	0.002	0.006	0.9997	38.2	98.4	6.7	118.2	1.6	100.2	13.7	100.5	9.1	105.6	11.3	104.2	9.5
77	Diclobutrazol	0.002	0.005	0.9996	394.1	116.0	3.5	109.0	4.7	105.4	1.1	91.2	11.1	93.6	4.1	92.6	5.7
78	Diclofop-methyl	0.001	0.003	0.9998	133.8	100.4	3.4	103.8	5.2	99.2	1.9	92.2	8.4	94.3	2.6	90.9	4.2
79	Dicloran	0.001	0.004	0.9994	125.6	113.6	8.4	115.1	2.7	106.4	4.7	97.7	4.0	96.4	5.5	95.0	4.1
80	Dicofol	0.001	0.003	0.9999	51.2	62.8	7.4	115.1	2.7	110.4	2.1	86.0	16.6	85.8	13.5	94.8	9.5

No.	Analyte	LOD	LOQ	R^2	ME.(%)	Repeatability (*n*=5)						Reproducibility (*n*=11)					
						0.01 mg kg^{-1}		0.02 mg kg^{-1}		0.1 mg kg^{-1}		0.01 mg kg^{-1}		0.02 mg kg^{-1}		0.1 mg kg^{-1}	
						Rec. (%)	CV (%)	Rec. (%)	CV (%)	Rec. (%)	CV (%)	Rec. (%)	CV (%)	Rec. (%)	CV (%)	Rec. (%)	CV (%)
81	Dicrotophos	0.002	0.007	0.9984	>500	105.3	13.2	92.9	4.8	85.3	5.5	103.6	20.2	88.1	18.4	87.2	18.1
82	Diethatyl-ethyl	0.001	0.003	0.9999	107.2	115.1	3.1	116.0	3.8	110.3	2.3	98.2	9.3	99.9	4.4	94.8	3.2
83	Diethofencarb	0.002	0.005	0.9993	285.2	118.4	8.8	112.2	3.0	107.2	2.5	80.7	16.5	88.8	6.0	92.0	3.8
84	Difenoconazole	0.001	0.002	0.9996	404.9	96.7	3.5	104.3	5.4	101.3	0.5	91.5	10.0	92.0	7.1	93.1	7.0
85	Diflufenican	0.001	0.004	0.9999	254.8	98.6	3.2	107.3	4.8	101.4	1.1	88.1	11.7	91.6	4.1	89.3	8.0
86	Dimepiperate	0.001	0.004	0.9995	92.4	101.3	3.1	109.6	3.0	104.3	2.8	95.1	7.7	95.9	7.1	91.8	4.3
87	Dimethachlor	0.003	0.009	0.9986	63.4	100.2	2.7	110.6	1.5	105.5	4.4	88.6	2.0	92.1	4.3	94.0	4.9
88	Dimethametryn	0.001	0.003	0.9995	85.0	101.8	1.9	108.2	3.1	103.8	2.2	92.1	6.7	91.6	3.6	89.9	4.5
89	Dimethenamid	0.001	0.004	0.9973	61.6	97.3	2.9	110.2	1.4	105.1	5.6	92.7	6.7	93.4	3.8	92.6	6.2
90	Dimethipin	0.002	0.005	0.9999	92.2	335.6	14.7	111.1	3.4	97.3	3.3	83.1	14.4	86.4	1.5	85.9	7.4
91	Dimethomorph (*E*)	0.002	0.005	0.9988	>500	95.7	4.8	96.6	6.3	100.1	0.8	91.0	7.4	92.2	3.5	88.7	8.1
	Dimethomorph (*Z*)	0.002	0.005	0.9998	>500	82.4	6.4	104.2	7.3	104.8	0.9	90.5	13.5	93.1	4.7	89.8	9.1
92	Dimethylvinphos (*E*)	0.002	0.005	0.9992	262.7	106.8	12.4	98.5	3.1	90.0	2.1	80.0	11.5	86.7	9.8	82.8	16.2
	Dimethylvinphos (*Z*)	0.001	0.004	0.9997	351.3	116.1	11.7	103.1	2.4	91.2	1.5	79.1	9.2	85.6	13.9	82.2	17.1
93	Diniconazole	0.002	0.005	0.9994	425.4	111.4	2.9	108.0	4.6	103.2	2.1	93.2	11.0	93.5	1.4	92.5	5.0
94	Dinitramine	0.001	0.004	0.9958	109.6	103.2	4.9	106.7	2.6	102.2	6.5	97.0	5.4	92.5	4.4	97.5	3.7
95	Dioxathion	0.002	0.006	0.9999	66.7	90.5	4.3	113.3	1.3	108.6	0.8	87.4	11.2	92.1	4.3	90.5	4.6
96	Diphenamid	0.001	0.003	0.9998	55.5	99.8	2.3	114.9	1.7	108.1	2.6	90.7	3.6	93.1	3.1	92.6	3.8
97	Diphenylamine	0.002	0.006	0.9966	107.4	90.3	2.5	117.2	1.5	103.2	8.9	93.0	3.8	91.9	4.6	90.6	6.0
98	Dithiopyr	0.001	0.003	0.9982	59.8	89.1	1.9	111.9	1.0	106.5	4.4	96.5	4.6	95.2	5.0	92.8	7.7
99	Edifenphos	0.002	0.005	0.9998	>500	196.0	10.6	88.7	6.1	76.0	2.5	68.7	32.9	85.0	27.5	67.2	18.6
100	Endosulfan, *α*-	0.001	0.003	0.9998	36.8	93.9	3.8	113.9	3.3	109.0	3.2	89.2	11.2	94.6	6.1	89.2	7.1
	Endosulfan, *β*-	0.001	0.003	1.0000	27.4	110.3	6.7	112.3	2.9	108.4	2.8	96.5	10.1	97.7	8.2	92.0	7.7

No.	Analyte	LOD	LOQ	R^2	ME.(%)	Repeatability (n=5)						Reproducibility (n=11)					
						0.01 mg kg^{-1}		0.02 mg kg^{-1}		0.1 mg kg^{-1}		0.01 mg kg^{-1}		0.02 mg kg^{-1}		0.1 mg kg^{-1}	
						Rec. (%)	CV (%)	Rec. (%)	CV (%)	Rec. (%)	CV (%)	Rec. (%)	CV (%)	Rec. (%)	CV (%)	Rec. (%)	CV (%)
	Endosulfan, sulfate-	0.001	0.003	1.0000	63.9	91.4	2.7	106.6	5.4	100.6	2.2	75.0	8.9	83.6	9.4	89.1	11.3
101	Endrin	0.001	0.003	0.9999	57.7	90.9	4.3	112.9	2.4	107.9	2.7	95.0	13.4	97.3	13.5	90.9	12.1
	δ-keto-Endrin	0.001	0.004	0.9993	23.1	60.3	17.5	112.2	5.9	107.6	2.2	88.9	21.4	86.2	15.2	90.4	9.7
102	EPN	0.001	0.004	0.9995	259.7	119.8	5.3	111.3	3.6	101.0	1.6	93.9	3.3	92.1	10.6	95.2	3.8
103	Epoxiconazole	0.001	0.003	0.9999	165.0	99.5	5.0	112.3	5.0	104.8	1.3	95.5	9.4	94.7	8.1	92.5	8.2
104	EPTC	0.003	0.009	0.9991	35.2	85.8	4.5	110.6	4.8	99.0	14.7	97.8	4.7	103.0	5.4	101.6	3.6
105	Etaconazole-1	0.001	0.003	1.0000	88.8	98.3	1.0	114.4	5.1	111.7	1.6	93.2	9.1	100.1	9.0	97.6	3.7
	Etaconazole-2	0.001	0.003	0.9998	63.3	97.6	4.0	109.2	9.9	115.9	2.0	95.9	0.8	97.0	2.4	96.1	0.3
106	Ethalfluralin	0.001	0.004	0.9996	67.1	99.4	2.8	111.2	3.1	99.7	11.2	98.7	3.6	94.3	3.0	97.5	5.6
107	Ethion	0.001	0.003	0.9996	137.9	109.3	3.7	109.4	4.2	105.4	1.4	90.4	11.6	94.0	2.1	94.6	0.7
108	Ethofumesate	0.001	0.003	0.9993	66.5	90.1	2.4	109.0	2.2	105.2	2.7	95.8	7.8	92.8	5.0	91.7	3.8
109	Ethoprophos	0.002	0.005	0.9955	172.0	98.3	3.8	117.9	3.1	102.6	6.1	94.9	6.2	91.5	6.3	95.9	6.6
110	Ethychlozate	0.001	0.004	0.9995	257.0	117.9	8.7	92.6	3.1	90.9	1.3	80.7	18.7	83.0	10.3	91.8	8.3
111	Etoxazole	0.001	0.004	0.9998	165.0	78.0	1.7	109.4	4.2	102.9	1.0	94.5	3.8	92.0	3.7	90.5	6.0
112	Etridiazole	0.002	0.005	0.9937	48.4	99.1	15.1	103.3	6.3	86.9	14.6	87.2	19.9	82.6	11.1	87.2	14.0
113	Fenamidone	0.001	0.003	0.9998	123.6	86.7	2.7	111.7	4.0	105.4	1.4	92.3	7.0	92.3	3.4	92.3	3.5
114	Fenarimol	0.001	0.004	0.9998	212.6	92.8	2.8	108.6	5.2	102.9	1.5	99.4	13.6	97.1	6.1	92.9	0.3
115	Fenbuconazole	0.001	0.004	0.9998	308.4	90.2	4.3	110.6	5.7	105.2	1.2	90.3	10.0	95.0	3.8	91.9	3.7
116	Fenchlorphos	0.002	0.005	0.9996	79.7	99.5	3.0	108.5	1.3	101.6	3.9	88.7	9.5	91.2	2.5	88.8	3.0
117	Fenclorim	0.001	0.004	0.9953	106.1	94.3	3.1	111.6	2.3	98.6	10.5	91.6	4.6	91.7	7.3	89.5	7.9
118	Fenfuram	0.002	0.006	0.9996	-10.9	94.9	10.4	96.7	2.7	74.6	4.2	73.6	18.5	83.0	19.6	85.8	20.2
119	Fenitrothion	0.002	0.005	0.9989	209.6	119.3	3.5	104.4	3.2	99.9	3.2	86.0	6.8	87.7	3.3	86.5	8.6
120	Fenobucarb	0.002	0.007	0.9998	143.0	118.8	3.3	115.7	2.1	100.5	10.0	90.9	5.8	92.0	4.0	90.9	6.1

No.	Analyte	LOD	LOQ	R^2	ME.(%)	Repeatability (n=5)						Reproducibility (n=11)					
						0.01 mg kg^{-1}		0.02 mg kg^{-1}		0.1 mg kg^{-1}		0.01 mg kg^{-1}		0.02 mg kg^{-1}		0.1 mg kg^{-1}	
						Rec. (%)	CV (%)	Rec. (%)	CV (%)	Rec. (%)	CV (%)	Rec. (%)	CV (%)	Rec. (%)	CV (%)	Rec. (%)	CV (%)
121	Fenothiocarb	0.001	0.003	0.9999	171.4	95.4	3.0	115.0	3.8	109.7	1.9	92.2	6.8	94.9	2.5	92.6	1.1
122	Fenoxanil	0.001	0.004	0.9999	443.8	119.2	4.5	111.0	4.9	105.1	1.5	89.7	11.1	93.8	1.4	88.9	2.6
123	Fenpropathrin	0.001	0.003	0.9998	133.9	113.1	9.6	110.1	5.5	102.8	1.8	96.6	6.1	93.9	0.9	94.2	2.0
124	Fenpropimorph	0.001	0.003	0.9975	80.5	94.7	2.9	108.3	2.1	104.0	3.2	93.8	1.1	93.4	2.0	92.0	1.0
125	Fenpyrazamine	0.001	0.004	0.9993	>500	118.2	10.2	100.5	5.3	89.7	0.8	83.8	11.1	85.2	5.4	85.3	4.2
126	Fenson	0.001	0.003	0.9998	40.6	105.7	3.3	114.8	2.7	108.6	2.8	93.7	5.8	93.7	3.5	92.1	3.5
127	Fenthion	0.002	0.007	0.9992	99.7	102.2	4.1	112.1	1.8	105.6	2.7	89.2	5.9	88.9	4.7	88.6	2.6
128	Fenvalerate-1	0.001	0.004	0.9998	347.6	144.8	13.6	96.7	4.4	96.0	0.5	93.3	6.6	91.4	12.7	95.6	7.0
	Fenvalerate-2	0.001	0.004	0.9997	>500	138.1	11.8	102.5	6.8	100.0	0.2	93.1	5.9	91.9	12.8	96.9	4.9
129	Fipronil	0.001	0.003	0.9993	225.6	119.5	8.8	106.1	4.8	103.8	2.5	94.1	3.7	92.7	1.8	92.4	3.2
130	Flamprop-isopropyl	0.002	0.005	1.0000	68.4	97.3	2.3	110.9	4.5	107.3	1.6	99.7	8.1	100.7	4.6	96.6	2.8
131	Fluacrypyrim	0.001	0.004	0.9999	152.8	101.3	3.4	109.2	4.7	105.9	1.4	94.5	9.2	96.6	1.4	93.7	4.7
132	Fluazifop-butyl	0.001	0.003	0.9996	146.8	102.2	2.7	111.4	4.1	107.8	1.5	94.7	5.0	95.9	4.3	93.4	5.3
133	Fluchloralin	0.001	0.003	0.9991	59.0	119.5	3.9	111.7	2.4	104.3	7.0	100.3	5.3	96.0	2.7	98.3	5.3
134	Flucythrinate-1	0.001	0.004	0.9993	426.4	116.2	3.2	104.2	5.4	101.5	0.7	96.0	11.9	93.6	16.8	95.8	6.0
	Flucythrinate-2	0.001	0.004	0.9993	461.4	116.9	3.9	103.1	4.8	100.3	0.6	95.6	11.9	92.6	14.9	95.5	5.1
135	Fluensulfone	0.003	0.008	0.9994	117.3	103.5	2.0	117.4	2.8	106.2	9.6	95.5	6.0	92.4	5.2	92.6	9.2
136	Flufenpyr-ethyl	0.001	0.004	0.9991	171.1	118.4	6.3	107.0	3.6	103.2	1.4	94.2	13.3	97.3	5.1	96.7	3.8
137	Flumetralin	0.001	0.003	0.9983	98.3	117.4	5.4	111.6	2.3	107.1	3.1	102.2	4.2	98.5	6.0	94.7	2.4
138	Flumioxazin	0.001	0.004	0.9992	465.1	119.1	8.8	100.3	6.4	100.9	0.5	87.7	14.9	83.2	15.0	91.0	10.8
139	Fluopyram	0.001	0.004	0.9996	163.7	115.0	3.4	108.7	3.8	105.3	1.4	91.7	7.9	95.6	1.1	92.4	5.0
140	Fluorochloridone	0.001	0.004	0.9999	81.3	119.1	8.2	109.2	6.0	103.9	1.5	100.6	15.2	98.4	15.1	94.3	13.2
141	Fluquinconazole	0.001	0.003	0.9994	167.4	115.5	8.4	109.0	3.3	103.5	1.9	91.5	5.8	92.8	0.3	90.2	3.2

No.	Analyte	LOD	LOQ	R^2	ME.(%)	Repeatability (n=5)						Reproducibility (n=11)					
						0.01 mg kg^{-1}		0.02 mg kg^{-1}		0.1 mg kg^{-1}		0.01 mg kg^{-1}		0.02 mg kg^{-1}		0.1 mg kg^{-1}	
						Rec. (%)	CV (%)	Rec. (%)	CV (%)	Rec. (%)	CV (%)	Rec. (%)	CV (%)	Rec. (%)	CV (%)	Rec. (%)	CV (%)
142	Flusilazole	0.001	0.003	0.9999	91.8	102.7	3.6	111.0	4.2	107.7	1.8	92.7	8.0	93.8	3.7	91.8	5.5
143	Flutianil	0.001	0.004	0.9993	227.7	98.9	3.7	108.8	5.5	104.6	1.5	95.9	2.4	96.6	2.8	92.3	3.0
144	Fluvalinate-1	0.001	0.004	1.0000	>500	162.7	14.2	88.3	3.9	88.1	3.4	83.3	9.4	83.1	10.3	86.7	5.1
	Fluvalinate-2	0.001	0.004	0.9995	>500	168.1	13.7	88.1	3.7	87.0	4.1	85.6	7.5	82.6	9.4	86.9	2.6
145	Fluxapyroxad	0.001	0.003	0.9999	430.4	112.9	3.5	111.7	5.2	100.3	1.1	93.3	7.9	92.9	2.7	92.0	4.5
146	Fonofos	0.001	0.004	0.9992	51.9	89.3	1.6	116.9	1.9	105.2	9.1	94.2	6.6	94.2	4.8	93.1	6.5
147	Formothion	0.002	0.006	0.9995	347.8	117.2	7.2	101.0	1.2	90.7	3.0	81.1	15.5	85.0	7.2	79.9	14.2
148	Fthalide	0.001	0.003	0.9999	51.1	117.0	6.5	112.9	0.7	106.7	2.2	91.7	5.9	93.5	3.2	90.5	3.2
149	Halfenprox	0.001	0.003	0.9991	199.0	96.4	5.4	102.7	6.1	103.3	1.2	96.1	9.0	91.5	14.9	96.4	3.3
150	Heptachlor	0.001	0.004	0.9994	49.8	95.3	8.0	102.9	4.8	96.7	7.9	93.4	11.9	95.7	13.6	84.4	5.6
	Heptachlor epoxide	0.001	0.004	0.9998	19.7	92.9	3.1	113.9	1.2	111.5	5.2	98.9	3.4	96.3	3.1	93.9	6.1
151	Heptenophos	0.002	0.006	0.9994	166.9	115.0	9.7	115.1	2.8	98.0	11.3	90.9	5.0	92.1	2.0	90.5	2.8
152	Hexythiazox	0.002	0.005	0.9995	278.4	109.2	5.4	99.0	6.6	116.2	1.9	82.5	23.2	97.6	1.2	98.8	3.9
153	Indanofan	0.001	0.004	0.9999	245.6	88.7	7.3	111.1	4.9	103.8	1.1	89.7	7.0	96.3	2.4	91.4	7.6
154	Indoxacarb	0.001	0.003	0.9998	264.2	118.5	8.1	110.2	3.8	103.1	1.7	88.0	10.3	87.6	8.4	86.2	12.6
155	Ipconazole	0.002	0.005	0.9996	337.2	93.0	2.7	110.1	4.9	103.9	1.3	97.3	13.9	96.3	10.8	93.1	2.4
156	Iprobenfos	0.002	0.005	0.9987	249.7	103.8	2.5	101.9	6.9	101.0	5.5	92.3	6.7	92.7	2.7	93.8	5.8
157	Iprodione	0.001	0.004	0.9998	>500	114.1	7.4	91.8	10.9	78.0	1.6	78.2	11.2	85.1	10.4	76.4	8.2
158	Isazofos	0.001	0.004	0.9994	47.5	96.1	2.1	111.3	2.3	105.8	6.2	96.1	5.5	95.2	3.2	94.0	3.6
159	Isofenphos	0.001	0.004	0.9993	76.1	107.0	2.6	110.2	3.3	106.1	2.1	95.4	5.0	95.6	2.6	92.3	4.5
160	Isofenphos-methyl	0.001	0.003	0.9991	79.5	109.7	2.9	111.0	1.7	106.8	2.8	95.6	4.6	95.3	2.3	92.4	4.5
161	Isoprocarb	0.003	0.008	0.9996	151.8	116.6	5.2	115.7	1.2	99.3	11.7	89.4	9.2	89.5	5.2	89.9	2.6
162	Isopropalin	0.001	0.003	0.9992	70.3	112.6	3.0	109.9	2.2	104.2	3.4	94.9	4.7	92.3	3.0	95.9	2.9

No.	Analyte	LOD	LOQ	R^2	ME.(%)	Repeatability (*n*=5)						Reproducibility (*n*=11)					
						0.01 mg kg^{-1}		0.02 mg kg^{-1}		0.1 mg kg^{-1}		0.01 mg kg^{-1}		0.02 mg kg^{-1}		0.1 mg kg^{-1}	
						Rec. (%)	CV (%)	Rec. (%)	CV (%)	Rec. (%)	CV (%)	Rec. (%)	CV (%)	Rec. (%)	CV (%)	Rec. (%)	CV (%)
163	Isoprothiolane	0.001	0.003	1.0000	89.4	101.9	2.5	115.9	4.0	109.9	1.8	92.6	6.1	96.0	0.6	92.3	3.5
164	Isopyrazam	0.001	0.004	0.9994	418.4	101.8	3.9	106.1	5.4	100.3	0.7	94.1	7.0	93.8	1.0	93.6	1.5
165	Isotianil	0.001	0.004	0.9997	>500	119.4	8.9	113.6	4.6	102.8	1.3	90.1	12.9	90.9	2.1	91.8	3.5
166	Isoxadifen-ethyl	0.001	0.003	0.9999	108.0	115.7	8.7	108.3	4.4	104.9	1.5	93.3	5.7	91.5	3.0	92.5	5.1
167	Kresoxim-methyl	0.001	0.003	1.0000	55.5	109.3	2.8	114.9	4.4	109.5	1.9	94.4	6.2	96.6	3.4	92.1	5.3
168	Leptophos	0.001	0.004	0.9998	183.9	114.9	6.0	102.0	4.9	95.9	1.7	88.5	15.9	91.3	9.2	83.7	6.2
169	Mefenpyr-diethyl	0.001	0.003	0.9999	164.4	101.1	2.7	105.5	5.1	101.7	1.6	91.0	8.6	91.8	6.4	91.6	5.9
170	Mepanipyrim	0.001	0.004	1.0000	159.0	94.1	3.4	115.2	4.5	110.5	1.9	92.1	8.8	93.2	4.9	90.7	3.0
171	Mepronil	0.001	0.004	0.9999	293.6	97.7	4.0	107.9	5.8	104.3	0.9	86.0	6.8	90.7	5.3	91.9	4.9
172	Metalaxyl	0.001	0.004	0.9996	63.7	90.2	2.8	107.6	2.7	103.2	3.9	91.5	7.8	91.3	5.3	92.5	5.3
173	Methidathion	0.001	0.004	0.9999	290.7	107.2	7.6	105.0	2.6	96.0	1.2	84.4	6.4	85.4	5.7	79.2	6.0
174	Methoprotryn	0.001	0.003	0.9998	125.4	109.7	3.2	109.2	4.4	106.1	1.3	95.5	11.8	99.0	7.0	94.5	1.0
175	Methoxychlor	0.001	0.004	0.9992	93.8	97.5	19.1	104.6	7.1	102.0	2.7	87.2	28.1	76.9	11.5	84.8	13.6
176	Methyl trithion	0.001	0.004	0.9994	210.3	107.5	6.5	106.3	2.5	100.1	1.4	77.0	14.6	90.1	7.3	87.3	7.5
177	Metolachlor	0.001	0.004	0.9998	83.8	98.0	3.0	116.4	1.7	110.9	3.9	91.1	7.8	92.0	5.7	91.7	5.5
178	Metribuzin	0.001	0.004	0.9994	78.4	97.5	1.8	108.4	2.1	105.5	2.6	96.0	3.4	94.5	1.1	88.0	11.9
179	MGK-264	0.001	0.003	0.9997	63.2	97.0	2.1	109.9	2.5	105.4	2.5	98.4	8.0	99.0	7.0	95.5	3.8
180	Molinate	0.002	0.006	0.9999	40.7	92.6	2.5	115.7	3.7	102.4	12.2	60.6	86.9	113.2	32.0	124.2	42.4
181	Monolinuron	0.002	0.006	0.9994	167.5	102.3	6.1	114.0	1.8	100.6	6.1	85.3	17.4	98.0	9.5	95.4	10.5
182	Myclobutanil	0.001	0.003	1.0000	93.3	101.9	3.2	113.3	4.1	108.4	1.3	92.5	9.3	93.8	4.5	92.6	4.8
183	Nitrothal-isopropyl	0.001	0.003	0.9979	135.8	119.7	4.1	110.7	1.3	104.7	3.4	102.5	7.0	92.6	6.9	96.2	5.1
184	Nonachlor-*cis*	0.001	0.003	1.0000	27.9	96.0	5.5	110.2	2.3	107.0	2.3	89.1	18.2	90.5	14.4	91.4	5.1
	Nonachlor-*trans*	0.001	0.004	1.0000	38.5	88.8	2.4	112.8	3.3	107.8	2.8	96.6	9.0	94.3	7.9	90.6	8.5

No.	Analyte	LOD	LOQ	R^2	ME.(%)	Repeatability (*n*=5)						Reproducibility (*n*=11)					
						0.01 mg kg^{-1}		0.02 mg kg^{-1}		0.1 mg kg^{-1}		0.01 mg kg^{-1}		0.02 mg kg^{-1}		0.1 mg kg^{-1}	
						Rec. (%)	CV (%)	Rec. (%)	CV (%)	Rec. (%)	CV (%)	Rec. (%)	CV (%)	Rec. (%)	CV (%)	Rec. (%)	CV (%)
185	Nuarimol	0.001	0.003	0.9997	206.5	91.0	2.8	105.6	5.7	100.1	1.9	90.5	17.5	90.2	7.2	89.9	4.8
186	Ortho-phenyl phenol	0.002	0.006	0.9998	218.6	102.1	3.3	119.1	5.7	102.9	8.2	97.8	23.4	93.4	11.5	94.0	9.3
187	Oxadiazon	0.001	0.003	1.0000	72.9	102.3	2.6	112.8	3.2	108.1	1.7	94.0	4.9	97.0	3.2	92.6	4.7
188	Oxadixyl	0.001	0.004	0.9998	70.4	107.3	4.1	110.9	4.3	106.0	1.5	94.7	4.6	95.1	4.6	93.4	5.3
189	Oxyfluorfen	0.001	0.004	0.9993	168.3	119.6	7.0	108.9	3.5	106.8	1.5	94.0	3.2	96.5	9.7	100.5	2.3
190	Paclobutrazol	0.002	0.005	0.9997	374.4	109.3	3.6	106.5	5.1	101.4	2.0	92.1	11.2	93.1	3.4	91.2	5.0
191	Parathion	0.001	0.004	0.9994	138.8	119.3	4.6	111.4	1.7	105.2	3.1	92.5	4.6	90.5	6.0	95.0	4.3
192	Parathion-methyl	0.001	0.004	0.9991	164.2	119.7	7.3	105.8	1.7	99.4	4.1	89.6	9.8	89.8	10.0	90.3	3.3
193	Penconazole	0.001	0.003	0.9999	62.7	99.1	2.4	109.3	2.7	105.3	1.8	93.5	7.2	93.4	2.8	90.9	4.3
194	Pendimethalin	0.001	0.003	0.9991	101.7	119.9	4.4	108.3	2.8	103.5	2.8	96.0	4.8	91.9	4.8	94.1	3.8
195	Penflufen	0.001	0.004	1.0000	197.9	102.9	3.6	108.6	5.0	105.2	1.0	89.2	11.0	93.3	2.3	92.1	4.7
196	Pentachlorobenzonitrile	0.001	0.003	0.9996	58.3	108.0	3.4	115.7	1.6	108.8	7.3	90.3	4.8	92.1	4.4	91.0	8.7
197	Penthiopyrad	0.001	0.004	0.9995	245.3	109.3	3.2	108.5	4.6	105.0	1.0	91.4	8.4	94.9	2.0	93.8	4.6
198	Pentoxazone	0.001	0.003	0.9999	141.6	112.2	2.8	107.0	5.0	102.6	1.5	92.8	6.4	94.6	1.6	91.6	3.8
199	Permethrin-cis	0.003	0.008	0.9999	298.9	98.4	8.2	111.6	8.5	106.7	1.3	101.9	12.4	97.4	8.7	91.2	5.0
	Permethrin-trans	0.001	0.003	0.9994	237.1	85.1	7.3	104.8	5.3	102.3	1.3	93.9	7.3	95.4	1.8	91.2	3.6
200	Perthane	0.001	0.003	0.9999	66.9	94.8	2.9	111.8	3.8	106.8	2.4	93.9	4.3	96.3	3.9	92.5	5.4
201	Phenthoate	0.001	0.003	0.9992	106.9	118.9	3.1	109.8	2.5	104.1	2.0	88.5	8.6	91.1	0.9	90.5	1.9
202	Phosphamidon (*E*)	0.003	0.009	0.9998	>500	103.3	28.0	99.4	4.7	89.6	0.5	87.0	7.0	89.0	18.5	87.9	7.5
	Phosphamidon (*Z*)	0.001	0.004	0.9993	433.4	112.3	14.2	90.6	8.0	74.0	2.2	88.4	7.1	87.7	16.2	82.8	5.7
203	Phosalone	0.001	0.004	0.9996	432.7	116.6	9.3	91.1	4.7	83.7	4.8	81.1	24.3	85.1	12.9	83.5	11.3
204	Phosmet	0.001	0.004	0.9998	>500	105.2	8.1	92.5	3.7	83.2	3.3	80.5	16.1	86.5	10.4	89.1	13.7
205	Picoxystrobin	0.001	0.003	0.9999	133.0	96.5	3.4	118.5	3.5	111.5	2.2	91.7	6.0	95.5	3.5	92.6	5.0

No.	Analyte	LOD	LOQ	R^2	ME.(%)	Repeatability (n=5)						Reproducibility (n=11)					
						0.01 mg kg^{-1}		0.02 mg kg^{-1}		0.1 mg kg^{-1}		0.01 mg kg^{-1}		0.02 mg kg^{-1}		0.1 mg kg^{-1}	
						Rec. (%)	CV (%)	Rec. (%)	CV (%)	Rec. (%)	CV (%)	Rec. (%)	CV (%)	Rec. (%)	CV (%)	Rec. (%)	CV (%)
206	Piperonyl butoxide	0.001	0.003	0.9999	292.3	112.1	3.3	108.4	4.7	102.0	1.3	96.6	2.7	96.6	3.2	94.3	5.2
207	Pirimicarb	0.001	0.004	0.9998	88.1	90.6	3.0	112.3	1.2	105.5	5.1	94.0	3.8	94.5	1.8	92.7	4.4
208	Pirimiphos-ethyl	0.001	0.003	0.9985	83.1	105.3	4.0	107.9	1.3	105.5	3.4	94.4	4.4	95.9	2.1	93.9	3.3
209	Pirimiphos-methyl	0.001	0.003	0.9990	97.1	95.1	2.0	107.2	3.0	103.8	3.5	97.3	8.2	93.0	3.4	90.0	3.9
210	Pretilachlor	0.001	0.003	1.0000	116.9	111.5	3.9	113.6	3.8	107.1	1.8	97.5	12.4	97.8	2.1	93.2	3.3
211	Prochloraz	0.002	0.005	0.9994	>500	108.5	5.0	106.7	5.4	100.5	0.4	78.2	12.0	85.0	3.8	91.8	12.4
	2,4,6-trichlorophenol	0.003	0.009	0.9981	123.8	164.3	6.1	106.1	6.0	93.3	10.0	75.7	3.2	77.8	2.7	103.9	42.1
212	Procymidone	0.003	0.009	0.9999	51.4	94.6	2.7	117.2	3.5	107.2	2.6	88.8	3.4	90.7	2.0	91.9	8.7
213	Prodiamine	0.001	0.003	0.9992	88.6	108.7	1.7	109.3	3.1	105.3	4.4	95.0	6.6	94.8	4.6	93.9	1.9
214	Profenofos	0.002	0.005	0.9999	379.1	118.4	11.2	104.7	3.5	96.5	1.8	82.5	15.0	88.0	8.9	83.6	8.4
215	Profluralin	0.001	0.004	0.9992	57.8	101.7	2.3	110.9	1.9	104.8	8.0	102.0	7.0	97.7	7.8	98.9	2.8
216	Prohydrojasmon	0.002	0.006	0.9982	115.9	86.1	2.3	98.8	3.8	100.3	6.4	89.8	5.7	90.8	4.4	91.3	7.2
217	Prometon	0.001	0.003	0.9996	107.2	91.1	3.0	111.5	1.1	103.0	5.9	95.7	5.0	98.2	8.8	98.7	3.6
218	Prometryn	0.001	0.003	0.9992	82.1	87.1	1.6	108.0	2.3	101.8	3.5	90.2	7.4	90.3	4.0	90.2	5.9
219	Propachlor	0.002	0.005	0.9997	73.6	105.5	4.0	116.8	2.4	103.8	10.3	93.3	3.8	93.8	3.9	92.9	6.6
220	Propanil	0.001	0.004	0.9993	135.9	96.4	3.5	104.8	7.9	102.7	3.3	98.4	10.9	96.6	11.0	95.4	3.4
221	Propazine	0.001	0.004	0.9998	72.0	94.6	1.7	112.0	0.6	104.8	5.4	94.8	7.2	93.8	3.3	91.4	5.7
222	Propetamphos	0.001	0.004	0.9996	91.9	99.2	3.2	109.0	3.6	104.7	6.5	91.0	12.0	92.4	5.3	91.6	6.0
223	Propham	0.002	0.006	0.9998	114.0	89.3	4.1	114.8	1.9	100.2	12.3	91.5	4.6	97.1	9.5	95.3	9.5
224	Propiconazole-1	0.001	0.004	0.9998	130.9	97.6	2.3	110.9	3.2	105.1	0.1	92.7	11.1	92.7	1.7	91.8	3.7
	Propiconazole-2	0.002	0.005	0.9998	149.3	93.1	2.8	110.1	5.4	103.9	2.5	94.5	8.9	94.7	0.2	92.9	2.7
225	Propisochlor	0.001	0.003	0.9981	65.9	100.8	3.3	108.1	2.4	103.6	4.8	91.4	5.3	93.9	0.2	91.0	7.8
226	Propyzamide	0.001	0.004	0.9983	103.6	95.6	2.6	113.4	2.0	106.5	4.1	91.4	7.3	93.0	1.9	92.4	6.1

No.	Analyte	LOD	LOQ	R^2	ME.(%)	Repeatability (n=5)						Reproducibility (n=11)					
						0.01 mg kg^{-1}		0.02 mg kg^{-1}		0.1 mg kg^{-1}		0.01 mg kg^{-1}		0.02 mg kg^{-1}		0.1 mg kg^{-1}	
						Rec. (%)	CV (%)	Rec. (%)	CV (%)	Rec. (%)	CV (%)	Rec. (%)	CV (%)	Rec. (%)	CV (%)	Rec. (%)	CV (%)
227	Prothiofos	0.001	0.004	0.9999	119.1	96.3	2.8	112.8	3.5	107.6	2.2	88.4	8.1	92.1	5.9	89.3	6.5
228	Pyracarbolid	0.002	0.005	0.9995	158.2	105.1	3.1	112.5	4.6	107.2	1.9	103.1	14.1	99.8	8.9	94.4	2.4
229	Pyraclofos	0.002	0.006	0.9997	>500	98.7	8.2	92.7	5.9	80.8	3.1	78.1	9.7	86.9	4.3	87.3	11.5
230	Pyraflufen-ethyl	0.001	0.004	1.0000	176.8	104.3	3.5	107.3	5.0	103.0	1.3	91.5	9.2	93.4	3.7	92.1	5.0
231	Pyrazophos	0.001	0.004	0.9991	332.1	113.4	7.8	102.1	4.3	96.5	0.6	86.1	15.7	88.0	7.5	88.0	7.0
232	Pyridalyl	0.001	0.003	0.9994	303.2	87.1	3.7	100.0	7.2	99.7	0.8	91.6	3.1	90.2	5.3	90.5	3.2
233	Pyrifenox	0.001	0.004	0.9998	100.4	91.1	2.7	108.8	2.3	103.2	1.5	91.1	10.2	91.3	3.5	89.5	7.3
234	Pyriftalid	0.001	0.004	0.9994	278.4	104.4	4.2	105.5	4.7	102.1	1.5	92.4	11.0	94.3	5.8	91.2	1.8
235	Pyrimethanil	0.001	0.004	1.0000	80.1	90.4	2.2	111.0	0.9	103.7	5.4	91.2	9.5	91.7	4.2	91.1	7.6
236	Pyriminobac-methyl (*E*)	0.001	0.004	1.0000	167.4	103.7	3.6	114.4	4.6	107.7	2.0	93.4	7.9	93.7	1.1	94.2	3.8
	Pyriminobac-methyl (*Z*)	0.001	0.003	0.9997	84.4	101.3	2.4	110.9	4.4	108.1	1.7	91.9	7.1	94.7	2.5	93.4	4.9
237	Quinalphos	0.001	0.004	0.9997	92.8	110.1	3.3	110.2	3.7	104.4	1.5	92.4	10.4	97.6	9.4	90.0	0.4
238	Quinoxyfen	0.001	0.003	1.0000	63.8	87.6	2.7	108.6	4.9	103.8	2.1	87.9	9.7	90.5	4.3	87.9	4.2
239	Quintozene	0.001	0.004	0.9987	60.5	119.8	6.7	106.0	3.7	100.8	9.0	94.5	12.0	92.2	10.1	89.5	7.7
240	Quizalofop-ethyl	0.001	0.003	0.9982	303.7	101.0	4.3	104.9	5.9	103.1	0.8	88.1	11.1	94.2	0.5	92.7	0.7
241	Silafluofen	0.001	0.003	0.9989	185.9	87.5	3.1	101.0	5.3	100.8	1.1	93.7	3.5	93.2	4.1	90.5	4.2
242	Simeconazole	0.001	0.004	0.9986	138.7	95.8	2.6	106.6	3.2	103.8	4.4	92.3	8.0	92.5	3.0	92.8	5.8
243	Simetryn	0.001	0.004	0.9994	95.0	91.5	2.4	108.4	2.0	102.8	3.1	93.5	2.3	91.8	1.5	92.2	1.9
244	Spiromesifen	0.001	0.004	0.9999	207.9	104.6	9.8	106.3	5.1	99.3	0.9	81.1	15.6	87.9	4.2	86.7	4.9
245	Spiroxamine-1	0.002	0.005	0.9983	139.5	85.0	5.7	94.2	6.0	84.7	5.9	95.6	6.0	92.9	2.6	98.2	6.4
	Spiroxamine-2	0.002	0.005	0.9987	94.8	86.8	3.8	95.9	2.9	87.2	4.7	94.3	9.7	95.6	9.5	102.8	10.5
246	Sulfotep	0.001	0.004	0.9996	54.3	94.4	2.9	111.4	2.5	103.2	8.1	94.4	5.1	96.6	3.6	94.6	5.1
247	Tebuconazole	0.002	0.005	0.9999	356.6	99.9	3.1	108.1	4.7	100.1	1.4	86.6	9.3	90.6	1.6	92.0	4.4

No.	Analyte	LOD	LOQ	R^2	ME.(%)	Repeatability (*n*=5)						Reproducibility (*n*=11)					
						0.01 mg kg^{-1}		0.02 mg kg^{-1}		0.1 mg kg^{-1}		0.01 mg kg^{-1}		0.02 mg kg^{-1}		0.1 mg kg^{-1}	
						Rec. (%)	CV (%)	Rec. (%)	CV (%)	Rec. (%)	CV (%)	Rec. (%)	CV (%)	Rec. (%)	CV (%)	Rec. (%)	CV (%)
248	Tebufenpyrad	0.001	0.003	0.9997	166.9	89.9	3.0	107.3	5.5	102.2	1.2	93.5	6.1	94.0	0.6	92.2	2.1
249	Tebupirimfos	0.001	0.004	0.9997	74.4	91.6	2.2	113.3	2.9	106.1	5.7	96.4	3.3	95.5	2.8	94.2	6.2
250	Tecnazene	0.002	0.005	0.9997	60.2	113.4	4.9	113.3	3.8	95.1	11.5	94.8	7.0	93.5	7.0	93.9	9.3
251	Tefluthrin	0.001	0.004	0.9997	33.5	89.1	1.9	109.0	2.1	108.0	6.1	96.8	5.2	96.7	4.5	95.2	6.8
252	Terbacil	0.002	0.005	0.9991	272.4	109.6	3.7	104.3	3.7	98.2	3.4	94.6	8.5	92.7	6.1	91.8	3.6
253	Terbumeton	0.001	0.004	0.9984	100.0	92.8	4.1	111.2	0.9	103.9	5.7	97.2	8.3	94.0	2.2	94.6	1.3
254	Terbutryn	0.001	0.004	0.9989	85.9	89.8	2.9	108.4	2.1	102.6	2.9	93.1	5.6	91.2	5.1	89.2	4.9
255	Tetrachlorvinphos	0.001	0.004	0.9999	394.5	93.4	9.6	93.4	3.9	83.4	0.6	83.0	6.0	85.8	16.5	79.8	7.0
256	Tetraconazole	0.001	0.004	0.9999	84.2	99.9	3.4	115.0	2.8	108.8	2.0	95.1	6.1	94.3	2.4	92.7	5.0
257	Tetradifon	0.001	0.003	0.9999	68.3	100.1	2.8	110.2	4.8	105.8	2.3	89.7	8.0	91.4	4.6	89.5	4.2
258	Tetramethrin-1	0.001	0.004	1.0000	468.5	118.8	12.9	110.3	6.3	97.4	1.1	151.4	70.4	92.8	1.3	92.3	0.3
	Tetramethrin-2	0.003	0.008	0.9999	330.0	89.3	15.2	112.5	5.3	104.2	1.2	84.4	40.3	70.4	6.5	78.2	1.7
259	Thifluzamide	0.001	0.004	0.9999	293.5	115.4	9.3	111.9	4.5	108.2	1.7	91.1	10.4	95.0	3.2	93.5	5.4
260	Thiometon	0.002	0.005	0.9995	80.9	83.8	5.1	113.2	2.1	96.2	2.1	81.0	20.8	85.0	13.2	90.3	13.7
261	Thionazin	0.003	0.009	0.9995	66.0	90.4	3.8	117.4	2.9	103.6	10.1	94.3	5.2	93.2	4.8	93.2	7.8
262	Tolclofos-methyl	0.001	0.004	0.9981	71.8	92.9	3.1	114.7	1.8	108.3	4.9	92.0	5.3	94.2	2.9	90.7	6.9
263	Triadimefon	0.001	0.004	0.9996	78.5	102.4	3.0	114.9	3.0	109.0	2.8	94.6	3.8	93.2	1.7	92.9	4.8
264	Triadimenol	0.002	0.006	0.9997	180.9	93.0	3.0	106.7	4.9	101.1	2.5	90.4	10.9	91.5	3.3	95.9	1.6
265	Tri-allate	0.001	0.003	0.9995	59.8	88.8	1.3	113.0	2.0	108.6	6.1	91.8	7.7	92.9	5.2	92.3	7.1
266	Triazophos	0.001	0.004	0.9996	273.1	114.0	8.8	105.8	4.2	99.6	1.4	83.8	20.9	90.0	4.1	88.7	5.1
267	Tridiphane	0.001	0.004	0.9977	59.6	104.0	10.7	106.0	2.7	102.4	5.0	93.6	13.5	88.0	12.0	95.5	11.8
268	Trifloxystrobin	0.001	0.003	0.9999	119.6	110.2	1.8	110.2	4.4	105.7	1.4	94.8	7.4	95.8	1.7	94.9	2.8
269	Triflumizole	0.001	0.004	0.9997	94.9	98.0	3.5	104.2	1.5	99.7	3.2	90.6	2.7	90.8	1.7	90.2	1.3

No.	Analyte	LOD	LOQ	R^2	ME.(%)	Repeatability (*n*=5)						Reproducibility (*n*=11)					
						0.01 mg kg^{-1}		0.02 mg kg^{-1}		0.1 mg kg^{-1}		0.01 mg kg^{-1}		0.02 mg kg^{-1}		0.1 mg kg^{-1}	
						Rec. (%)	CV (%)	Rec. (%)	CV (%)	Rec. (%)	CV (%)	Rec. (%)	CV (%)	Rec. (%)	CV (%)	Rec. (%)	CV (%)
270	Trifluralin	0.001	0.004	0.9995	60.6	100.0	1.8	111.2	3.5	104.1	9.3	98.3	4.3	97.5	3.8	99.7	6.1
271	Vinclozolin	0.001	0.003	0.9990	56.0	103.0	2.9	115.0	2.6	107.4	3.5	91.2	8.0	93.2	4.4	93.3	10.2
272	Zoxamide	0.002	0.005	0.9998	>500	98.6	11.8	86.9	14.1	79.6	11.9	97.9	9.2	97.7	12.7	83.8	15.8
